# Nanoscale Distribution of Presynaptic Ca^2+^ Channels and Its Impact on Vesicular Release during Development

**DOI:** 10.1016/j.neuron.2014.11.019

**Published:** 2015-01-07

**Authors:** Yukihiro Nakamura, Harumi Harada, Naomi Kamasawa, Ko Matsui, Jason S. Rothman, Ryuichi Shigemoto, R. Angus Silver, David A. DiGregorio, Tomoyuki Takahashi

**Affiliations:** 1 Laboratory of Molecular Synaptic Function, Graduate School of Brain Science, Doshisha University, Kyoto 610-0394, Japan; 2 Cellular & Molecular Synaptic Function Unit, Okinawa Institute of Science and Technology (OIST) Graduate University, Okinawa 904-0495, Japan; 3 Laboratory of Dynamic Neuronal Imaging, Institut Pasteur, 25 rue du Dr Roux, 75724 Paris Cedex 15, France; 4 CNRS UMR 3571, 25 rue du Dr Roux, 75724 Paris Cedex 15, France; 5 Division of Cerebral Structure, Department of Cerebral Research, National Institute for Physiological Sciences, Myodaiji, Okazaki 444-8787, Japan; 6 Institute of Science and Technology Austria, A-3400 Klosterneuburg, Austria; 7 Department of Neuroscience, Physiology and Pharmacology, University College London, Gower Street London WC1E 6BT, UK

## Abstract

Synaptic efficacy and precision are influenced by the coupling of voltage-gated Ca^2+^ channels (VGCCs) to vesicles. But because the topography of VGCCs and their proximity to vesicles is unknown, a quantitative understanding of the determinants of vesicular release at nanometer scale is lacking. To investigate this, we combined freeze-fracture replica immunogold labeling of Ca_v_2.1 channels, local [Ca^2+^] imaging, and patch pipette perfusion of EGTA at the calyx of Held. Between postnatal day 7 and 21, VGCCs formed variable sized clusters and vesicular release became less sensitive to EGTA, whereas fixed Ca^2+^ buffer properties remained constant. Experimentally constrained reaction-diffusion simulations suggest that Ca^2+^ sensors for vesicular release are located at the perimeter of VGCC clusters (<30 nm) and predict that VGCC number per cluster determines vesicular release probability without altering release time course. This “perimeter release model” provides a unifying framework accounting for developmental changes in both synaptic efficacy and time course.

## Highlights

• Ca^2+^ channels form clusters with highly variable numbers of channels
• EGTA sensitivity suggests that synaptic vesicles are tightly coupled to clusters
• Ca^2+^ channel number per cluster alters synaptic efficacy, but not precision
• A perimeter model accounts for synaptic efficacy and precision during development

## Teaser

Nanoscale topography of presynaptic Ca^2+^ channels and synaptic vesicles critically influences synaptic transmission. Nakamura et al. find that action potentials evoke release of vesicles coupled to the perimeter of Ca^2+^ channel clusters, explaining synaptic efficacy and precision during development.

## Introduction

Fast and precise chemical synaptic transmission is thought to be achieved through the colocalization of voltage-gated Ca^2+^ channels (VGCCs) and release-ready synaptic vesicles at the presynaptic active zone (AZ) (Eggermann et al., 2012). However, the effect on release of exogenous calcium buffers, such as EGTA, suggests that the “coupling” distance between VGCCs and the Ca^2+^ sensor for vesicular release (VGCC-sensor distance) varies across mammalian synapses producing either “loose” (Rozov et al., 2001; Fedchyshyn and Wang, 2005; Vyleta and Jonas, 2014) or “tight” coupling (Mintz et al., 1995; Fedchyshyn and Wang, 2005; Bucurenciu et al., 2008; Schmidt et al., 2013). Detailed simulations of Ca^2+^ buffering and diffusion indicate that the efficacy and time course of vesicular release can be sensitive to differences in the VGCC-sensor distance as small as 5–10 nm (Bennett et al., 2000; Meinrenken et al., 2002; Bucurenciu et al., 2008; Wang et al., 2009; Scimemi and Diamond, 2012). However, the ability of such simulations to reproduce the amplitude and time course of action potential (AP)-evoked vesicular release is limited, since key model parameters have not been experimentally measured. These parameters include knowledge of the spatial distributions of VGCCs and Ca^2+^ sensors, as well as intracellular Ca^2+^ buffering properties.

Lack of information on the spatial arrangement of VGCCs and synaptic vesicles within the AZ has led to divergent models of synaptic release, ranging from clustered VGCCs with random vesicle placement (Meinrenken et al., 2002; Ermolyuk et al., 2013) to random placement of both VGCCs and vesicles (Scimemi and Diamond, 2012). Recent advances in Ca^2+^ channel antibodies and freeze-fracture replica labeling electron microscopy (EM) have established that VGCCs form clusters at the AZ of central mammalian synapses (Kulik et al., 2004; Bucurenciu et al., 2008; Holderith et al., 2012; Indriati et al., 2013), but the number, density, and distribution of VGCCs within these clusters and their influence on vesicular release have not been explored. Indeed, estimates for the number of VGCCs necessary to drive vesicular release range from 1 (Stanley, 1993), to several (Fedchyshyn and Wang, 2005; Bucurenciu et al., 2010; Scimemi and Diamond, 2012), or to >10 (Borst and Sakmann, 1996; Nadkarni et al., 2010; Sheng et al., 2012).

To understand how the spatial distribution of VGCCs affect the VGCC-sensor coupling, we studied the calyx of Held synapse, since many of its properties are well characterized, and it is particularly amenable to presynaptic imaging and whole-cell patch pipette perfusion with exogenous buffers. By combining functional measurements, freeze-fracture replica immunogold labeling of Ca_v_2.1 channels, and experimentally constrained 3D models of Ca^2+^ diffusion and vesicular release, we estimated VGCC-sensor distance at different stage of development. Model predictions were tested against measurements of the sensitivity of vesicular release to EGTA, vesicular release probability, and the time course of release. Our results suggest that the Ca^2+^ sensors for vesicular release are located close to the perimeter of VGCC clusters. Moreover, our findings reconcile apparent inconsistencies across various experimental findings and explain how the speed and efficacy of AP-evoked vesicular release is differentially modulated during development.

## Results

### Clustering of Ca_v_2.1 Subunits at the Calyx of Held at Different Developmental Stages

**Figure 1:**
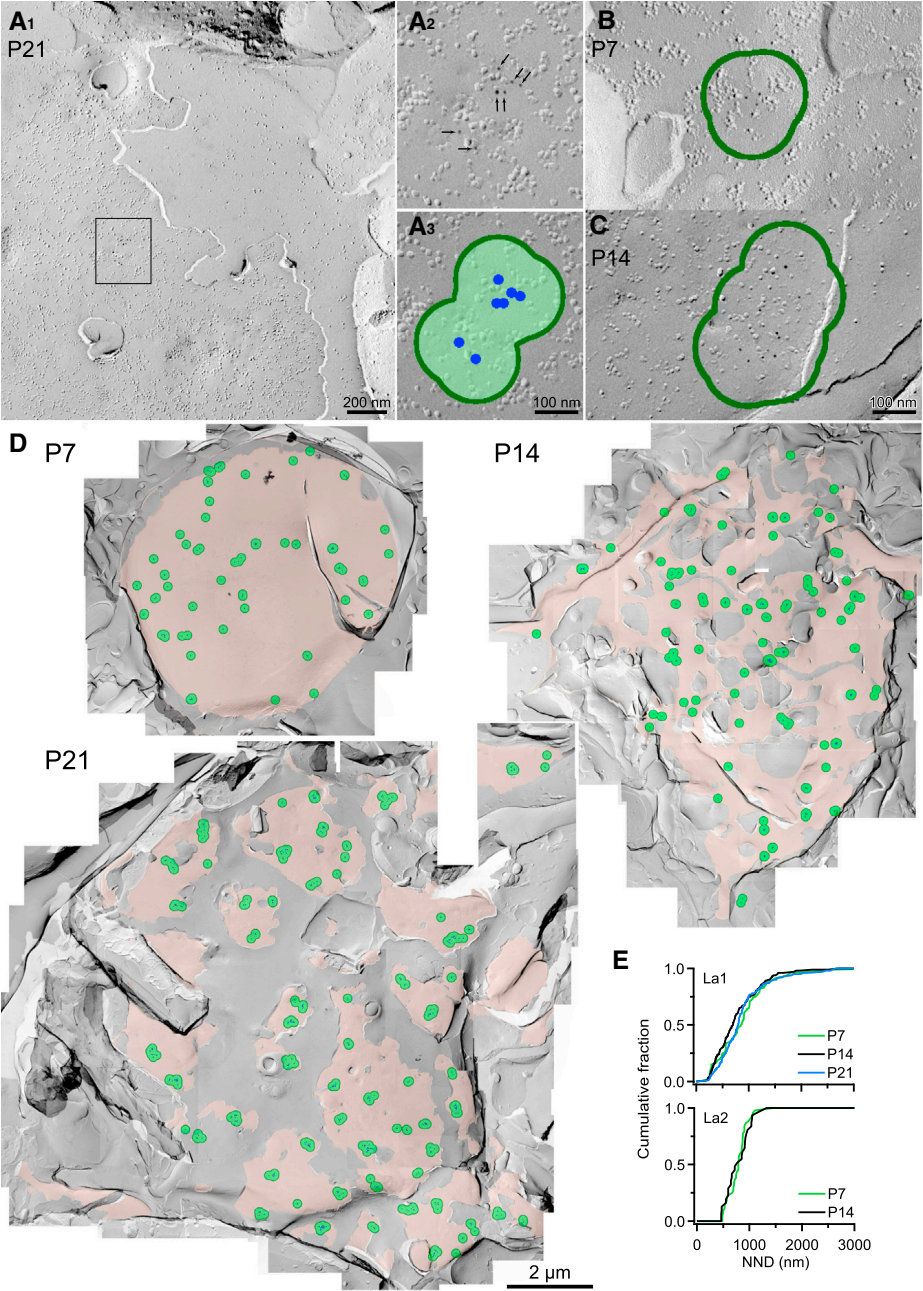
Distribution of Ca_v_2.1 Immunoparticles at the Developing Calyx of Held Presynaptic Terminal Revealed with SDS-FRL (A1) Freeze-fracture replica image of a calyx of Held of a P21 rat. Presynaptic P-face classification is confirmed by the presence of convex structures in the cross-fractured face that are likely to reflect synaptic vesicles. Round, electron-dense particles (5 nm diameter) indicate immunogold-labeled Ca_v_2.1 antibodies, which are often seen in characteristic concaved surface with dimples (Figures S2A and S2B). The higher magnification of this image is shown in Figure S2A. (A2) Zoom of box region in (A1). Arrows indicate 5 nm immunogold particles. (A3) Clusters of two or more particles were identified by the overlap of 100 nm radius circles (green) centered on the particles (blue). (B) Typical immunogold particle cluster at P7. (C) Typical immunogold particle cluster at P14. (D) Low magnification of immunogold particle clusters (green) in the presynaptic P-face (pink) of calyces of Held at different ages. (E) Cumulative histograms of NND between cluster centers comparing different ages in La1 (upper panel, p = 0.03 for P7 versus, P14, p = 0.27 for P7 versus, P21, p = 0.008 for P14 versus, P21) and La2 (lower panel, p = 0.26 for P7 versus, P14). Statistical comparisons were performed using a Kolmogorov-Smirnov test. La1 and La2 indicate samples reacted with different antibody batches resulting in a higher efficiency labeling for La2 (62% versus 19% for La1). All images are taken from La1 samples.

The calyx of Held synapse undergoes both morphological and functional changes during the second postnatal week, when rodents start to hear sounds (Kandler and Friauf, 1993; Taschenberger et al., 2002). To examine whether these functional changes are associated with alterations in the VGCC distribution, we performed SDS-digested freeze-fracture replica labeling (SDS-FRL) (Fujimoto, 1995; Hagiwara et al., 2005) with an antibody specific for the Ca_v_2.1 subunit of P/Q-type VGCCs (Holderith et al., 2012; Miyazaki et al., 2012; Indriati et al., 2013). Large continuous membranes with abundant intramembrane particles and shallow convex structures were identified as the presynaptic protoplasmic face (P-face) of a calyx when it adjoined the cross-fractured face through the presynaptic terminal containing synaptic vesicles. As expected from the intracellular location of the epitope, immunogold particles (5 nm diameter) for Ca_v_2.1 were predominantly found on the presynaptic P-face, with little labeling on the exoplasmic face or cross-fractured face (Figure 1A). Specificity of the antibody in replica labeling was confirmed using Ca_v_2.1 knockout mice, which showed little Ca_v_2.1 labeling (Figures S1A and S1B available online). Averaging over the entire P-face of calyx presynaptic membrane produced a mean density of immunogold particles of 2.6/μm^2^ at P7, 6.7/μm^2^ at P14, and 4.6/μm^2^ at P21 in one set of labeling samples (La1). In another set of labeling samples using a different batch of antibodies (La2), a higher particle density was observed (8.6/μm^2^ at P7 and 21.7/μm^2^ at P14).

Because the gold particles appeared to form clusters, we tested this possibility by comparing the gold particle distribution with a randomly distributed particle model (Figures S1C–S1F). To define a cluster, we drew a circle of various radii (50–500 nm) around each particle and compared the “cluster rate” between random and real particle distributions (for details, see Figure S1F legend). When the circles overlapped, particles were assigned to a single cluster (Figures 1A_3_, S2A, and S2C). We found that the cluster rate of the real particle distribution relative to the random distribution was highest when the radius was 100 nm. We therefore used this radius along with the condition that two or more gold particles are located within the encircled area as our definition of a cluster. Using this criterion, most gold particles (>80%, La1; >97%, La2) were located within these clusters regardless of developmental stage (Figures 1B–1D).

Ca_v_2.1 labeling followed by RIM staining revealed that the majority of Ca_v_2.1 clusters were closely associated with RIM particles (64% at P7, 87% at P14, and 74% at P21, Figures S2C and S2D), supporting the idea that Ca_v_2.1 clusters are localized in AZs. The nearest neighbor distance (NND) between clusters was similar, but not identical, across ages (La1): 899 ± 60 nm for P7, 779 ± 32 nm for P14, and 880 ± 38 nm for P21 (Figure 1E). Similar values were observed in La2 for P7 (785 ± 29 nm, n = 44) and P14 (787 ± 41 nm, n = 31). These intercluster NNDs are longer than that estimated for AZs at the P9 calyx (Sätzler et al., 2002) but comparable to those at the P14 calyx (940 nm; calculated from Taschenberger et al., 2002). The similar NNDs for Ca_v_2.1 clusters and AZs suggest that most AZs contain a single cluster of Ca_v_2.1 channel.

### Quantification of the Spatial Distribution of Ca_v_2.1 Subunits within Clusters

**Figure 2:**
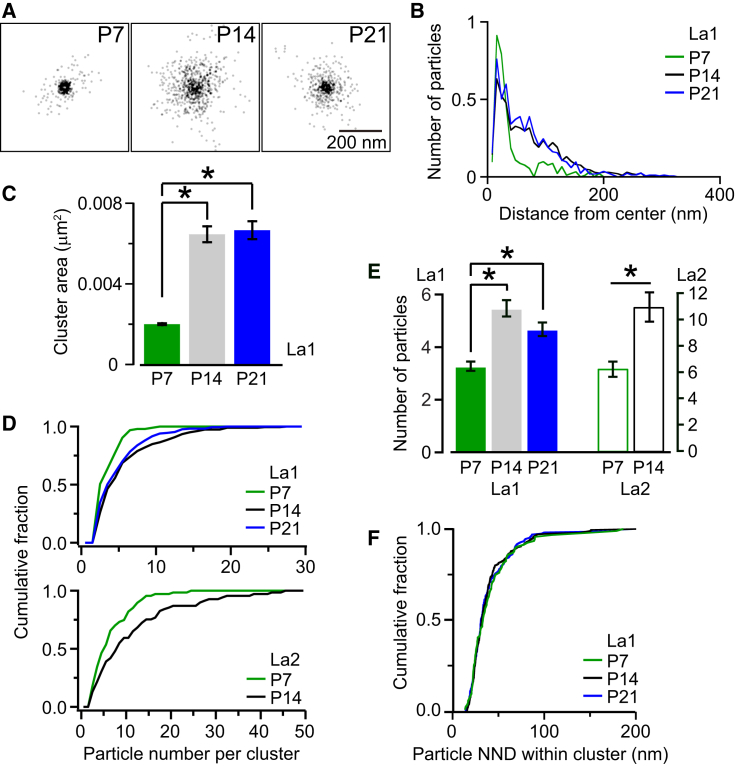
Ca_v_2.1 Immunogold Particle Distribution within Clusters as a Function of Age (A) Overlaid gold particle clusters (La1) aligned at their center of gravity (92 clusters per age). (B) Spatial distribution profiles of immunogold particles showing particle distances from the center of gravity of its corresponding cluster. Ordinate indicates number of particles in concentric bins (8 nm). (C) Mean cluster area (± SEM) for La1. Only clusters having three or more particles were included in this analysis (^∗^p < 0.01, one way ANOVA followed by Tukey's post hoc). (D) Cumulative histograms of immunogold particle number per cluster from La1 (upper) and La2 (lower). (E) Mean number (± SEM) of immunogold particles within a cluster for La1 (filled bars, ^∗^p < 0.01, one-way ANOVA followed by Tukey's post hoc) and La2 (open bars, ^∗^p < 0.01, t test). (F) Cumulative histograms of intracluster particle NND values for La1.

In order to evaluate the local distribution of Ca_v_2.1 immunogold particles within clusters, we superimposed 92 VGCC cluster profiles aligned at their center (Figure 2A) and plotted the density function from the cluster center (Figure 2B). Particles at P7 were more spatially confined than those after P14. Cluster area, computed from a perimeter line drawn through the outermost gold particles of a cluster, increased from P7 (0.0020 μm^2^, n = 46) to P14 (0.0065 μm^2^, n = 146) and then remained constant for P21 (0.0067 μm^2^, n = 105, Figure 2C). The Ca_v_2.1 cluster area at P14 corresponds to 12% of the AZ area estimated previously (Taschenberger et al., 2002).

The number of particles per cluster in La1 samples varied over a wide range, from 2 to 27 (Figure 2D) with an average of 3.1 ± 0.3 at P7 (n = 93 clusters), 5.3 ± 0.8 at P14 (n = 199), and 4.7 ± 0.3 at P21 (n = 167, Figure 2E). In La2, the number was approximately 2-fold larger ranging from 2 to 45 with a mean of 6.4 ± 0.6 at P7 (n = 69) and 11.1 ± 1.2 at P14 (n = 67), a similar age ratio to that in La1. In contrast, the NND between particles within a cluster remained similar from P7 (28 ± 1 nm) to P14 (30 ± 1 nm) in La2. The NND in La1 (39 ± 2 nm for P7, 37 ± 1 nm for P14, and 37 ± 1 nm for P21) was longer than those in La2, as expected for less efficient labeling (Experimental Procedures), and was similar throughout development as for La2 (Figure 2F, p > 0.8, Kolmogorov Smirnov test). The distribution of Ca_v_2.1 NND was narrow, with 80% of the NNDs between 13 and 55 nm (La1). These data indicate that the density of Ca_v_2.1 remains similar from P7 to P21, but the number of Ca_v_2.1 per cluster and the cluster area increase with development from P7 to P14.

### Presynaptic Ca^2+^ Dynamics Evoked by Single APs at Different Developmental Stages

**Figure 3:**
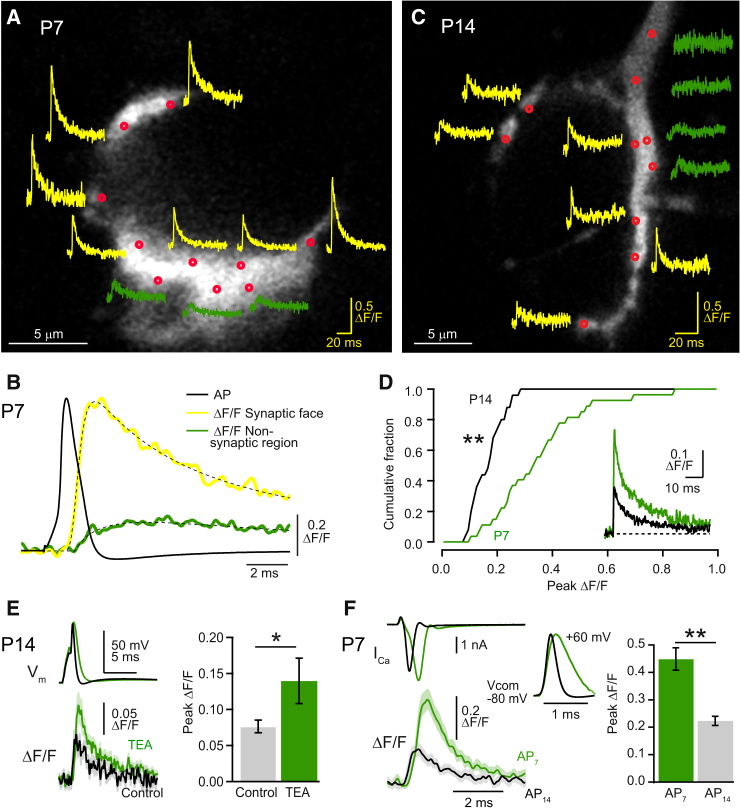
Developmental Reduction of AP-Induced Ca^2+^ Transients Measured with Confocal Spot Detection (A) Red channel confocal image of a P7 calyx of Held loaded with Alexa 594 and Oregon green BAPTA-5N. Single AP-induced Ca^2+^ transients are presented as ΔF/F and were recorded at various locations within the terminal (red circles) using confocal spot detection. Yellow and green traces show single Ca^2+^ transients recorded from confocal spots on the synaptic face and nonsynaptic regions, respectively. (B) Temporal relationship between the presynaptic AP (black trace) and Ca^2+^ transients recorded from spot locations along the synaptic face (yellow trace, average of seven locations in [A]) or at nonsynaptic regions (green trace, average of three locations in [A]) in a P7 calyx. Dashed line is a fit with Equation 2 (Supplemental Experimental Procedures). (C) Same as (A) but for a P14 calyx. Traces are averages of five trials. (D) Cumulative amplitude histograms of Ca^2+^ transient amplitudes at P7 and P14 (^∗∗^p < 0.01, Kolmogorov-Smirnov test). Inset, the population average at P7 (green, 21 spots from 8 calyces) and P14 (21 spots from 6 calyces). (E) Left: Averaged AP (V_m_) and synaptic-face Ca^2+^ transient in the absence (black; n = 29 traces) or presence (green; n = 21 traces) of 1 to 2 mM TEA (shaded areas denote 2× SEM). Right: Mean peak amplitude (± SEM) of Ca^2+^ transients in control and TEA (P14, n = 7 calyces, ^∗^p < 0.05, paired t test). (F) Left: Whole-terminal I_Ca_ and averaged Ca^2+^ transient (n = 16 traces) evoked in voltage clamp using an AP-waveform recorded previously from P7 (green, AP_7_, inset) and P14 (black, AP_14_, inset) calyces. EGTA concentration was 2 mM. Right: Mean peak amplitudes (± SEM) of Ca^2+^ transients for AP_7_ and AP_14_ at P7 calyces (^∗∗^p < 0.01, paired t test, n = 9 calyces).

In order to examine whether the spatio-temporal profile of presynaptic AP-evoked [Ca^2+^] changes was altered during the hearing acquisition period, we recorded local Ca^2+^ transients in response to single APs using a high-speed confocal spot detection method and low-affinity Ca^2+^ indicator Oregon green BAPTA-5N (DiGregorio et al., 1999). The point spread function of the microscope was 220 nm (*XY* axis) and 650 nm (*Z* axis) (Figure S3). In P7 calyces, Ca^2+^ transients were observed in the majority of locations tested on the synaptic face (79% ± 3%, n = 5 calyces, Figure 3A). Ca^2+^ transients rose rapidly during the repolarization phase of the AP, and their decays exhibited both a fast (2–5 ms) and slow (>10 ms) component, consistent with localized detection of a [Ca^2+^] domain (DiGregorio et al., 1999). In contrast, Ca^2+^ transients were slower and smaller when measured at locations away from the synaptic face (Figures 3B and S4), consistent with the report that VGCC currents were recorded exclusively at the synaptic face (Sheng et al., 2012).

In P14 calyces Ca^2+^ transients were also observed for confocal spot locations along the synaptic face, albeit less frequently than in P7 calyces (44% ± 16%, n = 5 calyces), and exhibited a similar spatial dependence (Figure 3C). To compare the amplitude of Ca^2+^ transients between ages, we selected transients recorded at the synaptic face with rise times less than 0.5 ms. Fast rise times are an indication that the confocal spot is located close (<200 nm) to the Ca^2+^ entry site (Figure S4). The mean peak amplitude of Ca^2+^ transients at P14 (0.17 ± 0.01 ΔF/F, n = 25 spot locationss from six calyces; Figure 3D) was 50% of that at P7 (0.35 ± 0.03 ΔF/F, n = 27 spot locations from eight calyces). To determine whether a developmental decrease of the AP duration underlies the age difference in the Ca^2+^ transient amplitude, we prolonged the AP duration at P14 calyces using 1 to 2 mM tetraethyl ammonium (TEA), thus mimicking the AP measured at P7 (Ishikawa et al., 2003). TEA caused an 83% ± 30% increase in the Ca^2+^ transient amplitude (Figure 3E). In a different set of experiments, we voltage clamped P7 calyces using an AP-waveform voltage command derived from P7 (AP_7_) or P14 (AP_14_) calyces. The shorter duration AP_14_ waveform reduced the time to peak of the Ca^2+^ current (I_Ca_) by 51% ± 1% (n = 9 calyces, p < 0.01, paired t test) as compared to using AP_7_ waveforms. Ca^2+^ transients recorded from the same spot location were 56% smaller when elicited using AP_14_ than when elicited by AP_7_ (Figure 3F). Hence, these results show that the Ca^2+^ influx is restricted to the synaptic face of calyces at both P7 and P14 and that the shortening of the presynaptic AP duration can account for a substantial fraction of the decrease in the Ca^2+^ transient amplitude during development.

### Properties of Endogenous Fixed Buffer

**Figure 4:**
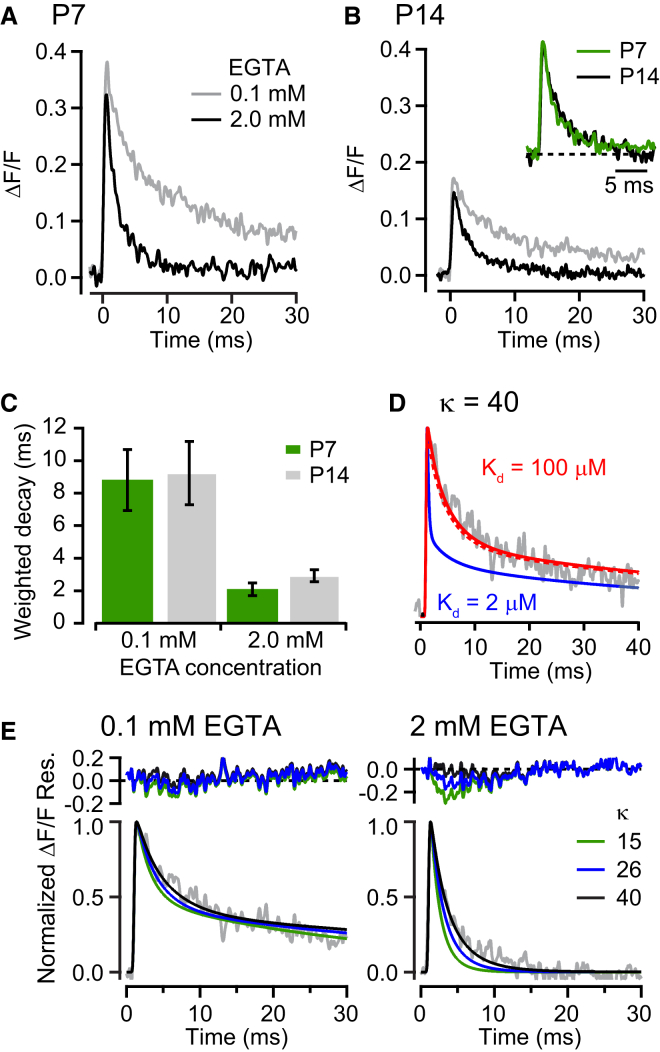
Quantification of Endogenous Fixed Ca^2+^ Buffer Properties at P7 and P14 (A) Averaged Ca^2+^ transient at the synaptic face of P7 calyces in the presence of 0.1 mM (gray, n = 25 calyces) or 2 mM (black, n = 33) EGTA in presynaptic pipettes. (B) Same as (A) but for P14 calyces (n = 23 and 33 respectively). Inset shows normalized Ca^2+^ transients for 2 mM EGTA at P7 (green) and P14 (black) for kinetic comparison. (C) Average weighted mean time constant (± SEM) of the Ca^2+^ transients at P7 and P14. (D) A normalized simulated Ca^2+^ transient computed with a fast EFB k_off_ (1.0 × 10^4^ s^−1^, K_d_ = 100 μM, red trace) matched the experimental Ca^2+^ transient (gray) better than when computed with a slow EFB k_off_ (1.0 × 10^3^ s^−1^, K_d_ = 2 μM, blue). The EFB concentration was adjusted to keep binding capacity κ = 40. (E) Normalized simulated Ca^2+^ transients with a low-affinity EFB (K_d_ = 100 μM) of κ = 15 (green), 26 (blue), and 40 (black) in the presence of 0.1 mM (left) and 2 mM EGTA (right). Normalized experimental Ca^2+^ transients (P14; gray) recorded in 0.1 and 2 mM EGTA are plotted on left and right panels, respectively. Residuals (Res.) were calculated from the difference between the normalized simulation traces and experimental traces.

The properties of endogenous buffers can influence the spatio-temporal profile of the [Ca^2+^] that drives vesicular release (Eggermann et al., 2012). We estimated the properties of endogenous fixed buffers (EFBs) in dialyzed terminals (i.e., without endogenous mobile buffers) by altering the concentration of an exogenous mobile buffer (Neher and Augustine, 1992), in this case EGTA, and monitoring its effect on Ca^2+^ transients. EGTA accelerates the decay of spatially equilibrated Ca^2+^ transients (Atluri and Regehr, 1996; Markram et al., 1998) due to its slow binding rate (10^7^ M^−1^s^−1^; Nägerl et al., 2000). Through competition for free Ca^2+^, we thus expect that the amount of EFB would be inversely proportional to the ability of EGTA to accelerate the decay of the local Ca^2+^ transient. We found no age difference in the decay time course of Ca^2+^ transients recorded at spot locations close to putative Ca^2+^ entry sites (i.e., transients with a rise time <0.5 ms) with either 0.1 mM or 2 mM EGTA-containing pipette solutions (Figures 4A–4C). This result suggests that the concentration and kinetic properties of EFBs do not significantly change between P7 and P14.

To estimate the kinetic properties of EFB, we compared measured Ca^2+^ transients with those predicted from 3D Ca^2+^ reaction-diffusion simulations in which the EFB properties were varied. For these simulations we used experimentally determined parameters, including the microscope point spread function (Figure S3), cytoplasmic diffusion properties of Ca^2+^, and the Ca^2+^ buffering and diffusion properties of Oregon green BAPTA-5N, ATP and EGTA (Table S1). The on and off binding rates of the EFB, and the number of open VGCCs, were free parameters. For a fixed number of open channels at each age (P7 and P14), a low-affinity EFB with fast on and off rate constants (k_on_, 1 × 10^8^ M^−1^s^−1^; k_off_, 1 × 10^4^ s^−1^; K_d_ = 100 μM, Xu et al., 1997), and a buffer capacity of κ = 40 (Helmchen et al., 1997) best matched the experimental measurements obtained with both 0.1 and 2 mM EGTA (Figures 4D and 4E). Moreover, the magnitude of inhibition of the peak Ca^2+^ transient by EGTA in our model (10% for P7 and 9% for P14) matched that of the experimental data (10% for P7 and 11% for P14; Figure S5), suggesting that a low-affinity EFB is present at 4 mM throughout development. When we repeated the simulations with a 50-times higher affinity EFB (k_on_, 5 × 10^8^ M^−1^s^−1^; k_off_, 1 × 10^3^ s^−1^; K_d_ = 2 μM, Meinrenken et al., 2002) (Figure 4D) or lower κ (Figure 4E), the result matched less well to the experimental data. Interestingly, a low-affinity EFB was also required for simulating [Ca^2+^] changes induced by uncaging (Bollmann and Sakmann, 2005). These results show that a fast, low-affinity EFB is present in the calyx of Held and that the kinetic properties of this EFB remain constant during development.

### Developmental Changes in the Sensitivity of Excitatory Postsynaptic Currents to Presynaptic EGTA

**Figure 5:**
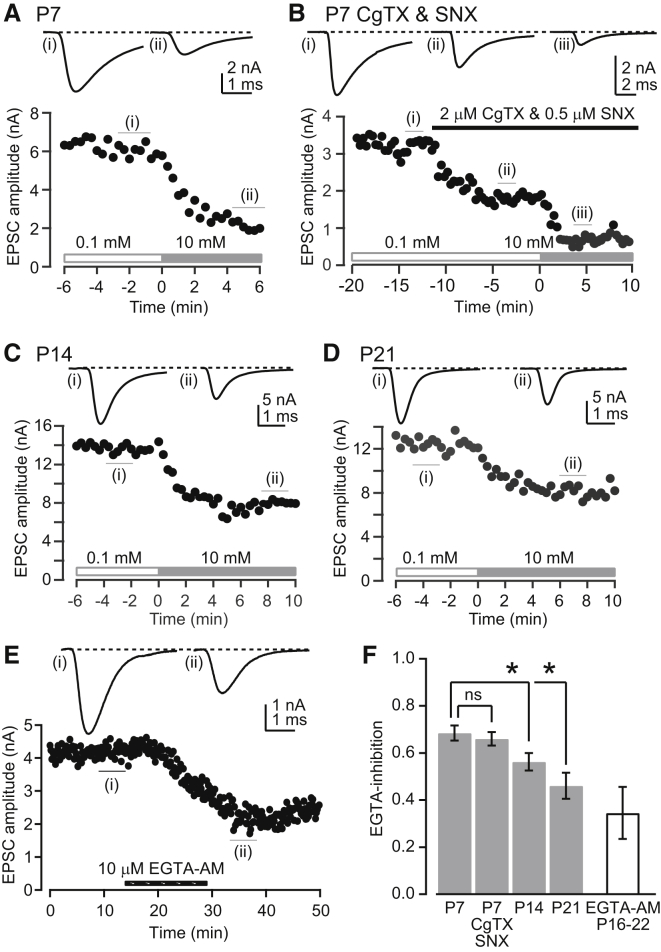
Effects of Intraterminal EGTA Perfusion on the EPSC Amplitude (A) EPSCs evoked by single presynaptic APs (elicited in current clamp) at P7, before (i) and after (ii) presynaptic internal pipette perfusion of 10 mM EGTA. Time 0 is defined by the time at which 10 mM EGTA was infused (horizontal solid gray bar). (B) EPSCs evoked from a P7 calyx before (i) and after (ii) bath application of 2 μM ω-conotoxin and 0.5 μM SNX, and then after presynaptic internal pipette perfusion from 0.1 to 10 mM EGTA (in the presence of VGCC blockers, iii). The reduction in EPSC amplitude due to blockers was 46% ± 5% (n = 5 calyces). (C) Same as (A) but for P14 calyces. (D) Same as (A) but for P21 calyces. (E) Effects of extracellular application of EGTA-AM (10 μM) on EPSCs evoked by extracellular fiber stimulation of an unperturbed P18 calyx. (F) Summary of the EPSC amplitude reduction (EGTA-inhibition; mean ± SEM) due to intraterminal perfusion of 10 mM EGTA (gray bars) at P7–P21 calyces after application of CgTX and SNX (p = 0.86, unpaired t test) or extracellular application of EGTA-AM (open bar). ^∗^p < 0.01; one-way ANOVA followed by Tukey's post hoc.

The sensitivity of excitatory postsynaptic currents (EPSCs) to presynaptic EGTA has been used to assay the VGCC-sensor distance at the calyx of Held (Borst and Sakmann, 1996; Fedchyshyn and Wang, 2005). We revisited the EGTA sensitivity of vesicular release using pipette perfusion, since this method allowed us to change only the [EGTA], thereby removing potentially confounding effects of EGTA-independent changes in synaptic efficacy before and after patch rupture (Fedchyshyn and Wang, 2005). Presynaptic pipette perfusion with the same internal solution (0.1 mM EGTA) as used for whole-cell recording from the calyx had no effect on EPSCs, demonstrating that patch pipette perfusion per se did not affect release properties (Figure S6). After recording EPSCs with the control solution, patch pipette perfusion of a new solution containing 10 mM EGTA reduced the EPSC amplitude within several minutes. At P7, the reduction in EPSC amplitude caused by 10 mM EGTA (EGTA-inhibition) was 69% ± 3% (n = 13, Figure 5A). A similar magnitude of EGTA-inhibition (66% ± 3%, n = 5, Figures 5B and 5F) was observed in the presence of a Ca_v_2.2-specific blocker ω-conotoxin GIVA (CgTX, 2 μM) and a Ca_v_2.3-specific blocker SNX-482 (SNX, 0.5 μM), suggesting the VGCC-sensor distance is similar among VGCC subtypes. Consistent with these results, we observed no significant difference in the spatial distribution of Ca^2+^ entry estimated with confocal line-scans with and without these subtype-specific VGCC blockers (Figure S7), suggesting that Ca_v_2.2 and Ca_v_2.3 have a similar distribution to Ca_v_2.1 before hearing onset.

Prominent inhibitory effects of 10 mM EGTA on the EPSC amplitude were also observed in older calyces, with an EPSC reduction of 56% ± 5% at P14 (n = 13, Figures 5C and 5F) and 46% ± 6% at P21 (n = 10, Figures 5D and 5F). We also tested the effect of membrane-permeable EGTA-AM on the EPSC amplitude in unperturbed P16–P22 calyces, where endogenous mobile Ca^2+^ buffers remained intact. Bath application of EGTA-AM (10 μM) for 15 min reduced the EPSC amplitude by 35% ± 11% (n = 5, Figure 5E), as previously reported in P14–P18 mice (Fedchyshyn and Wang, 2007). Our results with patch pipette perfusion indicate that 10 mM EGTA has a more potent inhibitory effect on the EPSC amplitude at P14–P21 rat calyces (Figure 5F) than reported previously with patch rupture methods (Fedchyshyn and Wang, 2005). Nevertheless, the magnitude of EGTA-inhibition did decrease between P7 and P21 (Fedchyshyn and Wang, 2005).

### Estimating the VGCC-Sensor Distance

**Figure 6:**
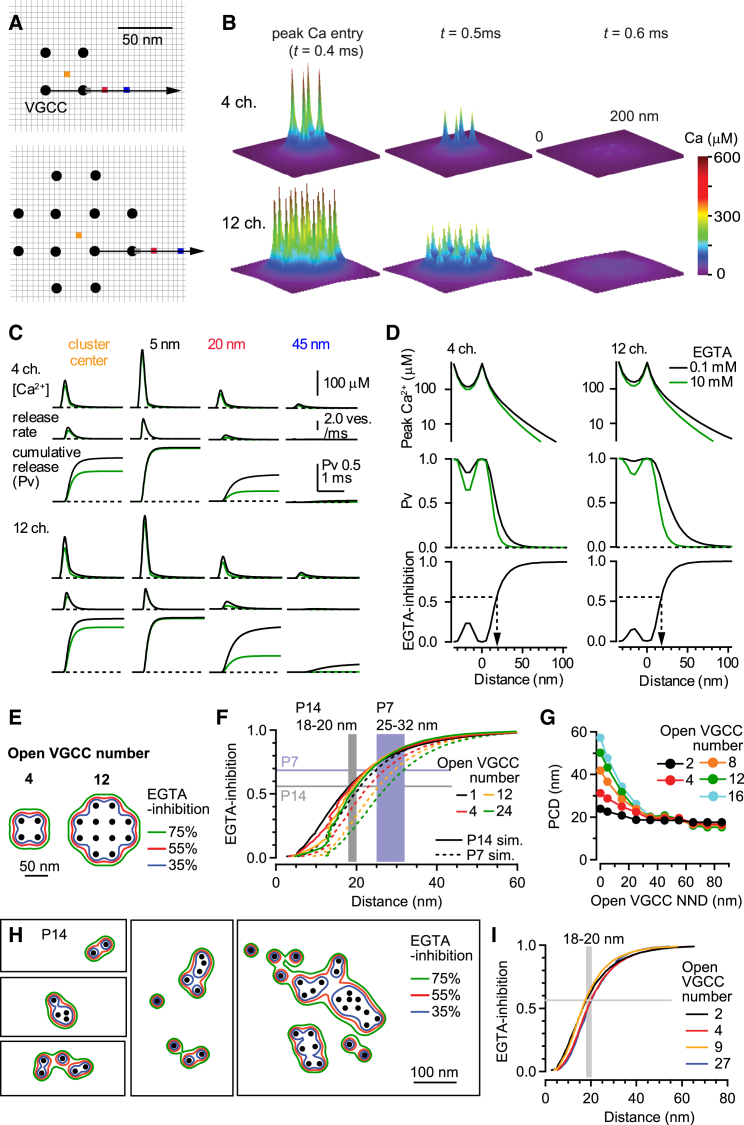
Estimation of the VGCC-Sensor Distance with 3D Reaction-Diffusion Simulations of [Ca^2+^] and Vesicular Release (A) Cartoon of the “grid array” of 4 and 12 open VGCCs (top and bottom; circles) on the terminal membrane for a 3D reaction-diffusion simulation. The NND of the VGCCs was 35 nm, and each voxel was 5 × 5 × 5 nm. Colored voxels correspond to location of simulation traces shown in (C). Arrows indicate the location of line profiles in (D). (B) Spatial distribution of the [Ca^2+^] in single voxels at the terminal membrane generated by the open VGCC clusters in (A), displayed at the time of peak Ca^2+^ entry, 0.1 ms and 0.2 ms after the peak. We set *t* = 0 at the time of a 50% rise time of the presynaptic AP. (C) Time course of [Ca^2+^], vesicular release rate, and cumulative vesicular release probability at colored voxel locations in (A) for control conditions (0.1 mM EGTA, black) and 10 mM EGTA (green). (D) Spatial profile of [Ca^2+^], vesicular release probability (P_v_), and the fractional reduction of P_v_ by 10 mM EGTA (EGTA-inhibition). Dashed line and arrow indicates distance at which 55% EGTA-inhibition was observed. (E) Contour plots for isovalue lines of EGTA-inhibition (35%, 55%, and 75%) around the open VGCC clusters shown in (A). (F) EGTA-inhibition as a function of distance between the vesicular Ca^2+^ sensor and VGCC cluster perimeter. The AP waveform and release sensor parameters were set for either P7 or P14 calyces. Horizontal lines indicate the average experimental values of EGTA-inhibition of 56% for P14 (gray) and 69% for P7 (blue). The vertical shaded regions indicate the range of distances between the sensor location and nearest open VGCC matching experimental EGTA-inhibition for differing number of VGCC per cluster for P14 (solid color lines) and P7 (dashed color lines) simulations. The locations where experimental EGTA-inhibition was observed, called PCDs, were 18–20 nm for P14 and 25–32 nm for P7. (G) NND of open VGCCs versus PCD for different number of open VGCCs. (H) P14 simulations showing EGTA-inhibition isovalues as in (E), but using five representative gold particle clusters (black dots) observed from SDS-FRL EM samples (La1). (I) EGTA-inhibition as a function of distance for VGCC locations corresponding to real gold particle patterns containing different numbers of open VGCCs. The PCD ranged between 18 and 20 nm.

Our measurements of the VGCC distribution (Figures 1 and 2); EFB properties (Figure 4); and the time course of AP-induced Ca^2+^ influx (Figure 3F), along with the previously reported single-channel conductance of Ca_v_2.1 (Sheng et al., 2012), exogenous Ca^2+^ buffer kinetics (Nägerl et al., 2000) and Ca^2+^ sensor models (Kochubey et al., 2009), allowed us to construct an experimentally constrained 3D reaction-diffusion model of Ca^2+^ and vesicular release to estimate the VGCC-sensor distance. We modeled P14 calyces by simulating Ca^2+^ entry induced by an AP_14_ waveform using a simple grid array of either 4 or 12 open VGCCs, where the VGCCs were located 35 nm apart (Figure 6A). These two configurations spanned the range of open VGCCs per cluster, which we estimated from La1 samples (gold particle NND = 37 nm), because the labeling efficiency (0.19) coincides with the channel open probability per AP (0.2; Sheng et al., 2012). In these simulations, we found steep [Ca^2+^] gradients surrounding each open VGCC that dissipated rapidly (<0.5 ms) following termination of Ca^2+^ entry (Figure 6B). The peak amplitude and time course of the [Ca^2+^] at the membrane depended on the distance from the nearest VGCCs (Figures 6C and 6D). In the presence of 0.1 mM EGTA, the peak [Ca^2+^] at 5 nm from the cluster edge was 230 μM for four open VGCCs and 246 μM for twelve open VGCCs, but at 45 nm, this decreased to 16 μM and 22 μM, respectively.

To simulate vesicular release, we drove a five-state Ca^2+^-dependent vesicular release model (Kochubey et al., 2009) using the simulated [Ca^2+^] time courses at various elementary simulation volumes (5 nm cubic voxels) across the entire synaptic face (examples shown in Figure 6C). This approach assumed that the Ca^2+^ sensor for vesicular release was within 5 nm of the membrane, which seems likely given its requisite interaction with the core complex formed by synaptic vesicle fusion proteins (Südhof, 2013). Computing vesicular release probability (P_v_) at different locations with respect to the VGCC cluster, we found P_v_ = 1 within those voxels containing a VGCC, 0.8–1 for voxels within the cluster center, and 0.01 for voxels 55 and 92 nm from the edge of a cluster with 4 and 12 open VGCC, respectively. Increasing the intraterminal [EGTA] from 0.1 to 10 mM inhibited P_v_ by 20% at the cluster center. Only when Ca^2+^ sensors were positioned outside the cluster (19 nm from the closest VGCC; Figure 6D) did EGTA-inhibition match the experimental results at P14 (56%; Figure 5F). Hence, these simulation results predict that synapses with P_v_ > 0.01 have Ca^2+^ sensors for vesicular release located less than 100 nm from the edge of a VGCC cluster (for less than 12 open VGCCs).

Since our SDS-FRL results showed that VGCCs cluster size varied widely, we systematically explored how the number and spatial distribution of open VGCCs within a cluster affect EGTA-inhibition. To do this, we drew contour plots indicating the different isovalue locations of EGTA-inhibition (Figure 6E). The average distance between isovalue positions of 56% EGTA-inhibition (the experimental P14 value) and the nearest open VGCC was insensitive to the number of open VGCCs per cluster, falling between 18 and 20 nm outside the cluster (Figure 6F). Changes in the open VGCC density had little effect on this distance, provided that the NND between open VGCCs was longer than 30 nm (Figure 6G), consistent with the estimate from our SDS-FRL data (Figure 2F). These results suggest that the Ca^2+^ sensor for vesicular release is located within a short distance from the edge of VGCC clusters. We call this topography the perimeter release model and refer to the distance between the closest open VGCC and the Ca^2+^ sensor as the perimeter coupling distance (PCD). Simulations using an AP_7_ waveform and a Ca^2+^ sensor with higher Ca^2+^ affinity for P8–P10 rats (Kochubey et al., 2009) resulted in PCDs = 25–32 nm for the experimentally measured EGTA-inhibition of 69% (Figure 5F), depending on the number of open VGCCs (1-24, Figure 6F). Since real VGCC clusters exhibit irregular shapes (Figures 1 and S2), we repeated reaction-diffusion simulations using our measured La1 immunogold particle distributions for P14 calyces (Figure 6H). Results showed that the PCD for 56% EGTA-inhibition was similar to that of the above grid array models (18–20 nm) and remained insensitive to the number of open VGCCs per cluster (Figure 6I). Thus, the perimeter release model predicts a developmental shortening of the PCD from ∼30 nm at P7 to ∼20 nm at P14.

Our perimeter release model is based on [Ca^2+^] and EPSC measurements from dialyzed terminals, where mobile Ca^2+^ buffers are washed out and partially replaced by 0.1 mM EGTA. Because expression of the mobile buffer calretinin increases with development at calyces of Held (Felmy and Schneggenburger, 2004), we evaluated its impact on P_v_ and PCD. Simulations including 0.5 mM or 1.2 mM calretinin reduced P_v_ by 12% and 24%, respectively (Figure S8A), but had no effect on the PCD estimate (20 nm at P14; data not shown). Thus, our model is relatively insensitive to the presence of calretinin even at high concentrations.

The single-channel current amplitude used here for AP_14_ waveform (0.35 pA) is based on single-channel conductance (3.3 pS in 2 mM [Ca^2+^]) measured for Ca_v_2.1 at the calyx of Held (Sheng et al., 2012). This channel conductance is similar to that of the Ca_v_2.2 (2.7 pS) at an autonomic preganglionic synapse (Weber et al., 2010). However, because the single-channel current amplitude can influence the coupling distance (Weber et al., 2010), we examined the model sensitivity to variations in the single-channel current. For single-channel currents greater than or equal to 0.2 pA, the 56% EGTA-inhibition would be outside the cluster (Figure S8B), supporting the perimeter release model.

### Estimating the Vesicular Release Probability in the Vicinity of a VGCC Cluster

**Figure 7:**
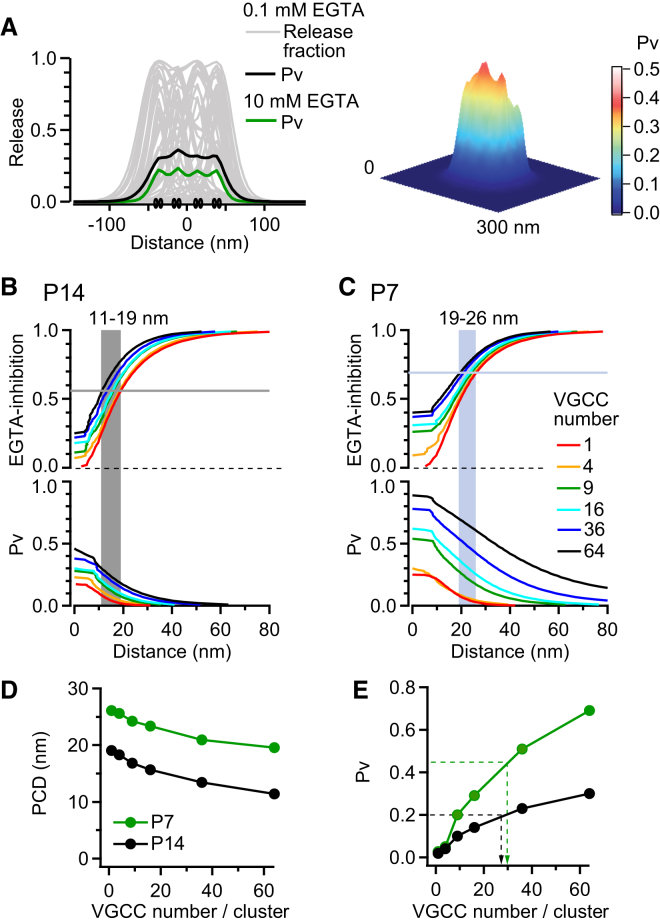
Estimation of the PCD and Vesicular Release Probability in a Model with Randomly Generated Patterns of VGCC Openings (A) Left: Spatial line profiles (50 trials, gray lines) showing the fraction of sensors in the release state (release fraction) for voxels at the membrane. Release fraction of each trial was simulated from a randomly generated open channel pattern with open probability of 0.175 and a grid arrangement of 16 VGCCs with NND = 20 nm and [EGTA] = 0.1 mM. Black trace is the average across all trials and thus represents P_v_. Green trace is the trial average for [EGTA] = 10 mM. Pairs of solid black ovals denote the VGCC locations. Right: 2D plot of the average P_v_ (0.1 mM EGTA). (B) EGTA-inhibition and P_v_ predicted for different sensor locations and numbers of channels using a P14 model (AP_14_ and P14 Ca^2+^ sensor model). Horizontal line indicates the mean EGTA-inhibition of P_v_ observed in experiments. Gray region indicates PCD range for different number of VGCCs per cluster. (C) Same as (B) but for a P7 simulation (AP_7_ and P7 Ca^2+^ sensor). (D) PCD plotted against the total number of VGCCs within each cluster. (E) P_v_ for AP_7_ (green) or AP_14_ (black) plotted against the numbers of VGCCs within each cluster. P7 and P14 Ca^2+^ sensors were used, respectively. Arrows indicate predicted total number of VGCCs per cluster: 29 and 26 for P7 and P14, respectively.

We next examined whether trial-to-trial variability in the pattern of open VGCCs altered our estimate of the PCD. To do this, we generated 50 different patterns of open VGCCs using the VGCC open probability during an AP, calculated from single-channel recordings (Sheng et al., 2012) (see Supplemental Experimental Procedures). The fraction of sensors in the release state (release fraction) was calculated at each surface-membrane voxel, for each pattern of open VGCCs (Figure 7A, gray traces). As for the deterministic simulations (Figure 6), the release fraction was equal to 1 within voxels where a VGCC opened and then dropped steeply within the cluster at locations where channels did not open (Figure 7A). P_v_ was calculated by averaging the release fraction across trials. At the center of the cluster composed of 16 VGCCs the peak P_v_ was 0.45. P_v_ then decreased with distance outside the cluster (Figure 7B, bottom). These simulations predicted the experimentally observed level of EGTA-inhibition at shorter PCDs than those with fixed VGCC distributions, ranging from 11 to 19 nm for P14 and 19 to 26 nm for P7 (Figures 7B and 7C). The PCD was weakly dependent on the number of VGCCs per cluster (Figure 7D). In contrast, P_v_ was strongly dependent on the number of VGCCs per cluster (Figure 7E). Moreover, these simulations predict that an average of 20–30 VGCCs per cluster underlie the observed P_v_ at P7 and P14. Thus, our perimeter release model predicts that the number of VGCCs per cluster has a minor effect on the PCD but is an important determinant of P_v_.

### Contributions of AP Duration and PCD to Changes in Synaptic Delay and Release Duration during Development

**Figure 8:**
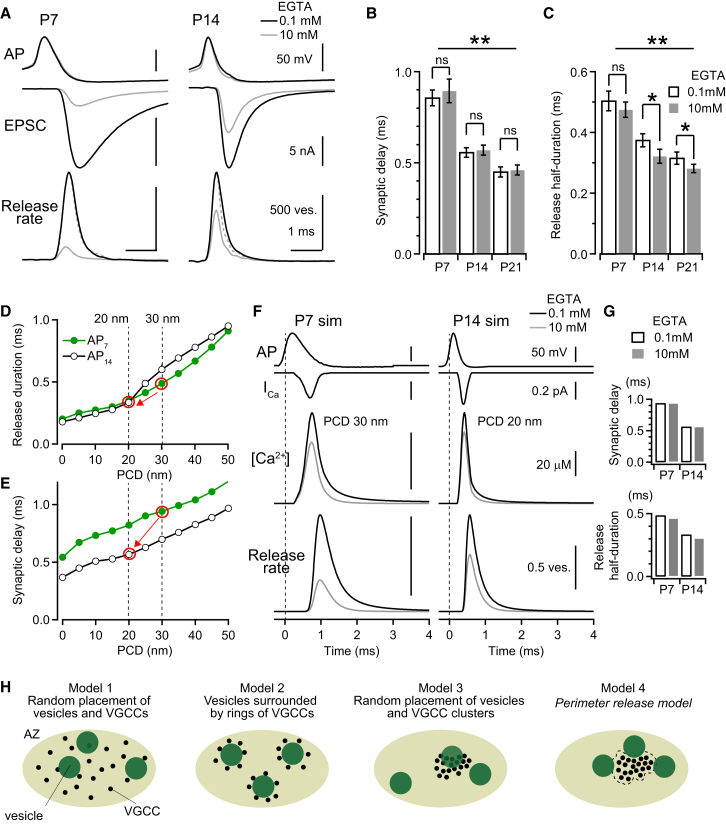
Developmental Changes in Vesicular Release Time Course Are Predicted by Perimeter Release Model (A) Representative examples of experimentally measured presynaptic APs, EPSCs, and vesicular release rates before (black) and after (gray) presynaptic perfusion of 10 mM EGTA in P7 and P14 calyces. Dashed lines indicate peak scaked release rate in 10 mM EGTA. (B) Mean synaptic delay (from the 50% rise time of the APs to the 20% rise time of the EPSCs, ± SEM) in the presence of 0.1 mM (open bars) or 10 mM EGTA (filled bars) in the presynaptic pipette solution (n = 10 calyces for P7, n = 8 for P14 and P21). (C) Release half duration (the width at half maximal of the release rate, ± SEM) in the presence of 0.1 mM and 10 mM EGTA, estimated by deconvolution at P7, P14, and P21 (^∗∗^p < 0.01, one-way ANOVA). Internal perfusion of 10 mM EGTA reduced the release duration by ∼15% in P14 and P21 (^∗∗^p < 0.05, paired t test), but not in P7 calyces. (D) Dependence of release duration on the PCD for AP_7_ (green) and AP_14_ (black) waveforms, simulated with the perimeter release model. Red circles indicate values predicted by experimental results, and an arrow indicates the direction of developmental change. (E) Same as (D) but for synaptic delay. (F) Temporally aligned simulated traces of the [Ca^2+^] and vesicular release rate for [EGTA] = 0.1 mM (black) and 10 mM (gray). For P7 and P14 simulations, the timing and duration of Ca^2+^ entry, number of open VGCCs, Ca^2+^ sensor affinity, and PCD for were adjusted specifically for each age. (G) The simulated effect of 10 mM EGTA on synaptic delay and vesicular release duration for the perimeter release model. (H) Cartoons showing possible AZ topographies for VGCCs and synaptic vesicles released by a single AP. Model 1, random placement of vesicles and VGCCs within AZ. Model 2, vesicles surrounded by rings of VGCCs. Model 3, random placement of vesicles and VGCC clusters, including within vesicle clusters. Model 4, perimeter release model, where releasable synaptic vesicles are positioned at the perimeter of a VGCC cluster. Whether there are more than one releasable vesicle is only speculative.

During the period of hearing acquisition, the synaptic delay between the presynaptic AP and the EPSC becomes shorter, and the time course of vesicular release becomes faster at the calyx of Held (Taschenberger and von Gersdorff, 2000; Taschenberger et al., 2005). Numerical simulations suggest that these developmental changes might be mediated by alterations in the VGCC-sensor distance (Bucurenciu et al., 2008), but other findings argue against this hypothesis (Meinrenken et al., 2002). We re-examined this issue by measuring the synaptic delay and time course of vesicular release during EGTA dialysis. Our results show that internal perfusion of 10 mM EGTA had no effect on the synaptic delay at any age investigated, whereas the synaptic delay became shorter between P7 to P21 (Figures 8A and 8B). Moreover, internal perfusion of 10 mM EGTA produced only a modest reduction in the time course of vesicular release at P14 and P21 (∼10%), and no change at P7, whereas the half duration of release was reduced by 29% from P7 to P14 (Figure 8C), as previously reported (Taschenberger et al., 2005).

We next examined whether our perimeter release model predicted the observed changes in synaptic delay and time course of vesicular release at different postnatal ages. Simulations for postnatal day 7 (P7) and P14 were performed using AP_7_ and AP_14_ waveforms and previously reported sensitivities of the Ca^2+^ sensors for each age (Kochubey et al., 2009). We used a fixed pattern cluster containing six open VGCCs for P7 and four for P14, corresponding to the channel open probability per AP multiplied by the total number of channels per cluster estimated from EM (see Discussion for more details). Simulation results predicted that the vesicular release duration and synaptic delay are steeply influenced by PCD (Figure 8D). Surprisingly, the different AP waveforms between P7 and P14 had a relatively minor effect on the vesicular release duration, whereas they had a marked influence on the synaptic delay (Figure 8E). In contrast, shortening of PCD from 30 nm (for P7) to 20 nm (for P14, see Figure 6) predicted a 31% decrease in the vesicular release half duration (Figure 8D, red arrow) comparable to mean experimental value (28%, Figure 8C). The shortening of synaptic delay (35%, Figure 8B) can be achieved through both changes in AP duration and PCD in simulation, which predicts 40% reduction (Figure 8E, red arrow). The overall release time course for P7 and P14 simulations (in 0.1 and 10 mM EGTA) was comparable to the experimental findings (compare Figures 8F and 8A). Moreover, increasing [EGTA] from 0.1 to 10 mM in the simulations had little effect on the synaptic delay and only a small reduction in the vesicular release half duration (Figure 8G), similar to our experimental findings (Figures 8B and 8C).

Neither the number of open VGCCs (Figure S8C) nor the sensitivity of the Ca^2+^ sensor (Figure S8D) affected the synaptic delay or release time course. A fast, low-affinity EFB resulted in a larger change in the release time course for increasing VGCC-sensor distance (Figures S8E and S8F), unlike model predictions using a high-affinity EFB (Meinrenken et al., 2002). Thus, a low-affinity EFB is critical for predicting the developmental speeding of the release time course. These simulations suggest that developmental shortening of the PCD from 30 to 20 nm is the main determinant for the developmental acceleration of the vesicular release time course (Figure 8D). In contrast, shortening of the PCD accounted for the developmental reduction in synaptic delay only partially (Figure 8E), with the remaining changes caused by the shortening of the AP duration. In simulations using stochastic patterns of open VGCC (Figure 7), the shortening of PCD from 25 to 15 nm also reproduced the developmental changes synaptic delay and release time course (Figure S8G). Thus, the shortening of PCD contributes to the developmental acquisition of synaptic precision that is critical for sound localization at the calyx of Held (Oertel, 1999).

## Discussion

We investigated the mechanisms that underlie the speed and precision of vesicular release at the rat calyx of Held, a central excitatory synapse that has been particularly well characterized across different developmental stages. We measured the properties of two key presynaptic parameters that have not previously been quantified: the distribution of VGCCs and the binding kinetics of endogenous fixed Ca^2+^ buffers. Moreover, we re-examined the EGTA sensitivity of neurotransmitter release under more controlled conditions than has been previously achieved. These results were then combined with other known experimental parameters to constrain 3D reaction-diffusion simulations to examine how different topographical arrangements of VGCCs and vesicles affect neurotransmitter release and to estimate the VGCC-sensor distance at different developmental stages. Our experimental results and simulations suggest a model in which release-ready vesicles are located within tens of nanometers of the outer perimeter of the VGCC clusters. This perimeter release model predicts the properties of vesicular release at the calyx of Held at different developmental stages. Our model provides a new framework for understanding mechanisms that determine high-fidelity transmission at central synapses.

### The Number of VGCCs that Contribute to the Release of a Vesicle

Whether vesicular release requires opening of one (Stanley, 1993) or multiple VGCCs (Borst and Sakmann, 1996; Fedchyshyn and Wang, 2005; Bucurenciu et al., 2010; Nadkarni et al., 2010; Scimemi and Diamond, 2012; Sheng et al., 2012) is controversial. The SDS-FRL results reported here show that Ca_v_2.1 channels exist primarily in clusters. Our experimentally constrained model indicates that a single VGCC close to a vesicle can induce vesicle fusion, albeit with a low P_v_ (0.02 to 0.03, depending on VGCC open probability). This finding could account for spontaneous EPSCs (Ermolyuk et al., 2013) but not AP-evoked release probability estimated for the whole calyx (0.45 at P7 and 0.19 at P14; Koike-Tani et al., 2008). Our results therefore suggest that at the majority of release sites multiple VGCCs contribute to the release of each vesicle following an AP at the calyx of Held.

In order to estimate the total number of VGCCs per cluster from our immunogold particle distributions, we first calculated the labeling efficiency using whole-terminal Ca^2+^ current measurements (see Supplemental Experimental Procedures). To minimize errors, we further analyzed the higher efficiency samples (La2). At P14, the number of Ca_v_2.1 channels per cluster was 18 (=11.1/0.62) on average and varied between 3 and 73 across clusters. At P7 we estimate that there are 10 Ca_v_2.1 channels per cluster, but because Ca_v_2.1 channels comprise 53% of the VGCCs at P7 (Figure S7), the total number of VGCCs on average is 19. We used a 100 nm radius circle for cluster analysis because it optimally detected real clusters (Figure S1F). We cannot rule out, however, the possibility that some clusters are composed of smaller, closely spaced subclusters (e.g., Figure S2C1). Using numerical simulations of release from stochastic open channel patterns based on the open probability of 0.25 at P7 (Sheng et al., 2012) and 0.175 at P14 (see calculation in Supplemental Experimental Procedures), we predict that 29 (at P7) and 26 (at P14) VGCCs are required (Figure 7E) to reproduce the mean P_v_ of whole terminal (Koike-Tani et al., 2008). The close match between the anatomical and functional estimates of the number of VGCCs per cluster indicates that physiological vesicular release at the calyx of Held is driven by channel clusters with an average of 20–30 VGCCs.

### A Unifying Model for Understanding AP-Evoked Vesicular Release at Central Synapses

Our experiments and simulations suggest a topographical arrangement where most releasable vesicles (in response to a single AP) are located 15–30 nm from the outer perimeter of VGCC clusters (Figure 8H, model 4). However, several other VGCC-sensor topographies have been proposed at mammalian central synapses, including random distributions of both VGCCs and release-ready synaptic vesicles within the AZ (model 1), synaptic vesicles surrounded by a ring of VGCCs (model 2), clusters of VGCCs, and a random distribution of synaptic vesicles within the AZ (model 3, Meinrenken et al., 2002; Schneggenburger and Neher, 2005; Wang et al., 2009; Scimemi and Diamond, 2012; Ermolyuk et al., 2013). SDS-FRL at the calyx of Held indicates that Ca_v_2.1 channels are clustered at high densities on the synaptic face in areas smaller than AZs (Figures 1 and 2). These observations are compatible with both model 3 and model 4. However, simulations of model 3 (Figure S8H) did not replicate our experimental levels of EGTA inhibition of vesicular release (Figure 5), unless a high number of VGGCs were placed within each cluster (>50 for P14). Because we estimate that 90% of the VGCC clusters contain less than 50 VGCCs (Figure 2D, after labeling efficiency correction), our experimental findings are not consistent with model 3. Moreover, EGTA inhibition and P_v_ were both predicted by the perimeter release model 4 when the Ca^2+^ sensor was positioned within tens of nanometers from the edge of the VGCC cluster. Although we cannot rule out that vesicles with negligible P_v_ are located further away, our perimeter release model is consistent with the majority of experimental results on AP-evoked vesicular release at the calyx of Held.

Since Ca_v_2.1 channels also form clusters within AZs at hippocampal (Holderith et al., 2012) and cerebellar synapses (Indriati et al., 2013), the latter of which has been suggested to have a coupling distance of ∼20 nm (Schmidt et al., 2013), our perimeter release model may also be applicable to bouton-type synapses. A recent study at hippocampal mossy-fiber synapses suggests that vesicular release is driven by loose VGCC-sensor coupling (65 nm; see Figure 2C in Vyleta and Jonas, 2014). Using their 90% reduction of EPSCs by 10 mM EGTA, our perimeter release model predicts a similar coupling distance (four open VGCCs, PCD = 40 nm, or 55 nm from center; see Figure 6D) and a slower vesicular release time course for the mossy-fiber terminal (see Figure 8D). However, it remains to be determined if VGCCs also cluster at that synapse. Finally, a similar model has been proposed at invertebrate synapses, where fast phasic release requires a coupling distance of <20 nm (Pan and Zucker, 2009). These findings suggests that short coupling distances (<100 nm) are a general requirement for fast AP-evoked vesicular release. Thus, within the framework of the perimeter release model it is possible to understand many aspects of vesicular release across a wide range of synapse types.

### Molecular Implication of the Perimeter Release Model

What mechanism might hold vesicles near the perimeter of VGCC clusters? The low-affinity vesicular Ca^2+^ sensor synaptotagmin can directly interact with VGCCs at their intracellular loops at high [Ca^2+^] (Chapman and Davis, 1998; Watanabe et al., 2010). Overexpression of mutated synaptotagmin1 in the calyx of Held reduces the pool size of release-ready vesicles, increases synaptic delay, and decreases the vesicular release rate, suggesting a role of synaptotagmin in positional vesicle priming (Young and Neher, 2009). The vesicular Rab (3/27)-binding protein RIM1a binds to VGCCs via their β subunits (Kiyonaka et al., 2007). Genetic deletion of RIM1 and RIM2 reduces the VGCC density and the number of docked vesicles at calyces of Held (Han et al., 2011). Thus, synaptotagmins and RIMs may also tether vesicles close to VGCCs. Munc13-1 has also been suggested to mediate the tethering of synaptic vesicles to VGCCs at the calyx of Held (Chen et al., 2013). In contrast, Septin 5 has been proposed to prevent vesicles from approaching VGCCs too closely, and its developmental downregulation is postulated to underlie developmental shorting of the VGCC-sensor coupling distance (Yang et al., 2010). Thus, a number of AZ proteins could be involved in tethering synaptic vesicles close to the perimeter of VGCC clusters (Hallermann and Silver, 2013) and orchestrate the developmental shortening of the VGCC-sensor distance at the perimeter of VGCC clusters.

### Functional Implications of the Perimeter Release Model

Restricting vesicular release to the perimeter of VGCC clusters has several important advantages. First, this topography will minimize disruption of VGCC clusters during vesicular fusion with the plasma membrane (Mercer et al., 2011). Second, the number of VGCC in a cluster can regulate P_v_ without altering the time course of vesicular release (Figures 7B and 7C). Moreover, the heterogeneity of P_v_ across AZs (Sakaba and Neher, 2001) could be explained by different numbers of VGCCs per cluster, rather than by variable VGCC-sensor distances (Meinrenken et al., 2002). Third, this topography could potentially allow multiple vesicles to be primed at an AZ, enabling multivesicular release with high precision at the calyx of Held (Taschenberger et al., 2002; Budisantoso et al., 2013). Hence, localizing vesicular release close to the perimeter of VGCC clusters ensures the synaptic precision is maintained over a wide range of synaptic strength.

## Experimental Procedures

All experiments were conducted in accordance with the guidelines of Doshisha University, Institut Pasteur, the National Institute for Physiological Sciences, and Institute of Science and Technology Austria.

### Electron Microscopy and Analysis of SDS-Digested Freeze-Fracture Replica Labeling

P7–P21 rats and mice were perfused with fixative, and brain slices were frozen, fractured, and replicated as described previously (Indriati et al., 2013). Replicas were incubated with an antibody against Ca_v_2.1 subunit of Ca^2+^ channel (8.1 μg/ml; Miyazaki et al., 2012) overnight followed by incubation with 5 nm gold-conjugated secondary antibody at 15°C. Identification of 5 nm particles on the replicas was based on their size, round shape, and electron density. Platinum coating of intramembrane particles often produced dark shadows that were easily distinguished from gold particles and thus excluded from analysis. Labeling efficiency of Ca_v_2.1 was estimated by comparing the overall density of the Ca_v_2.1 immunogold particles with the whole-terminal I_Ca_ at P14. The labeling efficiencies of La1 and La2 were 19% and 62%, respectively. We assumed the same labeling efficiency across ages.

### Slice Electrophysiology and Ca^2+^ Imaging

Whole-cell patch-clamp recordings were made from calyces of Held and MNTB neurons of acute brainstem slices prepared from P7–P22 Wistar rats. Presynaptic AP, I_Ca_, and EPSCs were recorded with a Multiclamp-700 (A or B) amplifier (Molecular Devices). Presynaptic internal solution exchange was performed using pipette perfusion, as described previously (Takahashi et al., 2012). Confocal laser scanning and spot detection of fluorescence was performed using an Ultima scanning head (Prairie Technologies) mounted on an Olympus BX61W1 microscope and equipped with a 60×(1.1 NA) water immersion objective. We monitored intracellular [Ca^2+^] changes with Oregon green BAPTA-5N added to presynaptic pipette solution. All experiments were performed at room temperature (22°C–24°C). Data analysis was performed with IgorPro 6.3 (WaveMetrics) using NeuroMatic. Deconvolution of EPSCs was performed as describe previously (Sakaba and Neher, 2001; Taschenberger et al., 2005). All values in the text and figures are given as means ± SEM unless otherwise indicated.

### Numerical Simulations Ca^2+^ Reaction-Diffusion and Vesicular Release

Ca^2+^ diffusion and binding with Oregon green BAPTA-5N and buffers in the vicinity of VGCC clusters was simulated using a finite-difference method (DiGregorio et al., 1999; DiGregorio et al., 2007) in order to predict spot-detected Ca^2+^ transients. Nanoscale simulations were performed without the Ca^2+^ indicator and then used to drive a five-state Ca^2+^-dependent release model (Kochubey et al., 2009). All simulation parameters are listed in Tables S1 and S2.

## Supplemental Information

Document S1. Figures S1–S8, Tables S1 and S2, and Supplemental Experimental Procedures

Document S2. Article plus Supplemental Information
