# Supplementary material for: Nanoscale Distribution of Presynaptic Ca2+ Channels and Its Impact on Vesicular Release during Development
Source: Neuron. 2015 Jan 7;85(1):145–58. doi: 10.1016/j.neuron.2014.11.019 (PMC4305191; doi:10.1016/j.neuron.2014.11.019)
Supplement: Document S1. Figures S1–S8, Tables S1 and S2, and Supplemental Experimental Procedures [file mmc1.pdf]

Neuron, Volume *85*

Supplemental Information

Nanoscale Distribution of Presynaptic Ca^{2+} channels and Its Impact on Vesicular Release during Development

Yukihiro Nakamura, Harumi Harada, Naomi Kamasawa, Ko Matsui, Jason S. Rothman, Ryuichi Shigemoto, R. Angus Silver, David A. DiGregorio, and Tomoyuki Takahashi

Inventory of supplemental information

Supplemental Data

Figure S1 (related to Figures 1 and 2). Comparison of $Ca_v2.1$ gold particle labeling in control and in $Ca_v2.1$ knockout mice, and determination of criterion for cluster analysis
Figure S2 (related to Figures 1 and 2). Application of cluster criterion to SDS-FRL images, and co-staining of $Ca_v2.1$ and RIM to identify active zones
Figure S3 (related to Figure 3). Estimation of confocal spot point spread function
Figure S4 (related to Figure 3). Spatial dependence of measured and simulated AP-evoked Ca^{2+} transients
Figure S5 (related to Figure 3). Simulation of developmental changes in AP-evoked Ca^{2+} transients
Figure S6 (related to Figure 5). Pipette perfusion control experiments
Figure S7 (related to Figure 5). Spatial distribution of presynaptic Ca^{2+} influx during development
Figure S8 (related to Figures 6 and 8). Influence of model parameters on vesicular release simulations
Table S1 (related to Figure 4) Model parameters for simulations of fluorescence Ca^{2+} transients
Table S2 (related to Figures 6, 7 and 8) Model parameters for simulations of Ca^{2+} diffusion and vesicular release

Supplemental Experimental Procedures

- Animals
- Electron microscopy and analysis of SDS-digested freeze-fracture replica labeling
  - SDS-FRL
  - Immunoparticle distribution analysis
  - Estimation of labeling efficiency
- Slice electrophysiology and Ca^{2+} imaging
  - Brainstem slice preparation
  - Electrophysiological recordings and data analysis
  - Measurements and analysis of Ca^{2+} transients
- Numerical simulations of Ca^{2+} reaction-diffusion and vesicular release
  - Simulations of fluorescence Ca^{2+} transients
  - Simulations of nanoscale [Ca^{2+}] and vesicular release

Supplemental References

SUPPLEMENTAL DATA

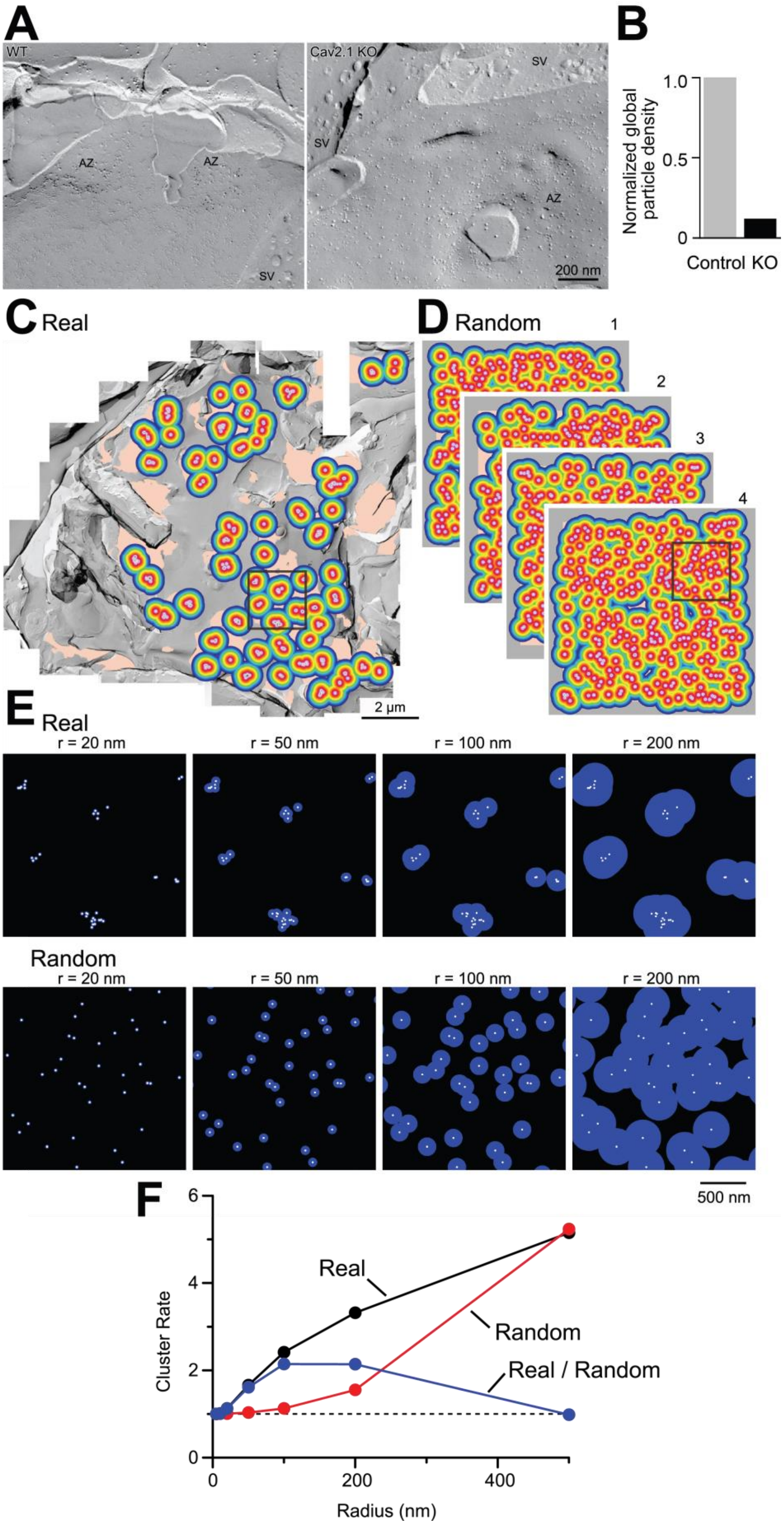

Figure S1. Comparison of $Ca_v2.1$ Gold Particle Labeling in Control and in $Ca_v2.1$ Knockout Mice, and Determination of Criterion for Cluster Analysis

(A) SDS-FRL immunogold labeling of $Ca_v2.1$ at the presynaptic P-face of the calyx of Held of a wild-type mouse (WT, left panel, P13). Presence of synaptic vesicles (SVs) in the cross-fracture through the cytosol confirms that this is the presynaptic P-face. As in rats, immunogold particles were mostly found as clusters. In a $Ca_v2.1$ knockout (KO) mouse (right panel, P13), the clustered pattern of immunogold labeling was not observed in the presynaptic P-face, not even near concaved surface with dimples, which indicates putative AZs.

(B) Global immunogold particle density of wild-type and $Ca_v2.1$ KO mice, both normalized to wild-type levels. The mean density for $Ca_v2.1$ KO mice was no more than 10% of wild-type (not shown), similar to the background level and confirming the specificity of the antibody (n = 2 murine calyces for both).

(C) SDS-FRL immunogold labeling of $Ca_v2.1$ at the presynaptic P-face (pink area) of a calyx of Held from a P21 rat. Distance from each pixel to the nearest neighbor immunogold particle is expressed in a pseudocolor map. The nearest distance from the pixels on the blue line to the immunogold particle is 480 nm.

(D) The same number of immunogold particles was distributed randomly in a square area with the same size as the sum of the presynaptic P-face area exposed in (C). 100 of such random distributions were made and distance maps were created for all.

(E) Comparison of real immunogold particle clusters with randomly distributed particles. Enlarged 2 × 2 µm areas in (C) and (D) are shown. Each particle location is shown with a white dot. Blue circles with the specified radius (upper panel) were drawn around each particle. By systematically varying the radius of these circles, we probed the criteria that defined reasonable clusters. When the circle radius was 50 nm, circles showed some overlap in the real distribution, but almost no overlap in the random distribution. When the circle radius was 200 nm, circles nearly filled the entire field in the random distribution, but a substantial fraction of the field remained unoccupied by the circles in the real distribution.

(F) To search for the optimal circle radius for defining a cluster, we calculated the 'cluster rate' between real and random distributions. The area that the circles occupy (S_T) was divided by the area of a circle around a single particle (S_C). The total number of immunoparticles (N_P) was divided by S_T/ S_C and we defined this value (N_P S_C / S_T) as the "cluster rate". If there is no overlap between circles, the cluster rate is 1. The cluster rate increases, however, when circles begin to overlap. With a large enough radius, the cluster rate approaches the total number of immunoparticles. As we increased the circle radius, the 'cluster rate' increased in both real and random distributions. The former slope was steeper than the latter below 100 nm, but above 200 nm 'cluster rate' in the random distribution increased more steeply. The maximal separation between random and real distributions was found at 100 nm, where the 'cluster rate' for the real distribution (black) divided by that for the random distribution (red) showed the largest value (blue).

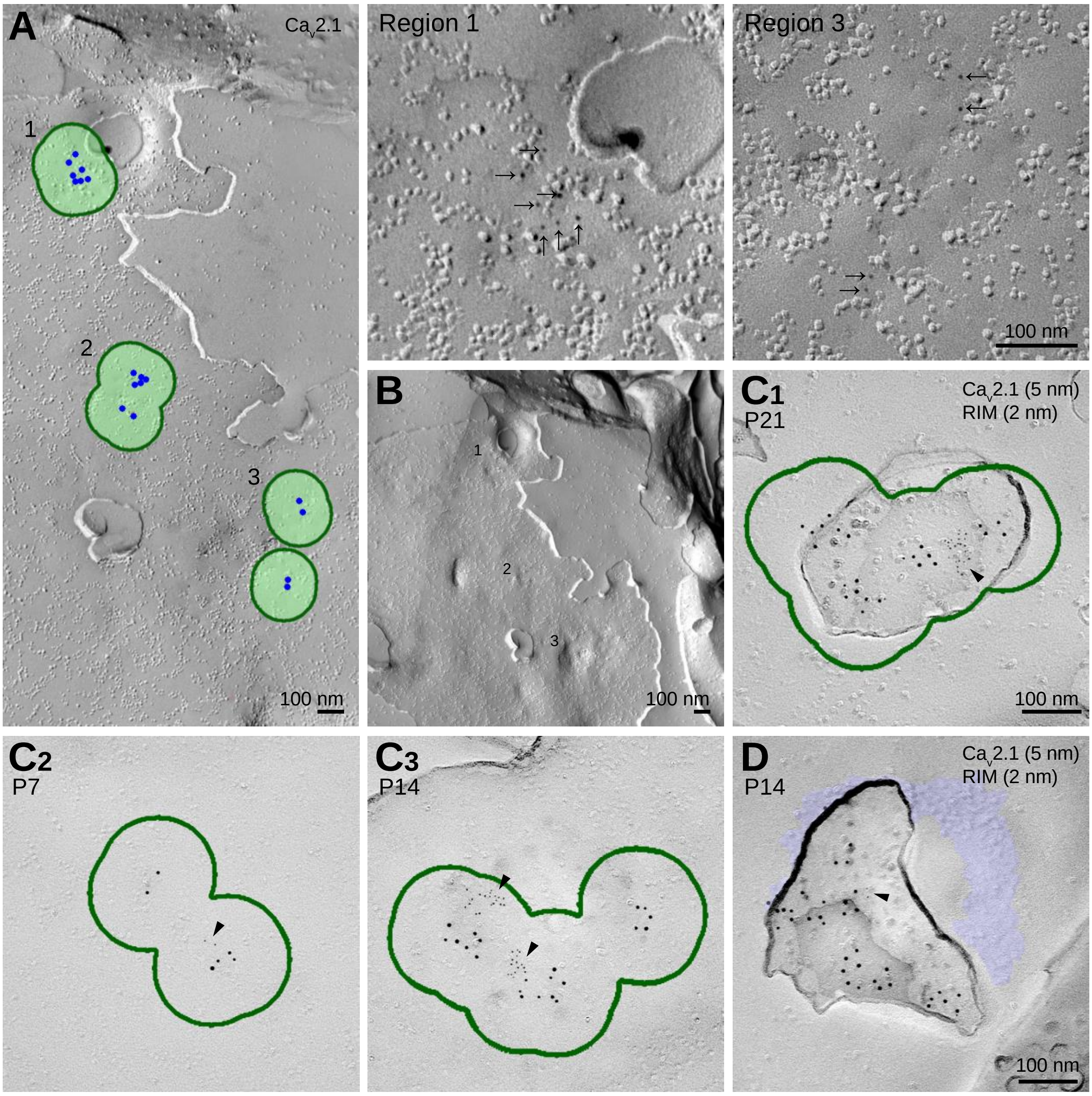

Figure S2. Application of Cluster Criterion to SDS-FRL Images, and Co-staining of $Ca_v2.1$ and RIM to Identify Active Zones

(A) *Left*: The replica image shows the same region as Figure 1A1. $Ca_v2.1$ particles are labeled with blue dots and cluster area is labeled with green. *Right*: Regions 1 and 3 are shown at a higher magnification. Region 2 is shown in Figure 1A2 and 1A3. Arrows indicate the location of each immunogold particle. Gold particles in region 3 are separated into two clusters according to our definition of cluster with a 100 nm radius. Note that NND of the two clusters is short (250 nm) in this case. Such a short NND observed in La1 was not seen in La2 with higher labeling efficiency. This is presumably because the lower channel labeling efficiency in La1 resulted in misclassifications of some large clusters as two closely space clusters.

(B) The same region as (A) viewed with a higher tilt (40 degrees) shows clusters 1-3 on concaved surface. $Ca_v2.1$ particle clusters are often found near such a concaved surface with dimples.

(C) Representative images of double immunogold labeling of $Ca_v2.1$ and RIM. Immunogold particles for RIM (2 nm, marked with arrowheads) were detected close to $Ca_v2.1$ particles (5 nm) in P21 (C1), P7

(C2), and P14 (C3, D) replicas, falling within the defined area of a cluster (green line), supporting that $Ca_v2.1$ clusters are located in AZs at all ages examined.

(D) One example of replica image showing a transition of the postsynaptic exoplasmic face to the presynaptic P-face within a synapse. An intra-membrane particle cluster indicating excitatory postsynaptic site on the exoplasmic face is labeled with light purple. Immunogold particles for $Ca_v2.1$ were observed in a small "window" showing the presynaptic P-face continuous to the postsynaptic site. Scale bars are 100 nm.

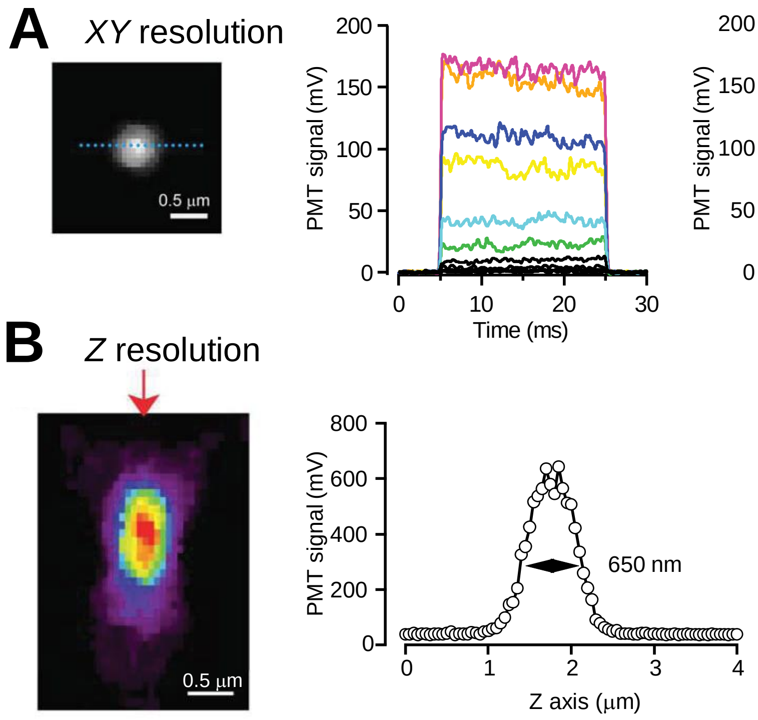

Figure S3. Estimation of Confocal Spot Point Spread Function

(A) *Left*: Confocal laser scanning image of a 100 nm green fluorescence bead with light blue dots indicating spot locations for the point scan. *Center*: Fluorescence traces recorded when the confocal spot is placed at locations along a line crossing the fluorescence bead in 80 nm steps. *Right*: Fluorescence intensity of each trace versus spot location from a representative scan. The fluorescence intensity during the 20 ms laser pulse was averaged. Red trace is a Gaussian fit from which the full width at half maximum was determined. The average full width at half maximum from 5 different beads was 220 ± 3 nm.

(B) *Left*: *x-z* plot of the scanning confocal point spread function created by imaging a 100 nm fluorescent bead. Focal planes were adjusted using a piezoelectric drive in 50 nm steps. Red arrow indicates the location of the intensity line profile along the *z*-axis. *Right*: *z*-axis intensity line profile (circles; average of 9 neighboring pixels) and its Gaussian fit (solid line). The average full width at half maximum of Gaussian fits *x-z* plots from image stacks of 6 different fluorescence beads was 650 ± 12 nm.

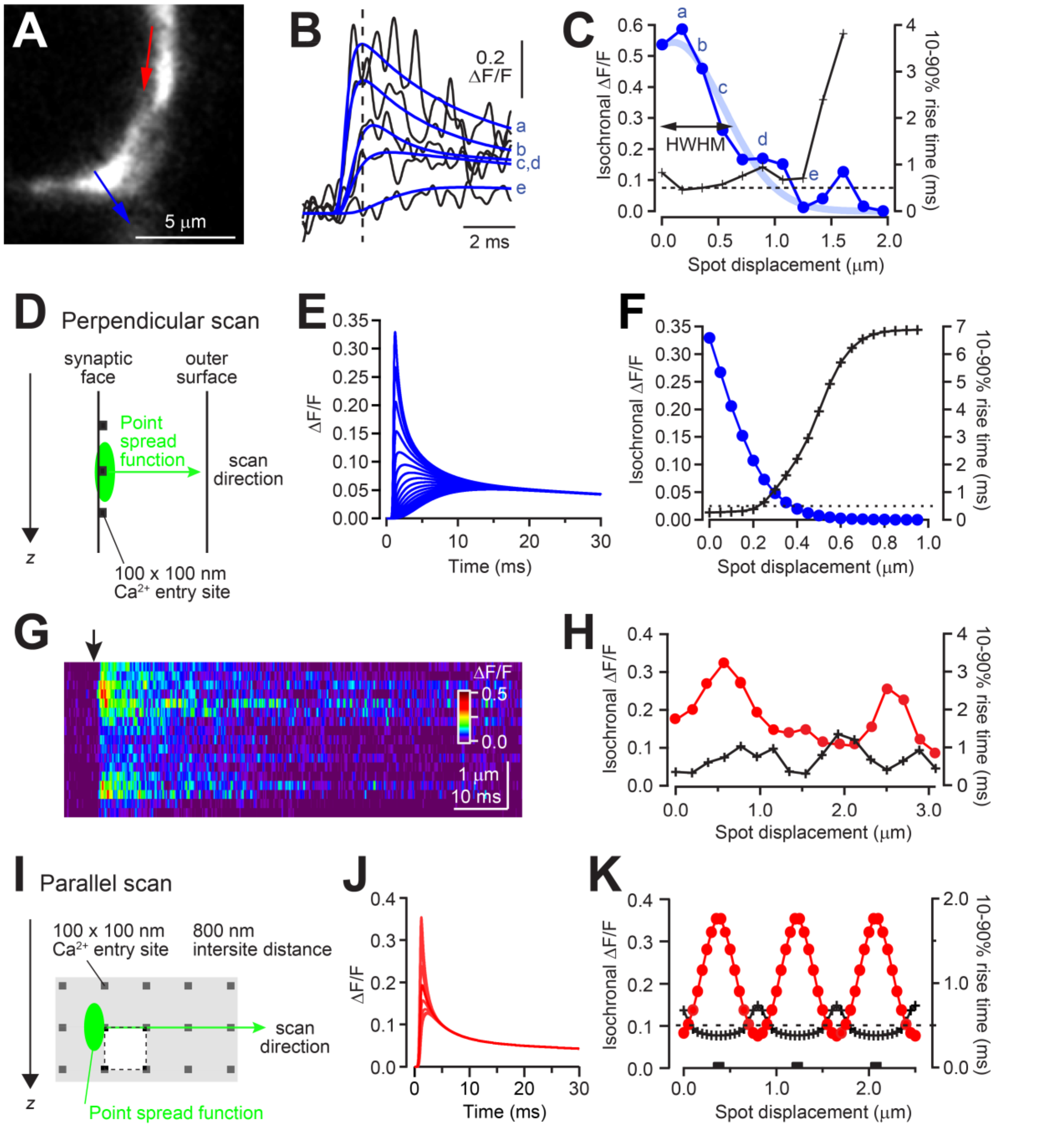

Figure S4. Spatial Dependence of Measured and Simulated AP-evoked Ca^{2+} Transients

(A) Confocal spot recording locations using a systematic spacing perpendicular to (blue arrow for B and C) and along (red arrow for G and H) the synaptic face of a representative P8 calyx.

(B) Representative Ca^{2+} transients (black traces) recorded at locations displaced from synaptic face, and their multi-exponential fits. A dashed line indicates the peak time of the largest transient. The amplitude of each fitted trace at this time point was used for the isochronal $\Delta F/F$ plot.

(C) Isochronal $\Delta F/F$ amplitudes (left axis) and 10-90% rise time (right axis) of Ca^{2+} transients plotted against the distance of spot displacement (178 nm steps) away from the synaptic face. The thick blue line shows a Gaussian fit to data points, from which the 50% decay distance of $\Delta F/F$ half width at half maximal (HWHM) was estimated. Ca^{2+} transients exhibited a prominent amplitude reduction with spot displacement (50% reduction in amplitude at 0.7 ± 0.03 µm, *n* = 4 calyces). These results are consistent with the report that VGCC currents are exclusively localized at the synaptic face (Sheng et

al., 2012).

(D) A schematic figure showing the arrangement of Ca^{2+} entry-sites and confocal detection volume for numerical simulation of Ca^{2+} transient recording locations perpendicular to synaptic surface. The size of simulation field was 0.8 μm (*x*: thickness of the calyx) × 0.8 μm (*y*) × 1 μm (*z*), and divided into equal elementary voxel sizes of 50 nm cube for simulation purposes. Ca^{2+} entry site was simulated as a 100 × 100 nm square (to approximate cluster area in SDS-FRL experiments).

(E) Simulated Ca^{2+} transients from confocal spot locations separated by 50 nm and perpendicular to synaptic face (green arrow in D).

(F) Isochronal $\Delta F/F$ (blue circles) and 10-90% rise time (black crosses) of the simulated Ca^{2+} transients for perpendicular scan. Isochronal amplitudes were calculated over a 0.2 ms window around the peak of the largest transient.

(G) A kymograph showing confocal spot recorded $\Delta F/F$ traces along synaptic face from the P8 calyx. Each trace was recorded at locations separated by 193 μm. An arrow indicates the AP peak time.

(H) Isochronal $\Delta F/F$ amplitudes of Ca^{2+} transients (red circles) and 10-90% rise time (black crosses) of Ca^{2+} transients plotted against spot displacement along synaptic face.

(I) A schematic figure showing the arrangement of Ca^{2+} entry-sites and confocal detection volume for parallel scan in simulation. A dashed square indicates simulation volume.

(J) Simulated Ca^{2+} transients from confocal spot locations separated by 50 nm and parallel to the synaptic surface. Scan direction is shown as a green arrow in (I).

(K) Isochronal $\Delta F/F$ amplitude (red circles) and 10-90% rise time (black crosses) of the simulated Ca^{2+} transients from (J). Black bars show the locations of Ca^{2+} entry site. Dashed line indicates rise time criterion (0.5 ms).

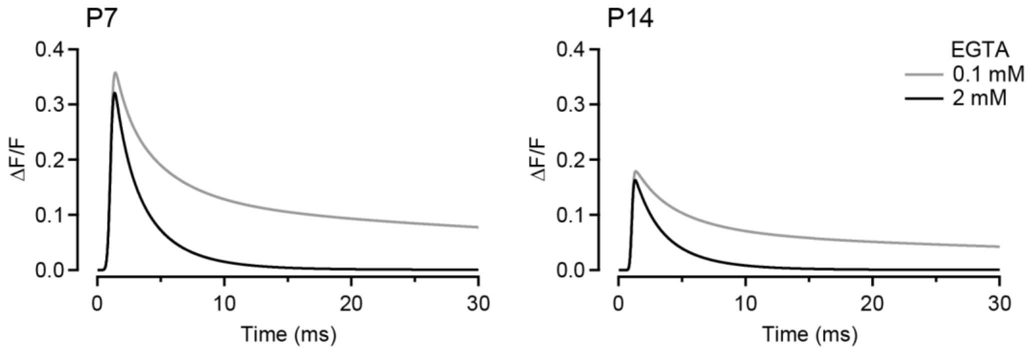

Figure S5. Simulation of Developmental Changes in AP-evoked Ca^{2+} Transients

Simulations of confocal spot detected Ca^{2+} transients at P7 and P14 using low-affinity endogenous fixed buffer (EFB, κ = 40) with 0.1 mM (gray) or 2 mM (black) EGTA in the internal solution. The Ca^{2+} diffusion model reproduced well the time course and amplitude of the experimentally measured Ca^{2+} transients at P7 and P14 (see Figures 4A and 4B). The number of open VGCCs was adjusted to match the amplitude of the peak $\Delta F/F$ between simulations and data (13 VGCCs for P7 and 11 VGCCs for P14).

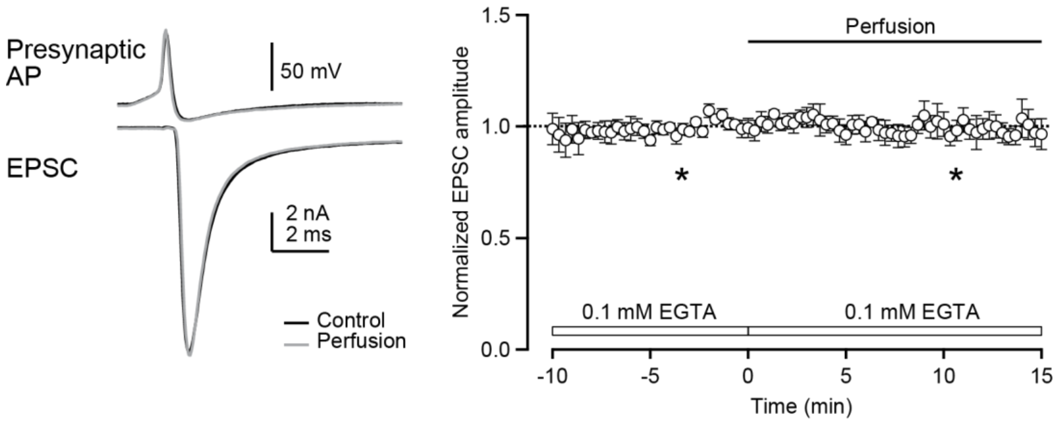

Figure S6. Pipette Perfusion Control Experiments

Left: Presynaptic AP and EPSCs 10 min after patch perfusion (gray trace) were indistinguishable from those recorded 5 min before patch perfusion (black trace, superimposed). *Right*: Time plot of EPSC amplitude when the internal solution of the same composition (0.1 mM EGTA) was perfused into the terminal at time zero. Data points are mean and SEM calculated from 7 experiments. The EPSC amplitudes were normalized to the mean EPSC amplitude calculated from EPSCs recorded during the 5 min before the onset of perfusion. Asterisks indicate the times at which sample records (left traces, superimposed) were collected.

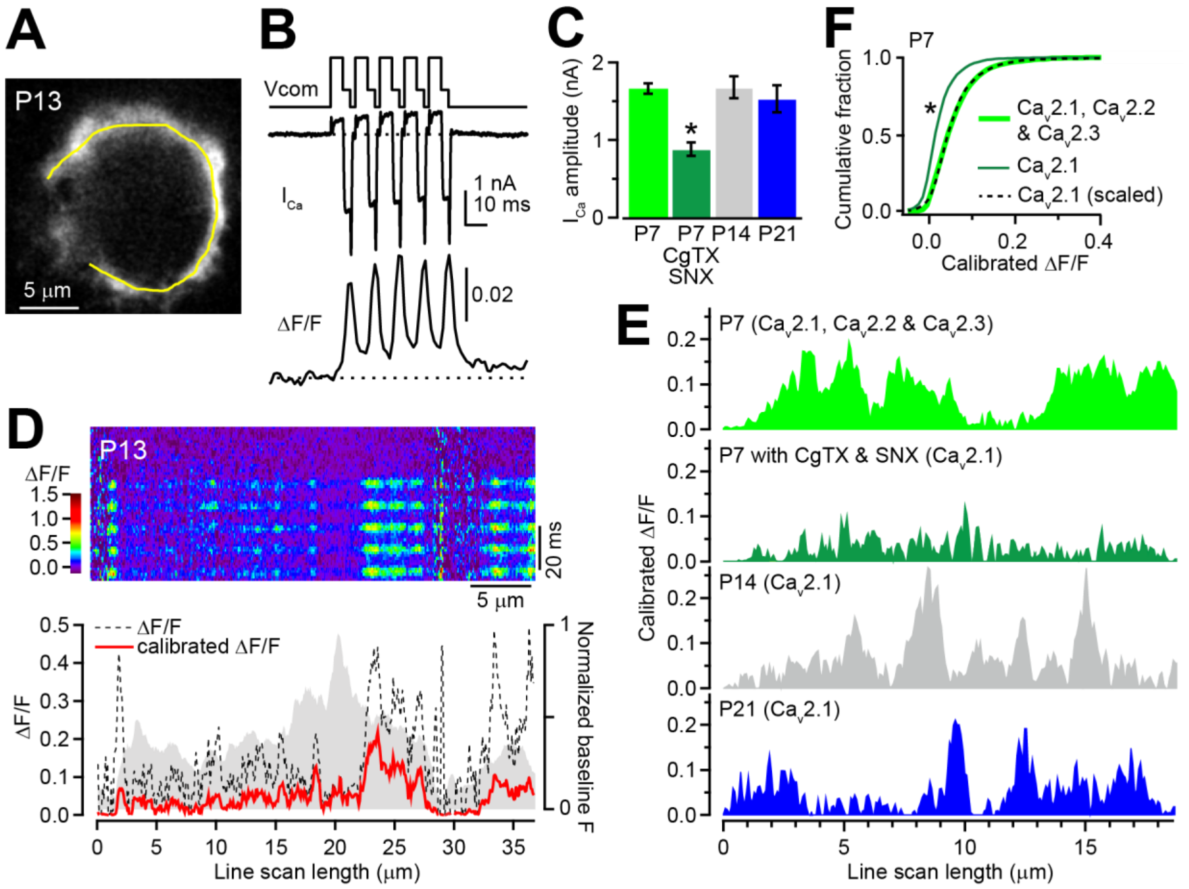

Figure S7. Spatial Distribution of Presynaptic Ca^{2+} Influx during Development

To examine the contribution of VGCC subtypes to the spatial distribution of Ca^{2+} influx, we performed confocal line scan Ca^{2+} imaging along the synaptic face of the calyx in response to voltage steps with and without subtype specific VGCC blockers. We used the low-affinity dye Oregon Green BAPTA-5N as a linear reporter of Ca^{2+} entry, and 10 mM EGTA to constrain the distribution of free Ca^{2+} to near Ca^{2+} entry sites.

(A) Confocal fluorescence image of a P13 calyx of Held loaded with 40 µM Alexa594 and 100 µM Oregon Green BAPTA-5N. Yellow line denotes the line scan region.

(B) Whole-terminal Ca^{2+} current (I_{Ca}, middle trace) evoked by voltage steps from –80 mV to +80 mV for 5 ms then to 0 mV for 3 ms (V_{com}, top trace). Bottom trace is a spatial average of the fluorescence change (ΔF/F; average of 3 trials) from the line scan in (D).

(C) The steady-state amplitude of I_{Ca} remained similar throughout development: at P7 (*n* = 23 calyces), P14 (*n* = 10), P21 (*n* = 6) and P7 calyces in the presence of CgTX and SNX (*n* = 11, $*P < 0.05$, one way ANOVA followed by Tuckey's post hoc between P7 (toxins) and all other groups).

(D) Linescan images (kymograph) and spatial profiles of ΔF/F exhibited hot spots of fluorescence changes along the scan region. *Top*: Average ΔF/F line scan (*n* = 3 trials) in response to the voltage step shown in (A). Acquisition rate was 1.2 ms/line. *Bottom*: Spatial distribution of resting fluorescence (gray, normalized to its maximum value), ΔF/F (dotted), and calibrated ΔF/F (red) along the line scan length. ΔF/F values were obtained from temporal averages of 3 ms during a depolarizing step to 0 mV and averaged from 15 such image segments (5 per scan × 3 line scan images). The calibrated the ΔF/F profile was used to normalize for terminal dimensions smaller than the microscope point spread

function, and calculated by dividing ΔF/F values at each pixel location by the relative resting fluorescence (calculated from the ratio of resting fluorescence at each spatial increment and the maximal resting fluorescence of the linescan).

(E) Spatial distribution of Ca^{2+} influx, expressed as calibrated ΔF/F, in a P7 calyx in the presence of CgTX and SNX, in a P14 calyx and in a P21 calyx.

(F) The spatial distribution was quantified using cumulative amplitude distributions of calibrated ΔF/F at P7 calyces in the absence (n = 39 scans from 23 calyces) and presence of CgTX and SNX (TX; n = 29 scans from 13 calyces). Dashed line is the ΔF/F distribution in the presence of toxins after normalizing by the median ΔF/F. There was no significant difference in the spatial distribution of Ca^{2+} entry with and without these toxins (P = 0.96, Kolmogorov-Smirnov test), suggesting that $Ca_v2.2$ and $Ca_v2.3$ have a similar distribution to $Ca_v2.1$, given our submicron resolution (see Figure S3).

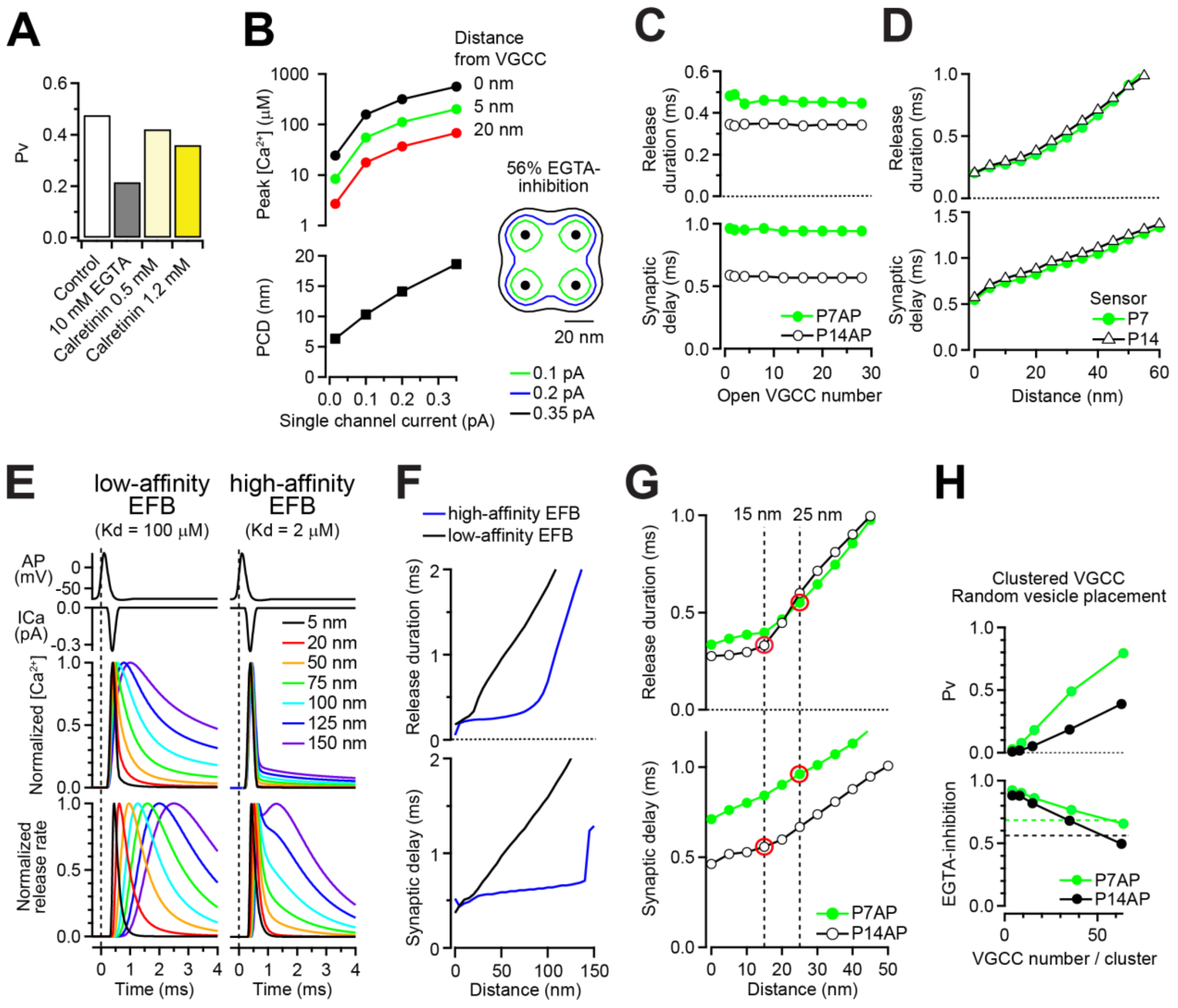

Figure S8. Influence of Model Parameters on Vesicular Release Simulations

(A) The effect of calretinin on P_v. P_v was estimated using four open VGCCs with an NND of 35 nm. For these simulations, we used rate constants for the independent T-site and R-site of calretinin (Table S2). The presence of calretinin at 1.2 mM (yellow bar) only had a modest effect on P_v with respect to control conditions.

(B) The influence of the single VGCC current amplitude on peak Ca^{2+} concentration (*top left*) and *perimeter coupling distance* (PCD; *bottom left*). Right panel indicates the EGTA-inhibition of P_v contour lines (56%) around the channel cluster composed of four open VGCCs with NND of 35 nm, assuming the single VGCC current amplitude as 0.35 pA (black), 0.2 pA (blue) and 0.1 pA (green). These simulations indicate that release is likely to occur from the perimeter of clusters for single channel conductances greater than ~0.2 pA.

(C) Synaptic delay and release duration plotted against the VGCC number within a cluster for perimeter release model simulations (PCD= 26-34 nm for P7 and 18-21 nm for P14). Although the number of open VGCC in response to AP decreased due to acceleration of AP from P7 to P14 (Yang and Wang, 2006), changing the number of open VGCCs had no effect on synaptic delay or release duration.

(D) Effects of developmental changes in Ca^{2+} sensitivity of vesicular sensors on release time course. Changes in Ca^{2+} sensor from P7 to the lower Ca^{2+} affinity sensor for P14 had little effect on release

duration and synaptic delay. Both sensor simulations were performed with an AP_7 waveform.

(E) Ca^{2+} reaction-diffusion and vesicular release simulation using a low-affinity (K_d = 100 μM, *left*) or high affinity EFB (K_d = 2 μM, *right*). For each condition, the time courses of Ca^{2+} transients (middle raw) and release rate (bottom raw) for different membrane voxels (distances from cluster perimeter are color-coded).

(F) Effects of EFB property on release duration and synaptic delay assuming high-affinity (blue trace) or low-affinity (black trace) EFB. The high-affinity buffer produces a release duration that is largely insensitive to PCD values between 10 and 100 nm.

(G) Dependence of release duration (upper panel) and synaptic delay (lower panel) on the PCD for P7 (green) and P14 (black), simulated using the perimeter release model with stochastic channel opening (Figure 7). Red circles indicate values predicted by experimental results.

(H) Initial P_v and EGTA-inhibition of P_v in random vesicle placement model. In this simulation, we assumed that synaptic vesicles primed for release were randomly distributed within AZ containing a single VGCC cluster. In this arrangement, the AZ P_v was calculated by simply averaging P_v from all locations within the AZ, assuming an area of 0.05 μm^2 (Taschenberger et al., 2002). Although the AZ P_v linearly increased with the number of open VGCC within the cluster, the mean number of VGCCs necessary to match experimental EGTA-inhibition values at P7 and P14 (dashed lines), was far greater than the mean number estimated from EM experiments (Figure 2E).

Table S1. Model Parameters for Simulations of Fluorescence Ca^{2+} Transients

Simulation Parameters	value	units	Reference
Simulation voxel size	50	nm	
Time step for simulation	0.38	μs	
Full width at half maximum of 3D Gaussian point spread function	220 (*x*-*y*)<br>700 (*z*)	nm	Figure S3
Ca^{2+} entry			
Maximal single channel current during AP	0.28 (P7)<br>0.35 (P14)	pA	Calculated from Sheng et al.,2012 and Figure 3F
Time course of Ca^{2+} entry			Taken from Figure 3F
Size of Ca^{2+} entry site	100 × 100	nm	Calculated from Figure 2C
Diffusion coefficient	0.22	$\mu m^2\ ms^{-1}$	Allbritton et al, 1992
Basal Ca^{2+} concentration	10	nM	Measured
Ca^{2+} extrusion	0.4	ms^{-1}	Helmchen et al, 1997
Endogenous fixed buffer properties			
k_{on}	100	$mM^{-1}ms^{-1}$	Xu et al., 1997
k_{off}	10	ms^{-1}	
Total concentration	4.0	mM	Calculated from Helmchen et al, 1997
ATP calcium binding properties			
k_{on}	500	$mM^{-1}ms^{-1}$	Naraghi & Neher, 1997
k_{off}	100	ms^{-1}	
Diffusion coefficient	0.2	$\mu m^2\ ms^{-1}$	
Total concentration available to bind Ca^{2+}	0.65	mM	calculated using Maxchelator
EGTA buffer properties			
k_{on}	10.5	$mM^{-1}ms^{-1}$	Nägerl et al., 2000
k_{off}	0.000735	ms^{-1}	
Diffusion coefficient	0.22	$\mu m^2\ ms^{-1}$	Naraghi & Neher, 1997
Total concentration	0.1, 2.0 or 10	mM	Experimental values
Calcium indicator (Oregon Green BAPTA-5N) properties			
k_{on}	240	$mM^{-1}ms^{-1}$	Faas et al., 2007
k_{off}	8.67	ms^{-1}	
Concentration	0.1	mM	Experimental value
Diffusion coefficient	0.1	$\mu m^2\ ms^{-1}$	Gabso et al., 1997
Ratio of maximum to minimum fluorescence	11.8		Bollmann and Sakmann, 2005

Table S2. Model Parameters for Simulations of Ca^{2+} Diffusion and Vesicular Release

Simulation Parameters	value	units	Reference
Simulation voxel size	5	nm	
Time step for Ca simulation	0.01515	μs	
Time step for release simulation	10	μs	
Ca^{2+} entry			
Maximal single channel current during AP	0.28 (P7)<br>0.35 (P14)	pA	Calculated from Sheng et al.,2012 and Figure 3F
Time course of Ca^{2+} entry			Taken from Figure 3F
Diffusion coefficient	0.22	$\mu m^2\ ms^{-1}$	Allbritton et al, 1992
Basal Ca^{2+} concentration	10	nM	Measured
Endogenous fixed buffer properties			
k_{on}	100	$mM^{-1}ms^{-1}$	Xu et al,. 1997
k_{off}	10	ms^{-1}	
Total concentration	4.0	mM	Calculated from Helmchen et al, 1997
ATP calcium binding properties			
k_{on}	500	$mM^{-1}ms^{-1}$	Naraghi & Neher, 1997
k_{off}	100	ms^{-1}	
Diffusion coefficient	0.2	$\mu m^2\ ms^{-1}$	
Total concentration available to bind Ca^{2+} (with 0.1 mM EGTA)	0.176	mM	calculated using Maxchelator
Total concentration available to bind Ca^{2+} (with 10 mM EGTA)	0.27	mM	
EGTA buffer properties			
k_{on}	10.5	$mM^{-1}ms^{-1}$	Nägerl et al., 2000
k_{off}	0.000735	ms^{-1}	
Diffusion coefficient	0.22	$\mu m^2\ ms^{-1}$	Naraghi & Neher, 1997
Total concentration	0.1 or 10	mM	Experimental values
Calretinin			
T site k_{on}	1.8	$mM^{-1}ms^{-1}$	Faas et al., 2007
T site k_{off}	0.053	ms^{-1}	
R site k_{on}	310	$mM^{-1}ms^{-1}$	
R site k_{off}	0.02	ms^{-1}	
Diffusion coefficient	0.02	$\mu m^2\ ms^{-1}$	Adopted the value for Calbindin D28k, Schmidt et al., (2005)
Total concentration	0.5 or 1.2	mM	Edmonds et al., 2000
Ca^{2+} sensor for release			
k_{on}	115 (P14)<br>121 (P8)	$mM^{-1}ms^{-1}$	Kochubey et al., 2009
k_{off}	7.9 (P14)<br>6.5 (P8)	ms^{-1}	
Cooperativity factor β	0.26		
Vesicular fusion rate γ	0.696	ms^{-1}	

SUPPREMENTAL EXPERIMENTAL PROCEDURES

Animals

Animal experiments were conducted in accordance with the guidelines of Doshisha University (Kyoto Japan), the National Institute for Physiological Sciences (Okazaki, Japan) and Institut Pasteur (Paris, France). Wistar rats and C57BL/6J mice of both sexes were raised on a 12 h light/dark cycle with water and food *ad libitum.*

Electron Microscopy and Analysis of SDS-Digested Freeze-Fracture Replica Labeling

SDS-FRL

Wistar rats at postnatal days (P) 7-8, 14-15, and 21 (*n* = 2, 4, and 5 animals, respectively) and P13 $Ca_v2.1$ knock-out (KO) and P13 wild-type mice were used. SDS-FRL was performed with some modifications (Masugi-Tokita and Shigemoto, 2007; Masugi-Tokita et al., 2007; Budisantoso et al., 2012; Indriati et al., 2013) of the technique developed by Fujimoto (Fujimoto, 1995). The rats were anesthetized with sodium pentobarbital (50 mg/kg, i.p.) and perfused transcardially with 25 mM phosphate buffered saline solution (PBS) for 1 min, followed by perfusion with 2% paraformaldehyde (PFA) and 15% saturated picric acid in 0.1 M phosphate buffer (PB) for 12 min. Coronal slices (130 μm thick) were cut using a vibrating microslicer (Pro7, Dosaka, Kyoto) in 0.1 M PB. A region of the MNTB was trimmed from the slices and immersed in graded glycerol of 10-30% in 0.1 M PB at 4°C 1 overnight and frozen by a high pressure freezing machine (HPM010; BAL-TEC). Frozen samples were fractured into two parts at -140°C and replicated by carbon deposition (5 nm thick), carbon-platinum (uni-direction from 60°, 2 nm) and carbon (20 nm) in a freeze-fracture replica machine (JFD II, JEOL, Tokyo). Tissue debris was dissolved with gentle shaking at 80°C for 18 h or 24 h in a solution containing 15 mM Tris-HCl (pH 8.3), 20% sucrose, and 2.5% SDS. The replicas were washed three times in 50 mM Tris-buffered saline (TBS, pH 7.4) containing 0.05% bovine serum albumin (BSA), 0.1% Tween-20, and 0.05% sodium azide and blocked with 5% BSA in the washing buffer for 1 h at room temperature. The replicas were then incubated with the guinea pig primary antibody against $Ca_v2.1$ subunit of P/Q type Ca^{2+} channel (8.1 μg/ml, Miyazaki et al., 2012) overnight at 15°C followed by incubation with goat anti-guinea pig secondary antibodies conjugated with 5 nm gold particles (British Biocell International, Cardiff) overnight at 15°C. Identification of 5 nm particles on the replicas was based on their size, round shape, and electron density. Weaker density dots just next to intra-membrane particles are mostly shadows made by the platinum coating and they were excluded. The specificity of the $Ca_v2.1$ antibody was confirmed by testing MNTB tissue from $Ca_v2.1$ KO mice. Immunogold particles for $Ca_v2.1$ were mostly abolished in the KO mice tissue (Figures S1A and S1B). For the double immunolabeling, primary antibody against RIM1/2 (Synaptic Systems, Holderith et al., 2012) combined with anti-rabbit secondary antibodies conjugated with 2 nm gold particles (British Biocell International) was used in a sequential manner after completing the labeling for $Ca_v2.1$. To facilitate visualization of these small gold particles, carbon replicas (Loukanov et al., 2010) were used for the double labeling. Background labeling for RIM estimated on the E-face was 0.32 /μm^2. The probability of having a single particle for RIM by chance within the 100 nm radius (green lines in Figure S2) of average $Ca_v2.1$ clusters was calculated to be less than 1%. The ratios of $Ca_v2.1$ clusters double labeled with at least a single RIM

particle were 64, 87, and 74% for P7 (n = 28), P14 (n = 79) and P21 (n = 100) samples, respectively.

Immunoparticle Distribution Analysis

Quantification of the distribution of immunoparticles was carried out as follows. Images of presynaptic P-face were captured at a magnification of 46,000× with a digital camera (Veleta, Olympus-Soft Imaging System; OSIS). Images were analyzed with iTEM (OSIS) and FIJI software (distributed under the General Public License, GPL). Distances were calculated using macros in Excel (Microsoft). The XY coordinates of immunoparticles were recorded and extracted in iTEM and the distances from each particle to every other particles were calculated and the smallest value was assigned as the nearest neighbor distance (NND) for each particle. To find clusters of immunoparticles, binary images with particle locations represented as single pixel dots were created and a 100 nm radius circle was drawn around each particle using the Maximum filter in FIJI. The particles were considered as forming a cluster when these circles overlap. When local maxima were searched in the binary images with overlapping circles, a geometrical centroid, also referred to as the center of gravity in this article, for each overlapping circle was found. The average particle distribution across all clusters was assessed by aligning individual distributions on their center of gravity (cluster center). The NND between clusters was estimated by determining the shortest distance between cluster centers.

Estimation of Labeling Efficiency

Labeling efficiency of $Ca_v2.1$ was estimated by comparing the overall density of the $Ca_v2.1$ immunogold particles with the amplitude of whole-terminal I_{Ca}. In response to a voltage step to 0 mV, the amplitude of steady-state whole-terminal I_{Ca} was 1691 pA at P14 (Figure S7C). When this amplitude was divided by the single channel current (0.15 pA) and the channel open probability for $Ca_v2.1$ channel at 0 mV (0.50, Sheng et al, 2012), this gave an estimate of 22546 channels per terminal. This number is similar to the estimate using variance mean analysis (Lin et al, 2011). We next estimated the number of gold particles corresponding to the entire calyx for both labeling samples. To do this, we measured surface area of postsynaptic MNTB neurons and their coverage by calyces. The surface area of MNTB neuron was 1168 ± 68 μm^2 (n = 11), similar to the value previously reported for the calyx of Held at P14 (Taschenberger et al., 2002). As calyx covers 55.8% of the surface (Taschenberger et al., 2002) the synaptic surface area was estimated as 647 μm^2.

In La1 (P14) samples the gold particle density was 6.7 /μm^2, giving a total number of $Ca_v2.1$ gold particles for the entire calyx of 4338. The labeling efficiency was therefore estimated to be 19% (4338/22546). In La2 (P14) the particle density was 21.7 /μm^2 giving a total gold particle number of 13984. The labeling efficiency for La2 was then estimated to be 62% (13984/22546). This labeling efficiency of La2 was similar to previously reported using the same batch of antibody (Indriati et al, 2013). As the NND within cluster was not different between P7, P14 and P21 in both La1 (Figure 2F) and La2 (data not shown), we assumed the same labeling efficiency across ages.

Slice Electrophysiology and Ca^{2+} Imaging

Brainstem Slice Preparation

Brainstem slices were prepared from P7-21 Wistar rats. Briefly, rats were decapitated under halothane anesthesia and their brains were quickly removed. Transverse slices (150-250 μm thick) containing the

MNTB were cut using a tissue slicer (VT1200S; Leica Microsystems). The dissections and slicing were performed in ice-cold Ca^{2+}-free artificial cerebrospinal fluid (ACSF) containing (in mM) 125 NaCl, 2.5 KCl, 26 $NaHCO_3$, 1.25 NaH_2PO_4, 6 $MgCl_2$, 10 glucose, 3 myo-inositol, 2 sodium pyruvate and 0.5 ascorbic acid (pH 7.4 when bubbled with 95% O_2 and 5% CO_2). Slices were maintained in the standard ACSF at 37°C for 40-60 min and subsequently at room temperature. The composition of standard ACSF was the same as the Ca^{2+}-free ACSF except that $MgCl_2$ and $CaCl_2$ concentrations were 1 mM and 2 mM, respectively.

Electrophysiological Recordings and Data Analysis

Whole-cell patch-clamp recordings from calyces were made using Multiclamp 700A or Multiclamp 700B amplifier (Molecular Devices). All experiments were carried out at room temperature (22-24 ^{o}C). Patch pipettes were made from glass capillaries (GC150F-10, Harvard Apparatus, Kent) using a puller (P-1000, Sutter). For electrophysiological recordings combined with confocal presynaptic Ca^{2+} imaging, the pipette solution contained (in mM): 95 K-methanesulfonate, 30 KCl, 40 HEPES, 0.1 or 2 EGTA, 6 NaOH, 4 $MgCl_2$, 4 ATP-Na, 0.5 GTP-Na (pH adjusted to 7.3 with KOH, 295-305 mOsm), to which we added Oregon Green BAPTA 5N (100 μM, Invitrogen) for Ca^{2+} imaging and Alexa 594 (40 μM, Invitrogen) for visualizing the shape of calyces. In presynaptic voltage-clamp experiments, K-methanesulfonate and KCl were replaced with (in mM) 125 CsCl and 10 TEA-Cl. Sodium and potassium currents were blocked by adding tetrodotoxin (1 μM), TEA-Cl (10 mM) and 4-aminopirdine (100 μM) to perfusate. In line scan experiments (Figure S7), [EGTA] was raised to 10 mM to minimize diffusional blurring of [Ca^{2+}] and Ca^{2+}-dependent VGCC inactivation at P7 (Nakamura et al., 2008). For simultaneous pre- and postsynaptic patch-clamp recordings, the presynaptic pipette solution contained (in mM): 90 K-methanesulfonate, 30 KCl, 3 K-glutamate, 40 HEPES, 12 Na_2 creatinephosphate, 0.1 EGTA, 1 $MgCl_2$, 2 ATP-Mg, 0.5 GTP-Na (pH adjusted to 7.3 with KOH, 315 mOsm). Free Ca^{2+} concentration in all presynaptic solutions was less than 10 nM (calculated using Maxchelator #46v8 (http://maxchelator.stanford.edu/), which was confirmed by fluorescent calibration using Oregon Green BAPTA1 (Invitrogen). The presynaptic pipette and series resistance were 4.5–7.0 MΩ and 6–22 MΩ, respectively, and routinely compensated by 80%. In voltage clamp experiments, linear leak and capacitive currents were subtracted using the scaled pulse (P/8) protocol. The liquid junction potential was not corrected. Presynaptic APs were evoked by brief current injection via a presynaptic pipette or by presynaptic fiber stimulation using an extracellular bipolar tungsten electrode.

The presynaptic pipette perfusion was performed as described previously (Takahashi et al, 2012). Briefly, under stereoscopic microscope observation, thin glass capillary (PT-030, Takao Manufacturing, Kyoto) containing the presynaptic patch solution with 10 mM EGTA was inserted into the presynaptic patch pipette. We set the distance between the tip of thin capillary and that of presynaptic patch pipette to be less than 200 μm. The tip of the inner glass capillary was connected to a picospritzer (Parker, Cleaveland) via tubing, from which we controlled the pressure (typically 8-10 psi) and timing of intra-terminal dialysis.

Electrodes for postsynaptic MNTB neurons had a resistance of 2.5-4.5 MΩ with the pipette solution containing (in mM): 110 Cs-methanesulfonate, 30 CsCl, 10 HEPES, 5 EGTA, 5 QX314 and 1 $MgCl_2$ (310 mOsm/l, pH 7.3 adjusted with CsOH). The access resistance was 6–20 MΩ, which was compensated by 80%. EPSCs were recorded from MNTB principal neurons at a holding potential of -70

mV. 100 μM Picrotoxin (100 μM) and strychnine (0.5 μM) were used to block spontaneous inhibitory postsynaptic currents. ω-Conotoxin-GIVA and SNX-482 (Peptide Institute, Osaka) were bath-applied with cytochrome-C (0.1 mg/ml).

Unless otherwise noted all chemicals and salts were from Sigma (St. Louis, MO) or Nacalai (Kyoto, Japan). QX314 was purchased from Almone Labs (Jerusalem). Tetrodotoxin was from Abcam (Cambridge, UK). Electrophysiological recordings were low-pass filtered at 10 kHz and acquired at 50-100 kHz (6052E, National Instruments or Digidata 1320A, Molecular Devices). Data analysis was performed with IgorPro 6.3 (WaveMetrics) using NeuroMatic software (http://www.neuromatic.thinkrandom.com/), or with Excel. Residual series resistance errors were compensated off-line for all evoked EPSCs. The synaptic delay was estimated from the 50% rise of the presynaptic AP to the 20% rise of the EPSC. All values in the text and figures are given as means ± SEM unless otherwise indicated. Statistical comparisons were made using the Student's unpaired t-test unless otherwise noted. $P < 0.05$ was considered as significant.

Time domain deconvolution of AP-evoked EPSCs was performed as describe previously (Neher and Sakaba., 2001; Taschenberger et al., 2005). We avoided use of low-affinity AMPAR antagonists because they inhibit mEPSC, precluding their use as a quantal response template for release rate estimates by deconvolution (see below). Cyclothiazide was also omitted due to its effects on presynaptic Ca^{2+} and K^+ currents (Ishikawa and Takahashi, 2001). For the EPSC deconvolution, matched quantal responses were used at each synapse. Quantal templates were extracted using triple exponential fits to average mEPSC waveform.

Measurements and Analysis of Ca^{2+} Transients

Ca^{2+}-dependent fluorescence transients were recorded using confocal laser scanning and spot detection using an Ultima scanning head (Prairie Technologies) mounted on an Olympus BX61W1 microscope. An acousto-optical tunable filter (AOTF) was used to select the excitation wavelength, amplitude and duration of illumination from 488 (Omicron Laserage) and 594 nm (Cobalt Lasers) diode lasers, for excitation of Oregon Green BAPTA-5N and Alexa 594, respectively. The laser beam was collimated and adjusted to overfill a 1.1 NA 60× objective (LUMFLN60XW, Olympus), thereby forming a. a diffraction-limited illumination spot in the specimen plane. Emitted fluorescence was descaned, and aligned through a 60 µm pinhole, placed on a conjugate image plane (corresponding to ~0.5 Airy units). Fluorescence emission from Oregon Green BAPTA-5N was filtered using a 535/70 nm band pass filter and detected with a gallium arsenide phosphide-based photocathode photomultiplier tube (H7422P, Hamamatsu Photonics). Alexa 594 fluorescence was filtered with a 605LP filter (all filters were from Chroma) and detected with a side-on multi-alkali PMT (3896, Hamamatsu Photonics). Zoom factor was set such that pixel sizes were 80 nm.

Confocal spot-detected fluorescence signals were filtered at 10 kHz using an 8-pole Bessel filter (Frequency Devices), digitized at 100 kHz, then filtered offline at 2 kHz. The jitter in the AP onset was corrected by time-aligning at the AP peak. The magnitude of Ca^{2+} transients was expressed as $\Delta F/F$ of Oregon Green BAPTA-5N fluorescence signal, which was calculated according to the equation:

$$\Delta F/F\ (t) = (F(t) - F_{rest}) / F_{rest} \quad \text{(equation 1)}$$

where F_{rest} is the time averaged (10 ms window) fluorescence before stimulation and $F(t)$ is the time-dependent fluorescence transient. $F(t)$ and F_{rest} values were calculated from the raw PMT signal

by subtracting the signal due to background fluorescence (i.e. due to PMT signal in the dark and that due to auto-fluorescence of tissue).The presence of Ca^{2+} transients was confirmed if the peak amplitude of ΔF/F trace was 3× larger than baseline root mean square, and such traces were further analyzed as shown below. To estimate the amplitude and rise time of Ca^{2+} transients, we fit single or averaged *ΔF/F* traces with the following equation (Nielsen et al., 2004), a least-square algorithm implemented in IgorPro:

$$\Delta F/F(t) = A_1\left(1-\exp\left(\frac{t-t_0}{\tau_{rise}}\right)\right)^n \left(A_2\exp\left(\frac{t-t_0}{\tau_{decay1}}\right)+A_3\exp\left(\frac{t-t_0}{\tau_{decay2}}\right)\right) \quad \text{(equation 2)}$$

For kinetic comparison, we selected transients for further analysis according to a rise time criterion of less than 0.5 ms. For spot locations separated by <0.2 um, only the largest trace were chosen within an 0.5 µm window (local maximum). The decay phase of *ΔF/F* traces were fitted by a double exponential decay function according to:

$$\Delta F/F(t) = A_1\exp\left(\frac{t-t_0}{\tau_{decay1}}\right)+A_2\exp\left(\frac{t-t_0}{\tau_{decay2}}\right) \qquad \text{(equation 3)}$$

where A_1 and A_2, and τ_{decay1} and τ_{decay2} are the relative amplitudes and time constants, respectively, of the fits. The weighted decay was calculated from the weighted average of the two time constants of a double exponential fit of the Ca^{2+} transient decay $(\tau_{decay1} A_1 + \tau_{decay2} A_2) / (A_1 + A_2)$.

For linescan-based fluorescence detection the acquisition rate for each line was typically 0.9-1.5 ms. To estimate the spatial distribution of Ca^{2+} entry, we calculated *ΔF/F* from a 3 ms window during the depolarizing step to 0 mV. This spatial profile of *ΔF/F* was calibrated by dividing by the ratio of F_{rest} per pixel and the maximum F_{rest} of the scan. The maximum F_{rest} was assumed to occur when point spread function was fully included within the nerve terminal. This procedure minimized errors caused by the calyx volume smaller than the detection volume and provided a more accurate estimate of the location and size of Ca^{2+} entry sites.

Numerical Simulations of Ca^{2+} Reaction-Diffusion and Vesicular Release

Simulations of Fluorescence Ca^{2+} Transients

We used D3D, a Java-based 3D reaction-diffusion simulator that was developed from earlier simulator implementations (Nielsen et al., 2004; DiGregorio et al., 2007) running on a Windows 7 operating system to calculate the spatiotemporal distribution of $[Ca^{2+}]$ in the vicinity of VGCC clusters at a calyx of Held terminal. Ca^{2+} diffusion and binding with Oregon Green BAPTA-5N and buffers were simulated by numerically integrating differential equations using an explicit finite-difference (Euler) method with a fixed time step and an elementary integration volume (i.e. voxels) (DiGregorio et al., 1999). In order to speed the calculation, we used simulation voxels that were 50 x 50 x 50 nm. The predicted Ca^{2+} transient decay time courses did not differ if the voxel size was reduced to 5 x 5 x 5 nm. The total simulation volume was 0.8 (*x*) × 0.8 (*y*) × 1.0 (*z*) µm, with the Ca^{2+} entry site centered within a surface orthogonal to the *z*-dimension. The height (*z*) matches the thickness of the terminal (Sätzler et al., 2002), while the *x* and *y* dimensions correspond to the average NND between clusters (Figure 1E), which with reflective boundaries on the four surfaces orthogonal to the *x*-*y* dimensions, is mathematically equivalent to periodic symmetrically arranged sources. In some simulations we took

advantage of quarter symmetry in the *x*-*y* plane to reduce the simulation volume by one quarter. The time step was calculated according to a stability criterion determined by the following equation:

$$h = {(3 * D * dt)}\big/{\partial x^2},$$

where *D* is the diffusion coefficient of the fastest diffusant, *dx* the voxel width and *h* the stability factor (0.1-0.4). Ca^{2+} extrusion via active transport was included on both *z* surfaces at a rate of 400 Ca^{2+}/s (Helmchen et al, 1997).

To model Ca^{2+} entry, the simulation volume contained a single 100 × 100 nm Ca^{2+} entry site. The total I_{Ca}, equivalent to the summed current from open VGCCs within a cluster, was homogenously distributed over the Ca^{2+} entry site, a size mimicking the average VGCC cluster area (Figure 2C). The total Ca^{2+} influx was varied by adjusting the equivalent number of open VGCCs to reproduce the amplitude of measured Ca^{2+} transient (Figures 4A and 4B). The time course of whole-terminal I_{Ca} in response to a voltage command waveform of a presynaptic AP (Figure 3F) was used as the waveform of Ca^{2+} entry. Because developmental shortening in the presynaptic AP results in a different Ca^{2+} driving force during the AP, we estimated the driving force for P7 and P14 conditions as the difference between the Ca^{2+} reversal potential (+45 mV, Sheng et al, 2012) and the potential of the AP waveform at the time of maximal I_{Ca} (Figure 3F). The computed Ca^{2+} driving force along with the single VGCC conductance (Sheng et al., 2012) resulted in a single VGCC current equaling 0.28 pA for P7 and 0.35 pA for P14 conditions. The resulting I_{Ca} had a half-duration of 0.28 ms for P7 and 0.19 ms for P14 conditions.

The concentration of Ca^{2+}-bound Oregon Green BAPTA-5N was converted to a fluorescence change ΔF/F as described by equation 6 in DiGregorio et al. (1999). To compare to experimental data, ΔF/F values were weighted according to a 3D Gaussian function matching the lateral and axial full width at half maximal of the measured confocal point spread function (Figure S3). The Gaussian volume was positioned in different locations within the simulation volumes by adding an *x*, *y*, or *z* offset to the 3D function (Figures S4D and S4I). Point spread function locations that produced transients with fast rise times (<0.5 ms) were then averaged together to produce a final simulated ΔF/F transient (0.1 mM EGTA) that was compared directly to data. The same spot locations were used to produce the average simulated ΔF/F transient in 2 mM EGTA. For confocal spot locations corresponding to the synaptic surface, the point spread function was centered 100 nm from the *z* surface containing the Ca^{2+} entry site. Spatial dependence along the synaptic face (parallel scan) and perpendicular to the face was achieved by displacing the center of the point spread function in the *x* or *z* dimension, respectively (Figure S4). Simulation parameters are summarized in Table S1.

Simulations of Nanoscale [Ca^{2+}] and Vesicular Release

To simulate Ca^{2+} reaction-diffusion near the vicinity of VGCC clusters on the nanoscale, we used the D3D simulation environment with a smaller voxel size (5 nm cubes). To reduce the total simulation time, we used a smaller simulation volume of 0.5 (*x*) × 0.5 (*y*) × 1.0 (*z*) μm. We confirmed that this smaller volume generated a similar result to that of the larger volume and therefore had no effect on our PCD estimate (data not shown). We also omitted the Ca^{2+} extrusion from these simulations, because the Ca^{2+} extrusion rate is so slow that it does not make significant contribution during the 10 ms simulation duration. For the P7 simulations, we assumed a VGCC open probability = 0.25 during a single AP

(Sheng et al, 2012) estimated using single channel recordings. For the P14 simulations, open probability was scaled down by 30% to 0.175 to account for the shorter AP duration at P14 (Yang and Wang, 2006).

For simulations of fixed VGCC open patterns (Figures 6 and 8), we modeled only the open VGCCs during an AP. The open channel number and density is assumed to equal that of gold particles per cluster of La1 samples, because the VGCC open probability is similar to the labeling efficiency of La1 (19%). We used a grid distribution of the VGCCs with a NND of 35 nm, which is similar to the NND of the gold particles measured in the La1 samples (37 nm). In some cases we directly used the immunogold particle locations of La1 clusters to define the open VGCC positions. The transmitter release rate was calculated using a 5-state Ca^{2+}-dependent release model for developing rats (Kochubey et al, 2009), together with the simulated $[Ca^{2+}]$ time course ($[Ca^{2+}](t)$) computed at each 5 nm voxel in Igor using the Euler method. The vesicular release probability (P_v) was calculated from the integral of the release rate. EGTA-inhibition of P_v was estimated by examining the simulated P_v at a particular voxel when simulated $[Ca^{2+}](t)$ waveforms in 0.1 or 10 mM EGTA. For each cluster we estimated the PCD, defined as the average distance between the voxels exhibiting experimental EGTA-inhibition values (Figure 5F) and the nearest VGCC. Contour lines were drawn using the implemented function of Igor. P_v for each VGCC cluster was the mean P_v over voxels at the PCD in the presence of 0.1 mM EGTA. For the analysis of release kinetics, we used results from the fixed pattern of VGCC opening simulations. Similar results were obtained using other age-dependent models in mice (Wang et al., 2008).

For the simulations with stochastic patterns of VGCC opening (Figure 7), we used a grid distribution of VGCC with a NND of 25 nm, similar to the gold particle NND of La2. Different patterns of open VGCCs were generated on each trial by assuming that the each VGCC was independent and had an equal open probability during AP (specific for that each age). We computed $[Ca^{2+}](t)$ waveforms at each voxel for each trial, which were then used to estimate the fraction of sensors in the release state at each voxel per trial. P_v was then calculated from the average fraction of sensors in the release state across 50 trials. EGTA-inhibition of P_v was then calculated from the fractional reduction in P_v in the 0.1 mM and 10 mM EGTA simulations.

Simulation parameters are summarized in Table S2. The k_{on} value reported for EGTA by Naraghi (1997; $2.7\times 10^6\ M^{-1}s^{-1}$ at pH 7.2) is one order of magnitude slower than that by Nägerl et al., (2000; $1.0 \times 10^7\ M^{-1}s^{-1}$ at pH 7.3, also see Neher, 1986). We adopted the latter value because the magnitude of EPSC-inhibition by 10 mM EGTA was similar to that by 0.5-1 mM BAPTA (k_{on}, $4.5 \times 10^8\ M^{-1}s^{-1}$) at P14 calyces (data not shown), suggesting 10-20 times, not 170 times (Naraghi, 1997), difference in k_{on} between EGTA and BAPTA.

SUPPREMENTAL REFERENCES

Allbritton, N. L., Meyer, T., and Stryer, L. (1992). Range of messenger action of calcium ion and inositol 1,4,5- trisphosphate. Science *258*, 1812-1815.

Bollmann, J.H., and Sakmann, B. (2005). Control of synaptic strength and timing by the release-site Ca^{2+} signal. Nat Neurosci *8*, 426-434.

Budisantoso, T., Harada, H., Kamasawa, N., Fukazawa, Y., Shigemoto, R., and Matsui, K. (2013). Evaluation of glutamate concentration transient in the synaptic cleft of the rat calyx of Held. J Physiol *591*, 219-239.

DiGregorio, D.A., Peskoff, A., and Vergara, J.L. (1999). Measurement of action potential-induced presynaptic calcium domains at a cultured neuromuscular junction. J Neurosci *19*, 7846-7859.

DiGregorio, D.A., Rothman, J.S., Nielsen, T.A., and Silver, R.A. (2007). Desensitization properties of AMPA receptors at the cerebellar mossy fiber granule cell synapse. J Neurosci *27*, 8344-8357.

Edmonds, B., Reyes, R., Schwaller, B., and Roberts, W.M. (2000). Calretinin modifies presynaptic calcium signaling in frog saccular hair cells. Nat Neurosci *3*, 786-790.

Faas, G.C., Schwaller, B., Vergara, J. L., and Mody, I. (2007). Resolving the fast kinetics of cooperative binding: Ca^{2+} buffering by Calretinin. Plos Biolgy *5*, e311.

Fujimoto, K. (1995). Freeze-fracture replica electron microscopy combined with SDS digestion for cytochemical labeling of integral membrane proteins. Application to the immunogold labeling of intercellular junctional complexes. J Cell Sci *108*, 3443-3449.

Gabso, M., Neher, E., and Spira, M. E. (1997). Low mobility of the Ca^{2+} buffers in axons of cultured Aplysia neurons. Neuron *18*, 473-481.

Helmchen, F., Borst, J.G., and Sakmann, B. (1997). Calcium dynamics associated with a single action potential in a CNS presynaptic terminal. Biophys J *72*, 1458-1471.

Holderith, N., Lorincz, A., Katona, G., Rozsa, B., Kulik, A., Watanabe, M., and Nusser, Z. (2012). Release probability of hippocampal glutamatergic terminals scales with the size of the active zone. Nat Neurosci *15*, 988-997.

Indriati, D.W., Kamasawa, N., Matsui, K., Meredith, A.L., Watanabe, M., and Shigemoto, R. (2013). Quantitative localization of $Ca_v2.1$ (P/Q-type) voltage-dependent calcium channels in Purkinje cells: somatodendritic gradient and distinct somatic coclustering with calcium-activated potassium channels. J Neurosci *33*, 3668-3678.

Ishikawa, T., and Takahashi, T. (2001). Mechanisms underlying presynaptic facilitatory effect of cyclothiazide at the calyx of Held of juvenile rats. J Physiol *533*, 423-431.

Kochubey, O., Han, Y., and Schneggenburger, R. (2009). Developmental regulation of the intracellular Ca^{2+} sensitivity of vesicle fusion and Ca^{2+}-secretion coupling at the rat calyx of Held. J Physiol *587*, 3009-3023.

Lin, K.H., Oleskevich, S., and Taschenberger, H. (2011). Presynaptic Ca^{2+} influx and vesicle exocytosis at the mouse endbulb of Held: a comparison of two auditory nerve terminals. J Physiol *589*, 4301-4320.

Loukanov A., Kamasawa N., Danev R., Shigemoto R., and Nagayama K. (2010). Immunolocalization of multiple membrane proteins on a carbon replica with STEM and EDX. Ultramicroscopy *110*, 366-374,

Masugi-Tokita, M., and Shigemoto, R. (2007). High-resolution quantitative visualization of glutamate and GABA receptors at central synapses. Curr Opin Neurobiol *17*, 387-393.

Masugi-Tokita, M., Tarusawa, E., Watanabe, M., Molnár, E., Fujimoto, K., and Shigemoto, R. (2007). Number and density of AMPA receptors in individual synapses in the rat cerebellum as revealed by SDS-digested freeze-fracture replica labeling. J Neurosci *27*, 2135-2144.

Miyazaki, T., Yamasaki, M., Hashimoto, K., Yamazaki, M., Abe, M., Usui, H., Kano, M., Sakimura, K., and Watanabe, M. (2012). $Ca_v2.1$ in cerebellar Purkinje cells regulates competitive excitatory

synaptic wiring, cell survival, and cerebellar biochemical compartmentalization. J Neurosci *32*, 1311-1328.

Nägerl, U. V., Novo, D., Mody, I., and Vergara, J. L. (2000). Binding kinetics of calbindin-D(28k) determined by flash photolysis of caged Ca^{2+}. Biophys J *79*, 3009-3018.

Naraghi, M. (1997) T-jump study of calcium binding kinetics of calcium chelators. Cell Calcium *22*, 255-268

Naraghi, M., and Neher, E. (1997). Linearized buffered Ca^{2+} diffusion in microdomains and its implications for calculation of [Ca^{2+}] at the mouth of a calcium channel. J Neurosci *17*, 6961-6973.

Nakamura, T., Yamashita, T., Saitoh, N., and Takahashi, T. (2008). Developmental changes in calcium/calmodulin-dependent inactivation of calcium currents at the rat calyx of Held. J Physiol *586*, 2253-2261.

Neher, E. (1986) Concentration profiles of intracellular calcium in the presence of a diffusible chelator. Exp Brain Res *14*, 80-96.

Nielsen, T.A., DiGregorio, D.A., and Silver, R.A. (2004). Modulation of glutamate mobility reveals the mechanism underlying slow-rising AMPAR EPSCs and the diffusion coefficient in the synaptic cleft. Neuron *42*, 757-771.

Sakaba, T., and Neher, E. (2001). Quantitative relationship between transmitter release and calcium current at the calyx of Held synapse. J Neurosci *21*, 462-476.

Sätzler, K., Söhl, L.F., Bollmann, J.H., Borst, J.G.G., Frotscher, M., Sakmann, B., and Lübke, J.H. (2002). Three-dimensional reconstruction of a calyx of Held and its postsynaptic principal neuron in the medial nucleus of the trapezoid body. J Neurosci *22*, 10567-10579.

Schmidt, H., Schwaller, B. and Eilers, J. (2005) Calbindin D28k targets myo-inositol monophosphatase in spines and dendrites of cerebellar Purkinje neurons. Proc Natl Acad Sci U S A *102*, 5850–5855

Sheng, J., He, L., Zheng, H., Xue, L., Luo, F., Shin, W., Sun, T., Kuner, T., Yue, D.T., and Wu, L.G. (2012). Calcium-channel number critically influences synaptic strength and plasticity at the active zone. Nat Neurosci *15*, 998-1006.

Takahashi,T., Hori, T., Nakamura, Y., and Yamashita,T. (2012). Patch-clamp recording method in slices for studying presynaptic mechanisms. In Patch Clamp Techniques, Y Okada, ed. (Springer) pp. 137-145.

Taschenberger, H., Leão, R.M., Rowland, K.C., Spirou, G.A., and von Gersdorff, H. (2002). Optimizing synaptic architecture and efficiency for high-frequency transmission. Neuron *36*, 1127-1143.

Taschenberger, H., Scheuss, V., and Neher, E. (2005). Release kinetics, quantal parameters and their modulation during short-term depression at a developing synapse in the rat CNS. J Physiol *568*, 513-537.

Wang, L.Y., Neher, E., and Taschenberger, H. (2008). Synaptic vesicles in mature calyx of Held synapses sense higher nanodomain calcium concentrations during action potential-evoked glutamate release. J Neurosci *28*, 14450-14458.

Xu, T., Naraghi, M., Kang, H., and Neher, E. (1997). Kinetic studies of Ca^{2+} binding and Ca^{2+} clearance in the cytosol of adrenal chromaffin cells. Biophys J *73*, 532-545.

Yang, Y.M., and Wang, L.Y. (2006). Amplitude and kinetics of action potential-evoked Ca^{2+} current and its efficacy in triggering transmitter release at the developing calyx of Held synapse. J Neurosci *26*, 5698-5708.
