# Supplementary material for: Nanoscale Distribution of Presynaptic Ca2+ Channels and Its Impact on Vesicular Release during Development
Source: Neuron. 2015 Jan 7;85(1):145–58. doi: 10.1016/j.neuron.2014.11.019 (PMC4305191; doi:10.1016/j.neuron.2014.11.019)
Supplement: Document S2. Article plus Supplemental Information [file mmc2.pdf]

Article

Neuron

Nanoscale Distribution of Presynaptic Ca^{2+} Channels and Its Impact on Vesicular Release during Development

Highlights

- Ca^{2+} channels form clusters with highly variable numbers of channels
- EGTA sensitivity suggests that synaptic vesicles are tightly coupled to clusters
- Ca^{2+} channel number per cluster alters synaptic efficacy, but not precision
- A perimeter model accounts for synaptic efficacy and precision during development

Authors

Yukihiro Nakamura, Harumi Harada, ..., David A. DiGregorio, Tomoyuki Takahashi

Correspondence

david.digregorio@pasteur.fr (D.A.D.),
ttakahas@mail.doshisha.ac.jp (T.T.)

In Brief

Nanoscale topography of presynaptic Ca^{2+} channels and synaptic vesicles critically influences synaptic transmission. Nakamura et al. find that action potentials evoke release of vesicles coupled to the perimeter of Ca^{2+} channel clusters, explaining synaptic efficacy and precision during development.

Nakamura et al., 2015, Neuron 85, 145–158
January 7, 2015 ©2015 The Authors
http://dx.doi.org/10.1016/j.neuron.2014.11.019

Nanoscale Distribution of Presynaptic Ca^{2+} Channels and Its Impact on Vesicular Release during Development

Yukihiro Nakamura,[1,2,3,4] Harumi Harada,[5,6] Naomi Kamasawa,[5,8] Ko Matsui,[5,9] Jason S. Rothman,[7] Ryuichi Shigemoto,[5,6] R. Angus Silver,[7] David A. DiGregorio,[3,4,*] and Tomoyuki Takahashi[1,2,*]
[1]Laboratory of Molecular Synaptic Function, Graduate School of Brain Science, Doshisha University, Kyoto 610-0394, Japan
[2]Cellular & Molecular Synaptic Function Unit, Okinawa Institute of Science and Technology (OIST) Graduate University, Okinawa 904-0495, Japan
[3]Laboratory of Dynamic Neuronal Imaging, Institut Pasteur, 25 rue du Dr Roux, 75724 Paris Cedex 15, France
[4]CNRS UMR 3571, 25 rue du Dr Roux, 75724 Paris Cedex 15, France
[5]Division of Cerebral Structure, Department of Cerebral Research, National Institute for Physiological Sciences, Myodaiji, Okazaki 444-8787, Japan
[6]Institute of Science and Technology Austria, A-3400 Klosterneuburg, Austria
[7]Department of Neuroscience, Physiology and Pharmacology, University College London, Gower Street London WC1E 6BT, UK
[8]Present address: Electron Microscopy Facility, Max Planck Florida Institute for Neuroscience, Jupiter, FL 33458, USA
[9]Present address: Division of Interdisciplinary Medical Science, Center for Neuroscience, United Centers for Advanced Research and Translational Medicine, Tohoku University Graduate School of Medicine, Miyagi 980-8575, Japan
*Correspondence: david.digregorio@pasteur.fr (D.A.D.), ttakahas@mail.doshisha.ac.jp (T.T.)
http://dx.doi.org/10.1016/j.neuron.2014.11.019
This is an open access article under the CC BY license (http://creativecommons.org/licenses/by/3.0/).

SUMMARY

Synaptic efficacy and precision are influenced by the coupling of voltage-gated Ca^{2+} channels (VGCCs) to vesicles. But because the topography of VGCCs and their proximity to vesicles is unknown, a quantitative understanding of the determinants of vesicular release at nanometer scale is lacking. To investigate this, we combined freeze-fracture replica immunogold labeling of $Ca_v2.1$ channels, local [Ca^{2+}] imaging, and patch pipette perfusion of EGTA at the calyx of Held. Between postnatal day 7 and 21, VGCCs formed variable sized clusters and vesicular release became less sensitive to EGTA, whereas fixed Ca^{2+} buffer properties remained constant. Experimentally constrained reaction-diffusion simulations suggest that Ca^{2+} sensors for vesicular release are located at the perimeter of VGCC clusters (<30 nm) and predict that VGCC number per cluster determines vesicular release probability without altering release time course. This "perimeter release model" provides a unifying framework accounting for developmental changes in both synaptic efficacy and time course.

INTRODUCTION

Fast and precise chemical synaptic transmission is thought to be achieved through the colocalization of voltage-gated Ca^{2+} channels (VGCCs) and release-ready synaptic vesicles at the presynaptic active zone (AZ) (Eggermann et al., 2012). However, the effect on release of exogenous calcium buffers, such as EGTA, suggests that the "coupling" distance between VGCCs and the Ca^{2+} sensor for vesicular release (VGCC-sensor distance) varies across mammalian synapses producing either "loose" (Rozov et al., 2001; Fedchyshyn and Wang, 2005; Vyleta and Jonas, 2014) or "tight" coupling (Mintz et al., 1995; Fedchyshyn and Wang, 2005; Bucurenciu et al., 2008; Schmidt et al., 2013). Detailed simulations of Ca^{2+} buffering and diffusion indicate that the efficacy and time course of vesicular release can be sensitive to differences in the VGCC-sensor distance as small as 5–10 nm (Bennett et al., 2000; Meinrenken et al., 2002; Bucurenciu et al., 2008; Wang et al., 2009; Scimemi and Diamond, 2012). However, the ability of such simulations to reproduce the amplitude and time course of action potential (AP)-evoked vesicular release is limited, since key model parameters have not been experimentally measured. These parameters include knowledge of the spatial distributions of VGCCs and Ca^{2+} sensors, as well as intracellular Ca^{2+} buffering properties.

Lack of information on the spatial arrangement of VGCCs and synaptic vesicles within the AZ has led to divergent models of synaptic release, ranging from clustered VGCCs with random vesicle placement (Meinrenken et al., 2002; Ermolyuk et al., 2013) to random placement of both VGCCs and vesicles (Scimemi and Diamond, 2012). Recent advances in Ca^{2+} channel antibodies and freeze-fracture replica labeling electron microscopy (EM) have established that VGCCs form clusters at the AZ of central mammalian synapses (Kulik et al., 2004; Bucurenciu et al., 2008; Holderith et al., 2012; Indriati et al., 2013), but the number, density, and distribution of VGCCs within these clusters and their influence on vesicular release have not been explored. Indeed, estimates for the number of VGCCs necessary to drive vesicular release range from 1 (Stanley, 1993), to several (Fedchyshyn and Wang, 2005; Bucurenciu et al., 2010; Scimemi

and Diamond, 2012), or to >10 (Borst and Sakmann, 1996; Nadkarni et al., 2010; Sheng et al., 2012).

To understand how the spatial distribution of VGCCs affect the VGCC-sensor coupling, we studied the calyx of Held synapse, since many of its properties are well characterized, and it is particularly amenable to presynaptic imaging and whole-cell patch pipette perfusion with exogenous buffers. By combining functional measurements, freeze-fracture replica immunogold labeling of $Ca_v2.1$ channels, and experimentally constrained 3D models of Ca^{2+} diffusion and vesicular release, we estimated VGCC-sensor distance at different stage of development. Model predictions were tested against measurements of the sensitivity of vesicular release to EGTA, vesicular release probability, and the time course of release. Our results suggest that the Ca^{2+} sensors for vesicular release are located close to the perimeter of VGCC clusters. Moreover, our findings reconcile apparent inconsistencies across various experimental findings and explain how the speed and efficacy of AP-evoked vesicular release is differentially modulated during development.

RESULTS

Clustering of $Ca_v2.1$ Subunits at the Calyx of Held at Different Developmental Stages

The calyx of Held synapse undergoes both morphological and functional changes during the second postnatal week, when rodents start to hear sounds (Kandler and Friauf, 1993; Taschenberger et al., 2002). To examine whether these functional changes are associated with alterations in the VGCC distribution, we performed SDS-digested freeze-fracture replica labeling (SDS-FRL) (Fujimoto, 1995; Hagiwara et al., 2005) with an antibody specific for the $Ca_v2.1$ subunit of P/Q-type VGCCs (Holderith et al., 2012; Miyazaki et al., 2012; Indriati et al., 2013). Large continuous membranes with abundant intramembrane particles and shallow convex structures were identified as the presynaptic protoplasmic face (P-face) of a calyx when it adjoined the cross-fractured face through the presynaptic terminal containing synaptic vesicles. As expected from the intracellular location of the epitope, immunogold particles (5 nm diameter) for $Ca_v2.1$ were predominantly found on the presynaptic P-face, with little labeling on the exoplasmic face or cross-fractured face (Figure 1A). Specificity of the antibody in replica labeling was confirmed using $Ca_v2.1$ knockout mice, which showed little $Ca_v2.1$ labeling (Figures S1A and S1B available online). Averaging over the entire P-face of calyx presynaptic membrane produced a mean density of immunogold particles of 2.6/μm^2 at P7, 6.7/μm^2 at P14, and 4.6/μm^2 at P21 in one set of labeling samples (La1). In another set of labeling samples using a different batch of antibodies (La2), a higher particle density was observed (8.6/μm^2 at P7 and 21.7/μm^2 at P14).

Because the gold particles appeared to form clusters, we tested this possibility by comparing the gold particle distribution with a randomly distributed particle model (Figures S1C–S1F). To define a cluster, we drew a circle of various radii (50–500 nm) around each particle and compared the "cluster rate" between random and real particle distributions (for details, see Figure S1F legend). When the circles overlapped, particles were assigned to a single cluster (Figures 1A$_3$, S2A, and S2C). We found that the cluster rate of the real particle distribution relative to the random distribution was highest when the radius was 100 nm. We therefore used this radius along with the condition that two or more gold particles are located within the encircled area as our definition of a cluster. Using this criterion, most gold particles (>80%, La1; >97%, La2) were located within these clusters regardless of developmental stage (Figures 1B–1D).

$Ca_v2.1$ labeling followed by RIM staining revealed that the majority of $Ca_v2.1$ clusters were closely associated with RIM particles (64% at P7, 87% at P14, and 74% at P21, Figures S2C and S2D), supporting the idea that $Ca_v2.1$ clusters are localized in AZs. The nearest neighbor distance (NND) between clusters was similar, but not identical, across ages (La1): 899 ± 60 nm for P7, 779 ± 32 nm for P14 ,and 880 ± 38 nm for P21 (Figure 1E). Similar values were observed in La2 for P7 (785 ± 29 nm, n = 44) and P14 (787 ± 41 nm, n = 31). These intercluster NNDs are longer than that estimated for AZs at the P9 calyx (Sätzler et al., 2002) but comparable to those at the P14 calyx (940 nm; calculated from Taschenberger et al., 2002). The similar NNDs for $Ca_v2.1$ clusters and AZs suggest that most AZs contain a single cluster of $Ca_v2.1$ channel.

Quantification of the Spatial Distribution of $Ca_v2.1$ Subunits within Clusters

In order to evaluate the local distribution of $Ca_v2.1$ immunogold particles within clusters, we superimposed 92 VGCC cluster profiles aligned at their center (Figure 2A) and plotted the density function from the cluster center (Figure 2B). Particles at P7 were more spatially confined than those after P14. Cluster area, computed from a perimeter line drawn through the outermost gold particles of a cluster, increased from P7 (0.0020 μm^2, n = 46) to P14 (0.0065 μm^2, n = 146) and then remained constant for P21 (0.0067 μm^2, n = 105, Figure 2C). The $Ca_v2.1$ cluster area at P14 corresponds to 12% of the AZ area estimated previously (Taschenberger et al., 2002).

The number of particles per cluster in La1 samples varied over a wide range, from 2 to 27 (Figure 2D) with an average of 3.1 ± 0.3 at P7 (n = 93 clusters), 5.3 ± 0.8 at P14 (n = 199), and 4.7 ± 0.3 at P21 (n = 167, Figure 2E). In La2, the number was approximately 2-fold larger ranging from 2 to 45 with a mean of 6.4 ± 0.6 at P7 (n = 69) and 11.1 ± 1.2 at P14 (n = 67), a similar age ratio to that in La1. In contrast, the NND between particles within a cluster remained similar from P7 (28 ± 1 nm) to P14 (30 ± 1 nm) in La2. The NND in La1 (39 ± 2 nm for P7, 37 ± 1 nm for P14, and 37 ± 1 nm for P21) was longer than those in La2, as expected for less efficient labeling (Experimental Procedures), and was similar throughout development as for La2 (Figure 2F, $p > 0.8$, Kolmogorov Smirnov test). The distribution of $Ca_v2.1$ NND was narrow, with 80% of the NNDs between 13 and 55 nm (La1). These data indicate that the density of $Ca_v2.1$ remains similar from P7 to P21, but the number of $Ca_v2.1$ per cluster and the cluster area increase with development from P7 to P14.

Presynaptic Ca^{2+} Dynamics Evoked by Single APs at Different Developmental Stages

In order to examine whether the spatio-temporal profile of presynaptic AP-evoked [Ca^{2+}] changes was altered during the hearing acquisition period, we recorded local Ca^{2+} transients in response to single APs using a high-speed confocal spot

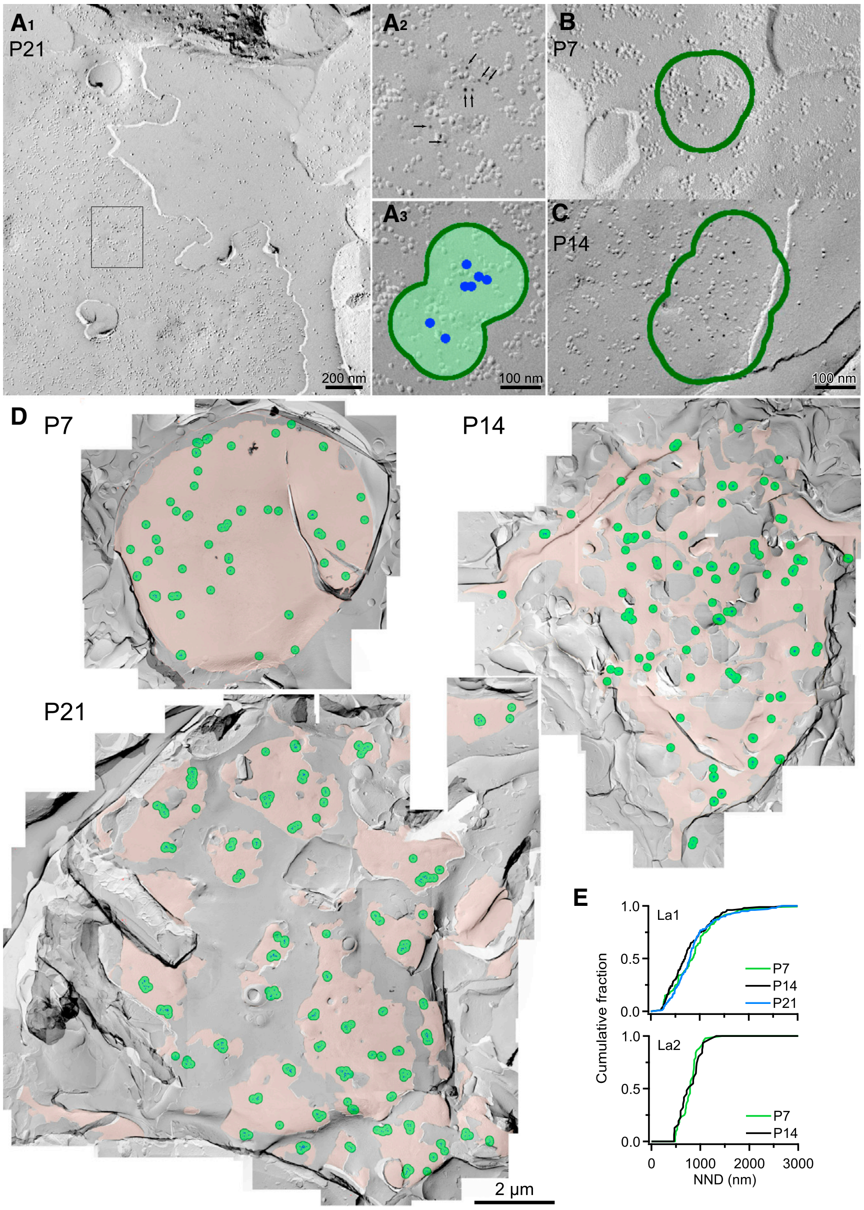

Figure 1. Distribution of $Ca_v2.1$ Immunoparticles at the Developing Calyx of Held Presynaptic Terminal Revealed with SDS-FRL

(A1) Freeze-fracture replica image of a calyx of Held of a P21 rat. Presynaptic P-face classification is confirmed by the presence of convex structures in the cross-fractured face that are likely to reflect synaptic vesicles. Round, electron-dense particles (5 nm diameter) indicate immunogold-labeled $Ca_v2.1$ antibodies, which are often seen in characteristic concaved surface with dimples (Figures S2A and S2B). The higher magnification of this image is shown in Figure S2A.

(A2) Zoom of box region in (A1). Arrows indicate 5 nm immunogold particles.

(A3) Clusters of two or more particles were identified by the overlap of 100 nm radius circles (green) centered on the particles (blue).

(B) Typical immunogold particle cluster at P7.

(C) Typical immunogold particle cluster at P14.

(D) Low magnification of immunogold particle clusters (green) in the presynaptic P-face (pink) of calyces of Held at different ages.

(E) Cumulative histograms of NND between cluster centers comparing different ages in La1 (upper panel, p = 0.03 for P7 versus, P14, p = 0.27 for P7 versus, P21, p = 0.008 for P14 versus, P21) and La2 (lower panel, p = 0.26 for P7 versus, P14). Statistical comparisons were performed using a Kolmogorov-Smirnov test. La1 and La2 indicate samples reacted with different antibody batches resulting in a higher efficiency labeling for La2 (62% versus 19% for La1). All images are taken from La1 samples.

detection method and low-affinity Ca^{2+} indicator Oregon green BAPTA-5N (DiGregorio et al., 1999). The point spread function of the microscope was 220 nm (*XY* axis) and 650 nm (*Z* axis) (Figure S3). In P7 calyces, Ca^{2+} transients were observed in the majority of locations tested on the synaptic face (79% ± 3%, n = 5 calyces, Figure 3A). Ca^{2+} transients rose rapidly during the repolarization phase of the AP, and their decays exhibited both a fast (2–5 ms) and slow (>10 ms) component, consistent with localized detection of a [Ca^{2+}] domain (DiGregorio et al., 1999). In contrast, Ca^{2+} transients were slower and smaller when measured at locations away from the synaptic face (Figures 3B and S4), consistent with the report that VGCC currents were recorded exclusively at the synaptic face (Sheng et al., 2012).

In P14 calyces Ca^{2+} transients were also observed for confocal spot locations along the synaptic face, albeit less frequently than in P7 calyces (44% ± 16%, n = 5 calyces), and exhibited a similar spatial dependence (Figure 3C). To compare the amplitude of Ca^{2+} transients between ages, we selected transients recorded at the synaptic face with rise times less than 0.5 ms. Fast rise times are an indication that the confocal spot is located close (<200 nm) to the Ca^{2+} entry site (Figure S4). The mean peak amplitude of Ca^{2+} transients at P14 (0.17 ± 0.01 ΔF/F, n = 25 spot locationss from six calyces; Figure 3D) was 50% of that at P7 (0.35 ± 0.03 ΔF/F, n = 27 spot locations from eight calyces). To determine whether a developmental decrease of the AP duration underlies the age difference in the Ca^{2+} transient amplitude, we prolonged the AP duration at P14 calyces using 1 to 2 mM tetraethyl ammonium (TEA), thus mimicking the AP measured at P7 (Ishikawa et al., 2003). TEA caused an 83% ± 30% increase in the Ca^{2+} transient amplitude (Figure 3E). In a different set of experiments, we voltage clamped P7 calyces using an AP-waveform voltage command derived from P7 (AP_7) or P14 (AP_{14}) calyces. The shorter duration AP_{14} waveform reduced the time to peak of the Ca^{2+} current (I_{Ca}) by 51% ± 1% (n = 9 calyces, p < 0.01, paired t test) as compared to using AP_7 waveforms. Ca^{2+} transients recorded from the same spot location were 56% smaller when elicited using AP_{14}

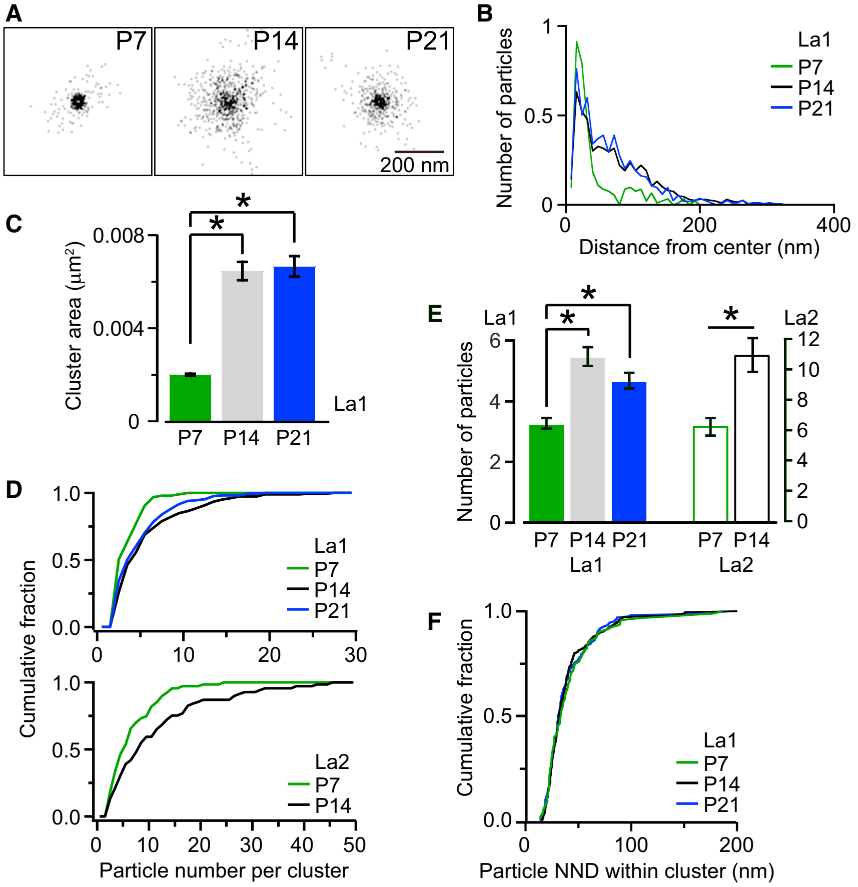

Figure 2. $Ca_V2.1$ Immunogold Particle Distribution within Clusters as a Function of Age

(A) Overlaid gold particle clusters (La1) aligned at their center of gravity (92 clusters per age).

(B) Spatial distribution profiles of immunogold particles showing particle distances from the center of gravity of its corresponding cluster. Ordinate indicates number of particles in concentric bins (8 nm).

(C) Mean cluster area (± SEM) for La1. Only clusters having three or more particles were included in this analysis (*$p < 0.01$, one way ANOVA followed by Tukey's post hoc).

(D) Cumulative histograms of immunogold particle number per cluster from La1 (upper) and La2 (lower).

(E) Mean number (± SEM) of immunogold particles within a cluster for La1 (filled bars, *$p < 0.01$, one-way ANOVA followed by Tukey's post hoc) and La2 (open bars, *$p < 0.01$, t test).

(F) Cumulative histograms of intracluster particle NND values for La1.

than when elicited by AP_7 (Figure 3F). Hence, these results show that the Ca^{2+} influx is restricted to the synaptic face of calyces at both P7 and P14 and that the shortening of the presynaptic AP duration can account for a substantial fraction of the decrease in the Ca^{2+} transient amplitude during development.

Properties of Endogenous Fixed Buffer

The properties of endogenous buffers can influence the spatio-temporal profile of the $[Ca^{2+}]$ that drives vesicular release (Eggermann et al., 2012). We estimated the properties of endogenous fixed buffers (EFBs) in dialyzed terminals (i.e., without endogenous mobile buffers) by altering the concentration of an exogenous mobile buffer (Neher and Augustine, 1992), in this case EGTA, and monitoring its effect on Ca^{2+} transients. EGTA accelerates the decay of spatially equilibrated Ca^{2+} transients (Atluri and Regehr, 1996; Markram et al., 1998) due to its slow binding rate (10^7 $M^{-1}s^{-1}$; Nägerl et al., 2000). Through competition for free Ca^{2+}, we thus expect that the amount of EFB would be inversely proportional to the ability of EGTA to accelerate the decay of the local Ca^{2+} transient. We found no age difference in the decay time course of Ca^{2+} transients recorded at spot locations close to putative Ca^{2+} entry sites (i.e., transients with a rise time <0.5 ms) with either 0.1 mM or 2 mM EGTA-containing pipette solutions (Figures 4A–4C). This result suggests that the concentration and kinetic properties of EFBs do not significantly change between P7 and P14.

To estimate the kinetic properties of EFB, we compared measured Ca^{2+} transients with those predicted from 3D Ca^{2+} reaction-diffusion simulations in which the EFB properties were varied. For these simulations we used experimentally determined parameters, including the microscope point spread function (Figure S3), cytoplasmic diffusion properties of Ca^{2+}, and the Ca^{2+} buffering and diffusion properties of Oregon green BAPTA-5N, ATP and EGTA (Table S1). The on and off binding rates of the EFB, and the number of open VGCCs, were free parameters. For a fixed number of open channels at each age (P7 and P14), a low-affinity EFB with fast on and off rate constants (k_{on}, 1×10^8 $M^{-1}s^{-1}$; k_{off}, 1×10^4 s^{-1}; $K_d = 100$ μM, Xu et al., 1997), and a buffer capacity of $\kappa = 40$ (Helmchen et al., 1997) best matched the experimental measurements obtained with both 0.1 and 2 mM EGTA (Figures 4D and 4E). Moreover, the magnitude of inhibition of the peak Ca^{2+} transient by EGTA in our model (10% for P7 and 9% for P14) matched that of the experimental data (10% for P7 and 11% for P14; Figure S5), suggesting that a low-affinity EFB is present at 4 mM throughout development. When we repeated the simulations with a 50-times higher affinity EFB (k_{on}, 5×10^8 $M^{-1}s^{-1}$; k_{off}, 1×10^3 s^{-1}; $K_d = 2$ μM, Meinrenken et al., 2002) (Figure 4D) or lower κ (Figure 4E), the result matched less well to the experimental data. Interestingly, a low-affinity EFB was also required for simulating $[Ca^{2+}]$ changes induced by uncaging (Bollmann and Sakmann, 2005). These results show that a fast, low-affinity EFB is present in the calyx of Held and that the kinetic properties of this EFB remain constant during development.

Developmental Changes in the Sensitivity of Excitatory Postsynaptic Currents to Presynaptic EGTA

The sensitivity of excitatory postsynaptic currents (EPSCs) to presynaptic EGTA has been used to assay the VGCC-sensor

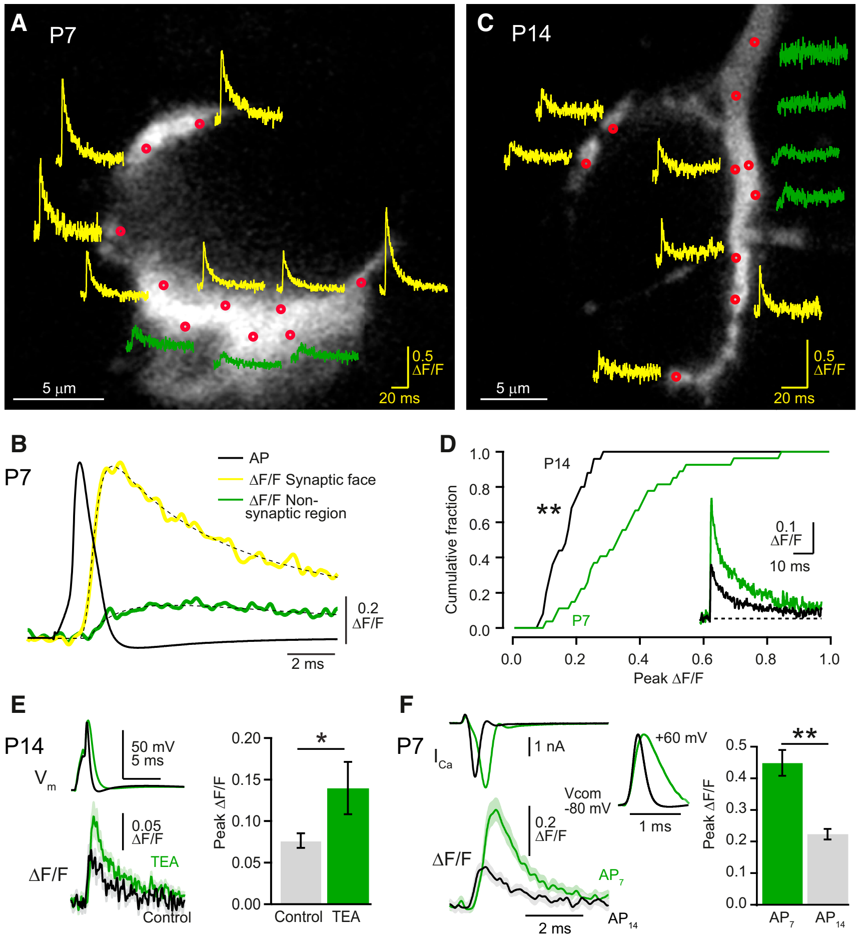

Figure 3. Developmental Reduction of AP-Induced Ca^{2+} Transients Measured with Confocal Spot Detection

(A) Red channel confocal image of a P7 calyx of Held loaded with Alexa 594 and Oregon green BAPTA-5N. Single AP-induced Ca^{2+} transients are presented as ΔF/F and were recorded at various locations within the terminal (red circles) using confocal spot detection. Yellow and green traces show single Ca^{2+} transients recorded from confocal spots on the synaptic face and nonsynaptic regions, respectively.

(B) Temporal relationship between the presynaptic AP (black trace) and Ca^{2+} transients recorded from spot locations along the synaptic face (yellow trace, average of seven locations in [A]) or at nonsynaptic regions (green trace, average of three locations in [A]) in a P7 calyx. Dashed line is a fit with Equation 2 (Supplemental Experimental Procedures).

(C) Same as (A) but for a P14 calyx. Traces are averages of five trials.

(D) Cumulative amplitude histograms of Ca^{2+} transient amplitudes at P7 and P14 (**$p < 0.01$, Kolmogorov-Smirnov test). Inset, the population average at P7 (green, 21 spots from 8 calyces) and P14 (21 spots from 6 calyces).

(E) Left: Averaged AP (V_m) and synaptic-face Ca^{2+} transient in the absence (black; n = 29 traces) or presence (green; n = 21 traces) of 1 to 2 mM TEA (shaded areas denote 2× SEM). Right: Mean peak amplitude (± SEM) of Ca^{2+} transients in control and TEA (P14, n = 7 calyces, *$p < 0.05$, paired t test).

(F) Left: Whole-terminal I_{Ca} and averaged Ca^{2+} transient (n = 16 traces) evoked in voltage clamp using an AP-waveform recorded previously from P7 (green, AP_7, inset) and P14 (black, AP_{14}, inset) calyces. EGTA concentration was 2 mM. Right: Mean peak amplitudes (± SEM) of Ca^{2+} transients for AP_7 and AP_{14} at P7 calyces (**$p < 0.01$, paired t test, n = 9 calyces).

distance at the calyx of Held (Borst and Sakmann, 1996; Fedchyshyn and Wang, 2005). We revisited the EGTA sensitivity of vesicular release using pipette perfusion, since this method allowed us to change only the [EGTA], thereby removing potentially confounding effects of EGTA-independent changes in synaptic efficacy before and after patch rupture (Fedchyshyn and Wang, 2005). Presynaptic pipette perfusion with the same internal solution (0.1 mM EGTA) as used for whole-cell recording from the calyx had no effect on EPSCs, demonstrating that patch pipette perfusion per se did not affect release properties (Figure S6). After recording EPSCs with the control solution, patch pipette perfusion of a new solution containing 10 mM EGTA reduced the EPSC amplitude within several minutes. At P7, the reduction in EPSC amplitude caused by 10 mM EGTA (EGTA-inhibition) was 69% ± 3% (n = 13, Figure 5A). A similar magnitude of EGTA-inhibition (66% ± 3%, n = 5, Figures 5B and 5F) was observed in the presence of a $Ca_v2.2$-specific blocker ω-conotoxin GIVA (CgTX, 2 μM) and a $Ca_v2.3$-specific blocker SNX-482 (SNX, 0.5 μM), suggesting the VGCC-sensor distance is similar among VGCC subtypes. Consistent with these results, we observed no significant difference in the spatial distribution of Ca^{2+} entry estimated with confocal line-scans with and without these subtype-specific VGCC blockers (Figure S7), suggesting that $Ca_v2.2$ and $Ca_v2.3$ have a similar distribution to $Ca_v2.1$ before hearing onset.

Prominent inhibitory effects of 10 mM EGTA on the EPSC amplitude were also observed in older calyces, with an EPSC reduction of 56% ± 5% at P14 (n = 13, Figures 5C and 5F) and 46% ± 6% at P21 (n = 10, Figures 5D and 5F). We also tested the effect of membrane-permeable EGTA-AM on the EPSC amplitude in unperturbed P16–P22 calyces, where endogenous mobile Ca^{2+} buffers remained intact. Bath application of EGTA-AM (10 μM) for 15 min reduced the EPSC amplitude by 35% ± 11% (n = 5, Figure 5E), as previously reported in P14–P18 mice (Fedchyshyn and Wang, 2007). Our results with patch pipette perfusion indicate that 10 mM EGTA has a more potent inhibitory effect on the EPSC amplitude at P14–P21 rat calyces (Figure 5F) than reported previously with patch rupture methods (Fedchyshyn and Wang, 2005). Nevertheless, the magnitude of EGTA-inhibition did decrease between P7 and P21 (Fedchyshyn and Wang, 2005).

Estimating the VGCC-Sensor Distance

Our measurements of the VGCC distribution (Figures 1 and 2); EFB properties (Figure 4); and the time course of AP-induced Ca^{2+} influx (Figure 3F), along with the previously reported

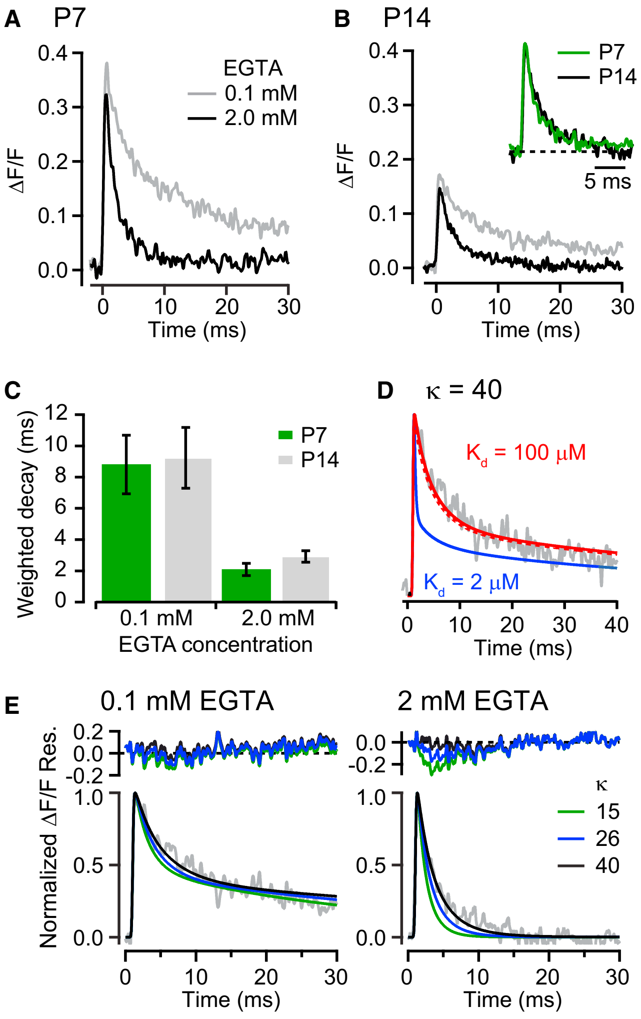

Figure 4. Quantification of Endogenous Fixed Ca^{2+} Buffer Properties at P7 and P14

(A) Averaged Ca^{2+} transient at the synaptic face of P7 calyces in the presence of 0.1 mM (gray, n = 25 calyces) or 2 mM (black, n = 33) EGTA in presynaptic pipettes.

(B) Same as (A) but for P14 calyces (n = 23 and 33 respectively). Inset shows normalized Ca^{2+} transients for 2 mM EGTA at P7 (green) and P14 (black) for kinetic comparison.

(C) Average weighted mean time constant (± SEM) of the Ca^{2+} transients at P7 and P14.

(D) A normalized simulated Ca^{2+} transient computed with a fast EFB k_{off} ($1.0 \times 10^4\ s^{-1}$, $K_d = 100\ \mu M$, red trace) matched the experimental Ca^{2+} transient (gray) better than when computed with a slow EFB k_{off} ($1.0 \times 10^3\ s^{-1}$, $K_d = 2\ \mu M$, blue). The EFB concentration was adjusted to keep binding capacity κ = 40.

(E) Normalized simulated Ca^{2+} transients with a low-affinity EFB ($K_d = 100\ \mu M$) of κ = 15 (green), 26 (blue), and 40 (black) in the presence of 0.1 mM (left) and 2 mM EGTA (right). Normalized experimental Ca^{2+} transients (P14; gray) recorded in 0.1 and 2 mM EGTA are plotted on left and right panels, respectively. Residuals (Res.) were calculated from the difference between the normalized simulation traces and experimental traces.

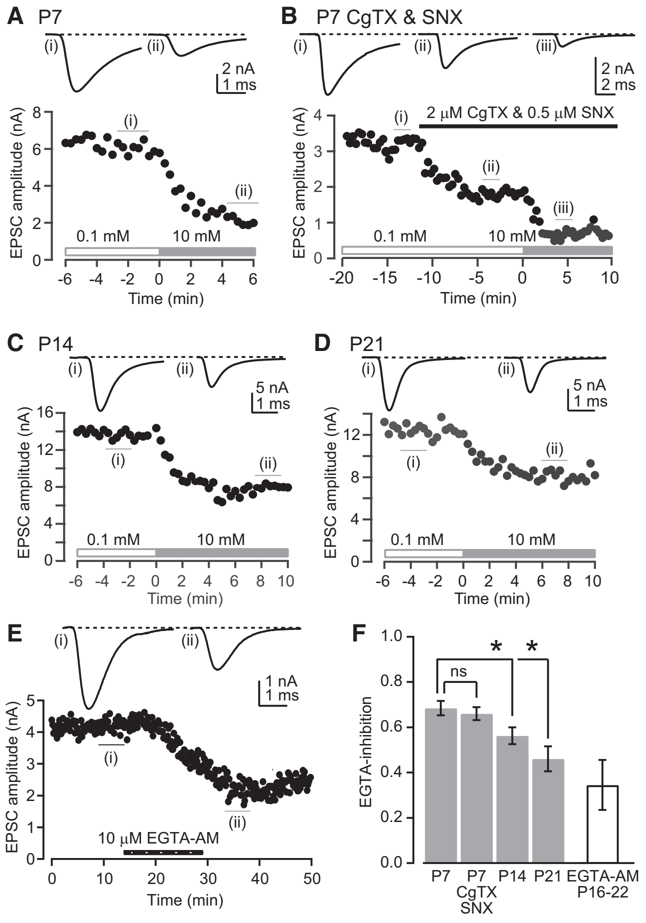

Figure 5. Effects of Intraterminal EGTA Perfusion on the EPSC Amplitude

(A) EPSCs evoked by single presynaptic APs (elicited in current clamp) at P7, before (i) and after (ii) presynaptic internal pipette perfusion of 10 mM EGTA. Time 0 is defined by the time at which 10 mM EGTA was infused (horizontal solid gray bar).

(B) EPSCs evoked from a P7 calyx before (i) and after (ii) bath application of 2 μM ω-conotoxin and 0.5 μM SNX, and then after presynaptic internal pipette perfusion from 0.1 to 10 mM EGTA (in the presence of VGCC blockers, iii). The reduction in EPSC amplitude due to blockers was 46% ± 5% (n = 5 calyces).

(C) Same as (A) but for P14 calyces.

(D) Same as (A) but for P21 calyces.

(E) Effects of extracellular application of EGTA-AM (10 μM) on EPSCs evoked by extracellular fiber stimulation of an unperturbed P18 calyx.

(F) Summary of the EPSC amplitude reduction (EGTA-inhibition; mean ± SEM) due to intraterminal perfusion of 10 mM EGTA (gray bars) at P7–P21 calyces after application of CgTX and SNX ($p = 0.86$, unpaired t test) or extracellular application of EGTA-AM (open bar). $^{*}p < 0.01$; one-way ANOVA followed by Tukey's post hoc.

single-channel conductance of $Ca_v2.1$ (Sheng et al., 2012), exogenous Ca^{2+} buffer kinetics (Nägerl et al., 2000) and Ca^{2+} sensor models (Kochubey et al., 2009), allowed us to construct an experimentally constrained 3D reaction-diffusion model of Ca^{2+} and vesicular release to estimate the VGCC-sensor distance. We modeled P14 calyces by simulating Ca^{2+} entry induced by an AP_{14} waveform using a simple grid array of either 4 or 12 open VGCCs, where the VGCCs were located 35 nm apart (Figure 6A). These two configurations spanned the range of open VGCCs per cluster, which we estimated from La1 samples (gold particle

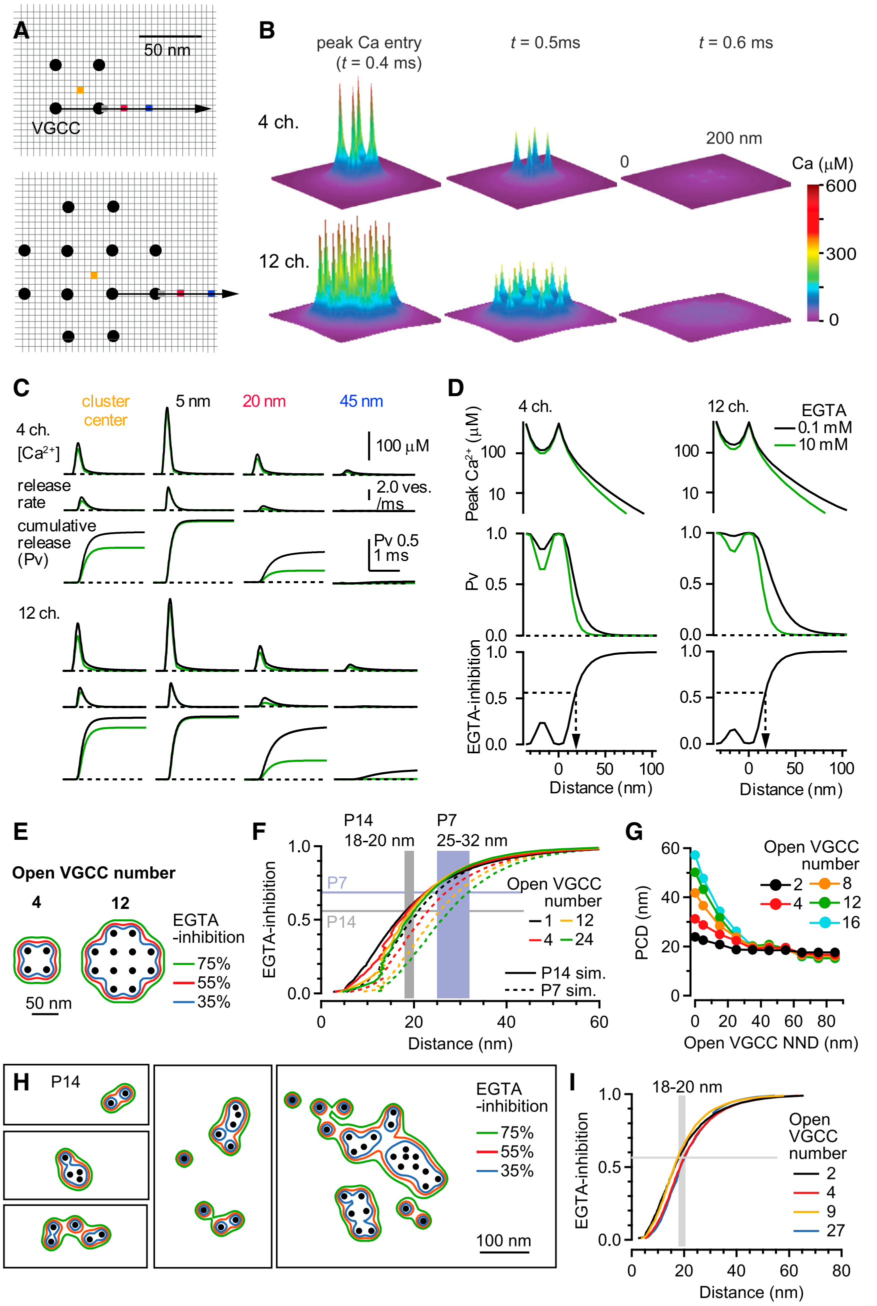

Figure 6. Estimation of the VGCC-Sensor Distance with 3D Reaction-Diffusion Simulations of [Ca^{2+}] and Vesicular Release

(A) Cartoon of the "grid array" of 4 and 12 open VGCCs (top and bottom; circles) on the terminal membrane for a 3D reaction-diffusion simulation. The NND of the VGCCs was 35 nm, and each voxel was 5 × 5 × 5 nm. Colored voxels correspond to location of simulation traces shown in (C). Arrows indicate the location of line profiles in (D).

(B) Spatial distribution of the [Ca^{2+}] in single voxels at the terminal membrane generated by the open VGCC clusters in (A), displayed at the time of peak Ca^{2+} entry, 0.1 ms and 0.2 ms after the peak. We set *t* = 0 at the time of a 50% rise time of the presynaptic AP.

(C) Time course of [Ca^{2+}], vesicular release rate, and cumulative vesicular release probability at colored voxel locations in (A) for control conditions (0.1 mM EGTA, black) and 10 mM EGTA (green).

(D) Spatial profile of [Ca^{2+}], vesicular release probability (P_v), and the fractional reduction of P_v by 10 mM EGTA (EGTA-inhibition). Dashed line and arrow indicates distance at which 55% EGTA-inhibition was observed.

(E) Contour plots for isovalue lines of EGTA-inhibition (35%, 55%, and 75%) around the open VGCC clusters shown in (A).

(F) EGTA-inhibition as a function of distance between the vesicular Ca^{2+} sensor and VGCC cluster perimeter. The AP waveform and release sensor parameters were set for either P7 or P14 calyces. Horizontal lines indicate the average experimental values of EGTA-inhibition of 56% for P14 (gray) and 69% for P7 (blue). The vertical shaded regions indicate the range of distances between the sensor location and nearest open VGCC matching experimental EGTA-inhibition for differing number of VGCC per cluster for P14 (solid color lines) and P7 (dashed color lines) simulations. The locations where experimental EGTA-inhibition was observed, called PCDs, were 18–20 nm for P14 and 25–32 nm for P7.

(G) NND of open VGCCs versus PCD for different number of open VGCCs.

(H) P14 simulations showing EGTA-inhibition isovalues as in (E), but using five representative gold particle clusters (black dots) observed from SDS-FRL EM samples (La1).

(I) EGTA-inhibition as a function of distance for VGCC locations corresponding to real gold particle patterns containing different numbers of open VGCCs. The PCD ranged between 18 and 20 nm.

NND = 37 nm), because the labeling efficiency (0.19) coincides with the channel open probability per AP (0.2; Sheng et al., 2012). In these simulations, we found steep [Ca^{2+}] gradients surrounding each open VGCC that dissipated rapidly (<0.5 ms) following termination of Ca^{2+} entry (Figure 6B). The peak amplitude and time course of the [Ca^{2+}] at the membrane depended on the distance from the nearest VGCCs (Figures 6C and 6D). In the presence of 0.1 mM EGTA, the peak [Ca^{2+}] at 5 nm from the cluster edge was 230 μM for four open VGCCs and 246 μM for twelve open VGCCs, but at 45 nm, this decreased to 16 μM and 22 μM, respectively.

To simulate vesicular release, we drove a five-state Ca^{2+}-dependent vesicular release model (Kochubey et al., 2009) using the simulated [Ca^{2+}] time courses at various elementary simulation volumes (5 nm cubic voxels) across the entire synaptic face (examples shown in Figure 6C). This approach assumed that the Ca^{2+} sensor for vesicular release was within 5 nm of the membrane, which seems likely given its requisite interaction with the core complex formed by synaptic vesicle fusion proteins (Südhof, 2013). Computing vesicular release probability (P_v) at different locations with respect to the VGCC cluster, we found P_v = 1 within those voxels containing a VGCC, 0.8–1 for voxels

within the cluster center, and 0.01 for voxels 55 and 92 nm from the edge of a cluster with 4 and 12 open VGCC, respectively. Increasing the intraterminal [EGTA] from 0.1 to 10 mM inhibited P_v by 20% at the cluster center. Only when Ca^{2+} sensors were positioned outside the cluster (19 nm from the closest VGCC; Figure 6D) did EGTA-inhibition match the experimental results at P14 (56%; Figure 5F). Hence, these simulation results predict that synapses with $P_v > 0.01$ have Ca^{2+} sensors for vesicular release located less than 100 nm from the edge of a VGCC cluster (for less than 12 open VGCCs).

Since our SDS-FRL results showed that VGCCs cluster size varied widely, we systematically explored how the number and spatial distribution of open VGCCs within a cluster affect EGTA-inhibition. To do this, we drew contour plots indicating the different isovalue locations of EGTA-inhibition (Figure 6E). The average distance between isovalue positions of 56% EGTA-inhibition (the experimental P14 value) and the nearest open VGCC was insensitive to the number of open VGCCs per cluster, falling between 18 and 20 nm outside the cluster (Figure 6F). Changes in the open VGCC density had little effect on this distance, provided that the NND between open VGCCs was longer than 30 nm (Figure 6G), consistent with the estimate from our SDS-FRL data (Figure 2F). These results suggest that the Ca^{2+} sensor for vesicular release is located within a short distance from the edge of VGCC clusters. We call this topography the perimeter release model and refer to the distance between the closest open VGCC and the Ca^{2+} sensor as the perimeter coupling distance (PCD). Simulations using an AP_7 waveform and a Ca^{2+} sensor with higher Ca^{2+} affinity for P8–P10 rats (Kochubey et al., 2009) resulted in PCDs = 25–32 nm for the experimentally measured EGTA-inhibition of 69% (Figure 5F), depending on the number of open VGCCs (1-24, Figure 6F). Since real VGCC clusters exhibit irregular shapes (Figures 1 and S2), we repeated reaction-diffusion simulations using our measured La1 immunogold particle distributions for P14 calyces (Figure 6H). Results showed that the PCD for 56% EGTA-inhibition was similar to that of the above grid array models (18–20 nm) and remained insensitive to the number of open VGCCs per cluster (Figure 6I). Thus, the perimeter release model predicts a developmental shortening of the PCD from ~30 nm at P7 to ~20 nm at P14.

Our perimeter release model is based on [Ca^{2+}] and EPSC measurements from dialyzed terminals, where mobile Ca^{2+} buffers are washed out and partially replaced by 0.1 mM EGTA. Because expression of the mobile buffer calretinin increases with development at calyces of Held (Felmy and Schneggenburger, 2004), we evaluated its impact on P_v and PCD. Simulations including 0.5 mM or 1.2 mM calretinin reduced P_v by 12% and 24%, respectively (Figure S8A), but had no effect on the PCD estimate (20 nm at P14; data not shown). Thus, our model is relatively insensitive to the presence of calretinin even at high concentrations.

The single-channel current amplitude used here for AP_{14} waveform (0.35 pA) is based on single-channel conductance (3.3 pS in 2 mM [Ca^{2+}]) measured for $Ca_v2.1$ at the calyx of Held (Sheng et al., 2012). This channel conductance is similar to that of the $Ca_v2.2$ (2.7 pS) at an autonomic preganglionic synapse (Weber et al., 2010). However, because the single-channel current amplitude can influence the coupling distance (Weber et al., 2010), we examined the model sensitivity to variations in the single-channel current. For single-channel currents greater than or equal to 0.2 pA, the 56% EGTA-inhibition would be outside the cluster (Figure S8B), supporting the perimeter release model.

Estimating the Vesicular Release Probability in the Vicinity of a VGCC Cluster

We next examined whether trial-to-trial variability in the pattern of open VGCCs altered our estimate of the PCD. To do this, we generated 50 different patterns of open VGCCs using the VGCC open probability during an AP, calculated from single-channel recordings (Sheng et al., 2012) (see Supplemental Experimental Procedures). The fraction of sensors in the release state (release fraction) was calculated at each surface-membrane voxel, for each pattern of open VGCCs (Figure 7A, gray traces). As for the deterministic simulations (Figure 6), the release fraction was equal to 1 within voxels where a VGCC opened and then dropped steeply within the cluster at locations where channels did not open (Figure 7A). P_v was calculated by averaging the release fraction across trials. At the center of the cluster composed of 16 VGCCs the peak P_v was 0.45. P_v then decreased with distance outside the cluster (Figure 7B, bottom). These simulations predicted the experimentally observed level of EGTA-inhibition at shorter PCDs than those with fixed VGCC distributions, ranging from 11 to 19 nm for P14 and 19 to 26 nm for P7 (Figures 7B and 7C). The PCD was weakly dependent on the number of VGCCs per cluster (Figure 7D). In contrast, P_v was strongly dependent on the number of VGCCs per cluster (Figure 7E). Moreover, these simulations predict that an average of 20–30 VGCCs per cluster underlie the observed P_v at P7 and P14. Thus, our perimeter release model predicts that the number of VGCCs per cluster has a minor effect on the PCD but is an important determinant of P_v.

Contributions of AP Duration and PCD to Changes in Synaptic Delay and Release Duration during Development

During the period of hearing acquisition, the synaptic delay between the presynaptic AP and the EPSC becomes shorter, and the time course of vesicular release becomes faster at the calyx of Held (Taschenberger and von Gersdorff, 2000; Taschenberger et al., 2005). Numerical simulations suggest that these developmental changes might be mediated by alterations in the VGCC-sensor distance (Bucurenciu et al., 2008), but other findings argue against this hypothesis (Meinrenken et al., 2002). We re-examined this issue by measuring the synaptic delay and time course of vesicular release during EGTA dialysis. Our results show that internal perfusion of 10 mM EGTA had no effect on the synaptic delay at any age investigated, whereas the synaptic delay became shorter between P7 to P21 (Figures 8A and 8B). Moreover, internal perfusion of 10 mM EGTA produced only a modest reduction in the time course of vesicular release at P14 and P21 (~10%), and no change at P7, whereas the half duration of release was reduced by 29% from P7 to P14 (Figure 8C), as previously reported (Taschenberger et al., 2005).

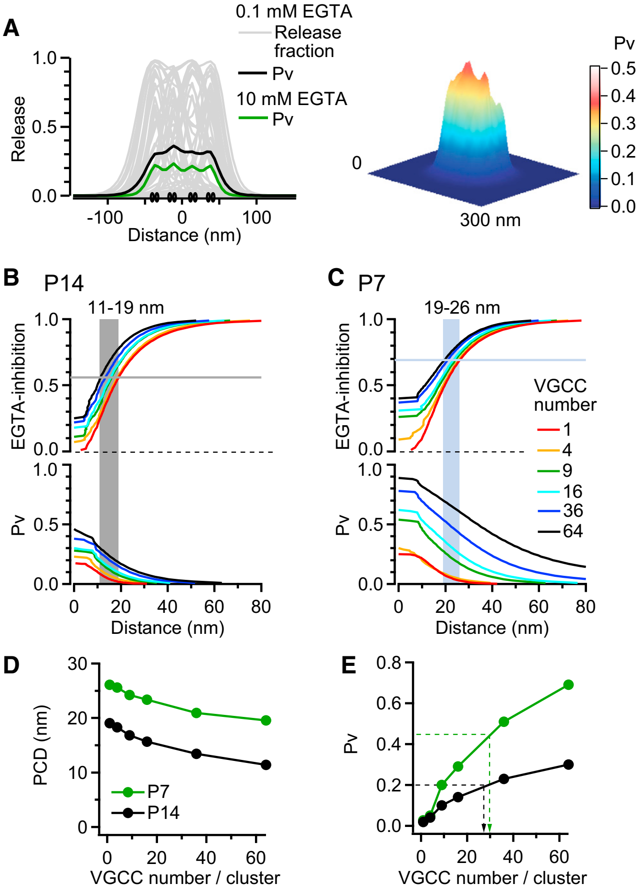

Figure 7. Estimation of the PCD and Vesicular Release Probability in a Model with Randomly Generated Patterns of VGCC Openings

(A) Left: Spatial line profiles (50 trials, gray lines) showing the fraction of sensors in the release state (release fraction) for voxels at the membrane. Release fraction of each trial was simulated from a randomly generated open channel pattern with open probability of 0.175 and a grid arrangement of 16 VGCCs with NND = 20 nm and [EGTA] = 0.1 mM. Black trace is the average across all trials and thus represents P_v. Green trace is the trial average for [EGTA] = 10 mM. Pairs of solid black ovals denote the VGCC locations. Right: 2D plot of the average P_v (0.1 mM EGTA).

(B) EGTA-inhibition and P_v predicted for different sensor locations and numbers of channels using a P14 model (AP_{14} and P14 Ca^{2+} sensor model). Horizontal line indicates the mean EGTA-inhibition of P_v observed in experiments. Gray region indicates PCD range for different number of VGCCs per cluster.

(C) Same as (B) but for a P7 simulation (AP_7 and P7 Ca^{2+} sensor).

(D) PCD plotted against the total number of VGCCs within each cluster.

(E) P_v for AP_7 (green) or AP_{14} (black) plotted against the numbers of VGCCs within each cluster. P7 and P14 Ca^{2+} sensors were used, respectively. Arrows indicate predicted total number of VGCCs per cluster: 29 and 26 for P7 and P14, respectively.

We next examined whether our perimeter release model predicted the observed changes in synaptic delay and time course of vesicular release at different postnatal ages. Simulations for postnatal day 7 (P7) and P14 were performed using AP_7 and AP_{14} waveforms and previously reported sensitivities of the Ca^{2+} sensors for each age (Kochubey et al., 2009). We used a fixed pattern cluster containing six open VGCCs for P7 and four for P14, corresponding to the channel open probability per AP multiplied by the total number of channels per cluster estimated from EM (see Discussion for more details). Simulation results predicted that the vesicular release duration and synaptic delay are steeply influenced by PCD (Figure 8D). Surprisingly, the different AP waveforms between P7 and P14 had a relatively minor effect on the vesicular release duration, whereas they had a marked influence on the synaptic delay (Figure 8E). In contrast, shortening of PCD from 30 nm (for P7) to 20 nm (for P14, see Figure 6) predicted a 31% decrease in the vesicular release half duration (Figure 8D, red arrow) comparable to mean experimental value (28%, Figure 8C). The shortening of synaptic delay (35%, Figure 8B) can be achieved through both changes in AP duration and PCD in simulation, which predicts 40% reduction (Figure 8E, red arrow). The overall release time course for P7 and P14 simulations (in 0.1 and 10 mM EGTA) was comparable to the experimental findings (compare Figures 8F and 8A). Moreover, increasing [EGTA] from 0.1 to 10 mM in the simulations had little effect on the synaptic delay and only a small reduction in the vesicular release half duration (Figure 8G), similar to our experimental findings (Figures 8B and 8C).

Neither the number of open VGCCs (Figure S8C) nor the sensitivity of the Ca^{2+} sensor (Figure S8D) affected the synaptic delay or release time course. A fast, low-affinity EFB resulted in a larger change in the release time course for increasing VGCC-sensor distance (Figures S8E and S8F), unlike model predictions using a high-affinity EFB (Meinrenken et al., 2002). Thus, a low-affinity EFB is critical for predicting the developmental speeding of the release time course. These simulations suggest that developmental shortening of the PCD from 30 to 20 nm is the main determinant for the developmental acceleration of the vesicular release time course (Figure 8D). In contrast, shortening of the PCD accounted for the developmental reduction in synaptic delay only partially (Figure 8E), with the remaining changes caused by the shortening of the AP duration. In simulations using stochastic patterns of open VGCC (Figure 7), the shortening of PCD from 25 to 15 nm also reproduced the developmental changes synaptic delay and release time course (Figure S8G). Thus, the shortening of PCD contributes to the developmental acquisition of synaptic precision that is critical for sound localization at the calyx of Held (Oertel, 1999).

DISCUSSION

We investigated the mechanisms that underlie the speed and precision of vesicular release at the rat calyx of Held, a central excitatory synapse that has been particularly well characterized across different developmental stages. We measured the properties of two key presynaptic parameters that have not previously been quantified: the distribution of VGCCs and the binding kinetics of endogenous fixed Ca^{2+} buffers. Moreover, we re-examined the EGTA sensitivity of neurotransmitter release under more controlled conditions than has been previously achieved. These results were then combined with other known experimental parameters to constrain 3D reaction-diffusion

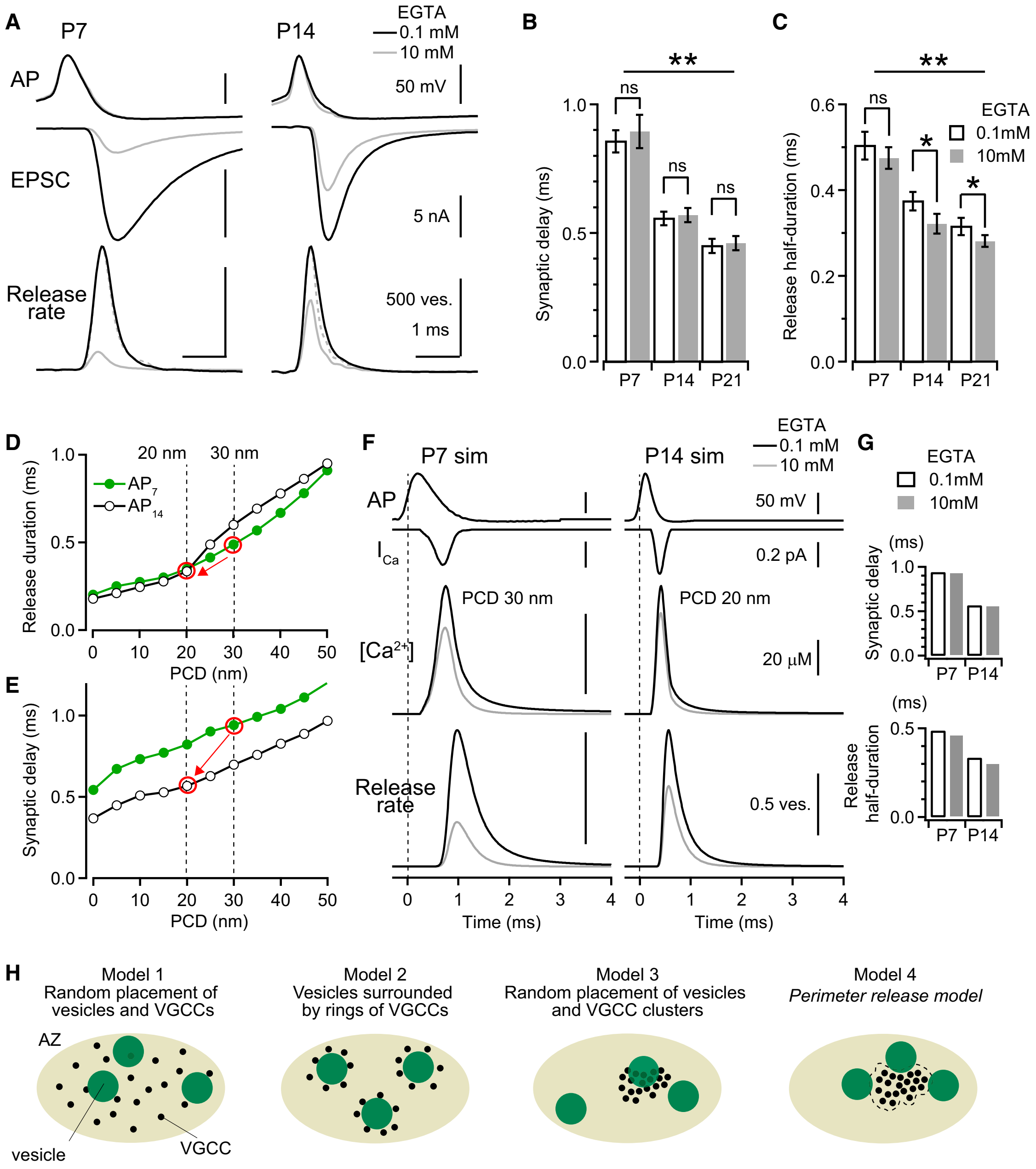

Figure 8. Developmental Changes in Vesicular Release Time Course Are Predicted by Perimeter Release Model

(A) Representative examples of experimentally measured presynaptic APs, EPSCs, and vesicular release rates before (black) and after (gray) presynaptic perfusion of 10 mM EGTA in P7 and P14 calyces. Dashed lines indicate peak scaked release rate in 10 mM EGTA.

(B) Mean synaptic delay (from the 50% rise time of the APs to the 20% rise time of the EPSCs, ± SEM) in the presence of 0.1 mM (open bars) or 10 mM EGTA (filled bars) in the presynaptic pipette solution (n = 10 calyces for P7, n = 8 for P14 and P21).

(C) Release half duration (the width at half maximal of the release rate, ± SEM) in the presence of 0.1 mM and 10 mM EGTA, estimated by deconvolution at P7, P14, and P21 (**$p < 0.01$, one-way ANOVA). Internal perfusion of 10 mM EGTA reduced the release duration by ~15% in P14 and P21 (**$p < 0.05$, paired t test), but not in P7 calyces.

(D) Dependence of release duration on the PCD for AP_7 (green) and AP_{14} (black) waveforms, simulated with the perimeter release model. Red circles indicate values predicted by experimental results, and an arrow indicates the direction of developmental change.

(E) Same as (D) but for synaptic delay.

(F) Temporally aligned simulated traces of the $[Ca^{2+}]$ and vesicular release rate for [EGTA] = 0.1 mM (black) and 10 mM (gray). For P7 and P14 simulations, the timing and duration of Ca^{2+} entry, number of open VGCCs, Ca^{2+} sensor affinity, and PCD for were adjusted specifically for each age.

(G) The simulated effect of 10 mM EGTA on synaptic delay and vesicular release duration for the perimeter release model.

(legend continued on next page)

simulations to examine how different topographical arrangements of VGCCs and vesicles affect neurotransmitter release and to estimate the VGCC-sensor distance at different developmental stages. Our experimental results and simulations suggest a model in which release-ready vesicles are located within tens of nanometers of the outer perimeter of the VGCC clusters. This perimeter release model predicts the properties of vesicular release at the calyx of Held at different developmental stages. Our model provides a new framework for understanding mechanisms that determine high-fidelity transmission at central synapses.

The Number of VGCCs that Contribute to the Release of a Vesicle

Whether vesicular release requires opening of one (Stanley, 1993) or multiple VGCCs (Borst and Sakmann, 1996; Fedchyshyn and Wang, 2005; Bucurenciu et al., 2010; Nadkarni et al., 2010; Scimemi and Diamond, 2012; Sheng et al., 2012) is controversial. The SDS-FRL results reported here show that $Ca_v2.1$ channels exist primarily in clusters. Our experimentally constrained model indicates that a single VGCC close to a vesicle can induce vesicle fusion, albeit with a low P_v (0.02 to 0.03, depending on VGCC open probability). This finding could account for spontaneous EPSCs (Ermolyuk et al., 2013) but not AP-evoked release probability estimated for the whole calyx (0.45 at P7 and 0.19 at P14; Koike-Tani et al., 2008). Our results therefore suggest that at the majority of release sites multiple VGCCs contribute to the release of each vesicle following an AP at the calyx of Held.

In order to estimate the total number of VGCCs per cluster from our immunogold particle distributions, we first calculated the labeling efficiency using whole-terminal Ca^{2+} current measurements (see Supplemental Experimental Procedures). To minimize errors, we further analyzed the higher efficiency samples (La2). At P14, the number of $Ca_v2.1$ channels per cluster was 18 (=11.1/0.62) on average and varied between 3 and 73 across clusters. At P7 we estimate that there are 10 $Ca_v2.1$ channels per cluster, but because $Ca_v2.1$ channels comprise 53% of the VGCCs at P7 (Figure S7), the total number of VGCCs on average is 19. We used a 100 nm radius circle for cluster analysis because it optimally detected real clusters (Figure S1F). We cannot rule out, however, the possibility that some clusters are composed of smaller, closely spaced subclusters (e.g., Figure S2C1). Using numerical simulations of release from stochastic open channel patterns based on the open probability of 0.25 at P7 (Sheng et al., 2012) and 0.175 at P14 (see calculation in Supplemental Experimental Procedures), we predict that 29 (at P7) and 26 (at P14) VGCCs are required (Figure 7E) to reproduce the mean P_v of whole terminal (Koike-Tani et al., 2008). The close match between the anatomical and functional estimates of the number of VGCCs per cluster indicates that physiological vesicular release at the calyx of Held is driven by channel clusters with an average of 20–30 VGCCs.

A Unifying Model for Understanding AP-Evoked Vesicular Release at Central Synapses

Our experiments and simulations suggest a topographical arrangement where most releasable vesicles (in response to a single AP) are located 15–30 nm from the outer perimeter of VGCC clusters (Figure 8H, model 4). However, several other VGCC-sensor topographies have been proposed at mammalian central synapses, including random distributions of both VGCCs and release-ready synaptic vesicles within the AZ (model 1), synaptic vesicles surrounded by a ring of VGCCs (model 2), clusters of VGCCs, and a random distribution of synaptic vesicles within the AZ (model 3, Meinrenken et al., 2002; Schneggenburger and Neher, 2005; Wang et al., 2009; Scimemi and Diamond, 2012; Ermolyuk et al., 2013). SDS-FRL at the calyx of Held indicates that $Ca_v2.1$ channels are clustered at high densities on the synaptic face in areas smaller than AZs (Figures 1 and 2). These observations are compatible with both model 3 and model 4. However, simulations of model 3 (Figure S8H) did not replicate our experimental levels of EGTA inhibition of vesicular release (Figure 5), unless a high number of VGGCs were placed within each cluster (>50 for P14). Because we estimate that 90% of the VGCC clusters contain less than 50 VGCCs (Figure 2D, after labeling efficiency correction), our experimental findings are not consistent with model 3. Moreover, EGTA inhibition and P_v were both predicted by the perimeter release model 4 when the Ca^{2+} sensor was positioned within tens of nanometers from the edge of the VGCC cluster. Although we cannot rule out that vesicles with negligible P_v are located further away, our perimeter release model is consistent with the majority of experimental results on AP-evoked vesicular release at the calyx of Held.

Since $Ca_v2.1$ channels also form clusters within AZs at hippocampal (Holderith et al., 2012) and cerebellar synapses (Indriati et al., 2013), the latter of which has been suggested to have a coupling distance of ~20 nm (Schmidt et al., 2013), our perimeter release model may also be applicable to bouton-type synapses. A recent study at hippocampal mossy-fiber synapses suggests that vesicular release is driven by loose VGCC-sensor coupling (65 nm; see Figure 2C in Vyleta and Jonas, 2014). Using their 90% reduction of EPSCs by 10 mM EGTA, our perimeter release model predicts a similar coupling distance (four open VGCCs, PCD = 40 nm, or 55 nm from center; see Figure 6D) and a slower vesicular release time course for the mossy-fiber terminal (see Figure 8D). However, it remains to be determined if VGCCs also cluster at that synapse. Finally, a similar model has been proposed at invertebrate synapses, where fast phasic release requires a coupling distance of <20 nm (Pan and Zucker, 2009). These findings suggests that short coupling distances (<100 nm) are a general requirement for fast AP-evoked vesicular release. Thus, within the framework of the perimeter release model it is possible to understand many aspects of vesicular release across a wide range of synapse types.

(H) Cartoons showing possible AZ topographies for VGCCs and synaptic vesicles released by a single AP. Model 1, random placement of vesicles and VGCCs within AZ. Model 2, vesicles surrounded by rings of VGCCs. Model 3, random placement of vesicles and VGCC clusters, including within vesicle clusters. Model 4, perimeter release model, where releasable synaptic vesicles are positioned at the perimeter of a VGCC cluster. Whether there are more than one releasable vesicle is only speculative.

Molecular Implication of the Perimeter Release Model

What mechanism might hold vesicles near the perimeter of VGCC clusters? The low-affinity vesicular Ca^{2+} sensor synaptotagmin can directly interact with VGCCs at their intracellular loops at high [Ca^{2+}] (Chapman and Davis, 1998; Watanabe et al., 2010). Overexpression of mutated synaptotagmin1 in the calyx of Held reduces the pool size of release-ready vesicles, increases synaptic delay, and decreases the vesicular release rate, suggesting a role of synaptotagmin in positional vesicle priming (Young and Neher, 2009). The vesicular Rab (3/27)-binding protein RIM1a binds to VGCCs via their β subunits (Kiyonaka et al., 2007). Genetic deletion of RIM1 and RIM2 reduces the VGCC density and the number of docked vesicles at calyces of Held (Han et al., 2011). Thus, synaptotagmins and RIMs may also tether vesicles close to VGCCs. Munc13-1 has also been suggested to mediate the tethering of synaptic vesicles to VGCCs at the calyx of Held (Chen et al., 2013). In contrast, Septin 5 has been proposed to prevent vesicles from approaching VGCCs too closely, and its developmental downregulation is postulated to underlie developmental shorting of the VGCC-sensor coupling distance (Yang et al., 2010). Thus, a number of AZ proteins could be involved in tethering synaptic vesicles close to the perimeter of VGCC clusters (Hallermann and Silver, 2013) and orchestrate the developmental shortening of the VGCC-sensor distance at the perimeter of VGCC clusters.

Functional Implications of the Perimeter Release Model

Restricting vesicular release to the perimeter of VGCC clusters has several important advantages. First, this topography will minimize disruption of VGCC clusters during vesicular fusion with the plasma membrane (Mercer et al., 2011). Second, the number of VGCC in a cluster can regulate P_v without altering the time course of vesicular release (Figures 7B and 7C). Moreover, the heterogeneity of P_v across AZs (Sakaba and Neher, 2001) could be explained by different numbers of VGCCs per cluster, rather than by variable VGCC-sensor distances (Meinrenken et al., 2002). Third, this topography could potentially allow multiple vesicles to be primed at an AZ, enabling multivesicular release with high precision at the calyx of Held (Taschenberger et al., 2002; Budisantoso et al., 2013). Hence, localizing vesicular release close to the perimeter of VGCC clusters ensures the synaptic precision is maintained over a wide range of synaptic strength.

EXPERIMENTAL PROCEDURES

All experiments were conducted in accordance with the guidelines of Doshisha University, Institut Pasteur, the National Institute for Physiological Sciences, and Institute of Science and Technology Austria.

Electron Microscopy and Analysis of SDS-Digested Freeze-Fracture Replica Labeling

P7–P21 rats and mice were perfused with fixative, and brain slices were frozen, fractured, and replicated as described previously (Indriati et al., 2013). Replicas were incubated with an antibody against $Ca_v2.1$ subunit of Ca^{2+} channel (8.1 μg/ml; Miyazaki et al., 2012) overnight followed by incubation with 5 nm gold-conjugated secondary antibody at 15°C. Identification of 5 nm particles on the replicas was based on their size, round shape, and electron density. Platinum coating of intramembrane particles often produced dark shadows that were easily distinguished from gold particles and thus excluded from analysis. Labeling efficiency of $Ca_v2.1$ was estimated by comparing the overall density of the $Ca_v2.1$ immunogold particles with the whole-terminal I_{Ca} at P14. The labeling efficiencies of La1 and La2 were 19% and 62%, respectively. We assumed the same labeling efficiency across ages.

Slice Electrophysiology and Ca^{2+} Imaging

Whole-cell patch-clamp recordings were made from calyces of Held and MNTB neurons of acute brainstem slices prepared from P7–P22 Wistar rats. Presynaptic AP, I_{Ca}, and EPSCs were recorded with a Multiclamp-700 (A or B) amplifier (Molecular Devices). Presynaptic internal solution exchange was performed using pipette perfusion, as described previously (Takahashi et al., 2012). Confocal laser scanning and spot detection of fluorescence was performed using an Ultima scanning head (Prairie Technologies) mounted on an Olympus BX61W1 microscope and equipped with a 60×(1.1 NA) water immersion objective. We monitored intracellular [Ca^{2+}] changes with Oregon green BAPTA-5N added to presynaptic pipette solution. All experiments were performed at room temperature (22°C–24°C). Data analysis was performed with IgorPro 6.3 (WaveMetrics) using NeuroMatic. Deconvolution of EPSCs was performed as describe previously (Sakaba and Neher, 2001; Taschenberger et al., 2005). All values in the text and figures are given as means ± SEM unless otherwise indicated.

Numerical Simulations Ca^{2+} Reaction-Diffusion and Vesicular Release

Ca^{2+} diffusion and binding with Oregon green BAPTA-5N and buffers in the vicinity of VGCC clusters was simulated using a finite-difference method (DiGregorio et al., 1999; DiGregorio et al., 2007) in order to predict spot-detected Ca^{2+} transients. Nanoscale simulations were performed without the Ca^{2+} indicator and then used to drive a five-state Ca^{2+}-dependent release model (Kochubey et al., 2009). All simulation parameters are listed in Tables S1 and S2.

SUPPLEMENTAL INFORMATION

Supplemental Information includes eight figures, two tables, and Supplemental Experimental Procedures and can be found with this article online at http://dx.doi.org/10.1016/j.neuron.2014.11.019.

ACKNOWLEDGMENTS

We thank Taro Ishikawa, Shinichi Iwasaki, Masahiro Kimura, Florian Mueller, and Tetsuhiro Tsujimoto for their contributions during the early stage of this study. We also thank Tetsuya Hori for helping with the pipette perfusion experiments, Hee-Sup Shin for providing $Ca_v2.1$ knockout mice, Masahiko Watanabe for providing $Ca_v2.1$ antibodies, and Steven Aird for editing the paper. This work was supported by the Core Research for Evolutional Science and Technology (CREST) of Japan Science and Technology Agency to T.T. and R.S.; by the funding provided by Okinawa Institute of Science and Technology (OIST) to T.T. and Y.N.; by JSPS Core-to-Core Program, A. Advanced Networks to T.T.; by the Grant-in-Aid for Young Scientists from the Japanese Ministry of Education, Culture, Sports, Science and Technology (#23700474) to Y.N.; by the Centre National de la Recherche Scientifique through the Actions Thematiques et Initatives sur Programme, Fondation Fyssen, Fondation pour la Recherche Medicale, Federation pour la Recherche sur le Cerveau, Agence Nationale de la Recherche (ANR-2007-Neuro-008-01 and ANR-2010-BLAN-1411-01) to D.D. and Y.N.; and by the European Commission Coordination Action ENINET (LSHM-CT-2005-19063) to D.D. and R.A.S. R.A.S. and J.S.R. were funded by Wellcome Trust Senior (064413) and Principal (095667) Research Fellowship and an ERC advance grant (294667) to RAS.

The authors declare no competing financial interests.

Accepted: November 14, 2014
Published: December 18, 2014

REFERENCES

Atluri, P.P., and Regehr, W.G. (1996). Determinants of the time course of facilitation at the granule cell to Purkinje cell synapse. J. Neurosci. *16*, 5661–5671.

Bennett, M.R., Farnell, L., and Gibson, W.G. (2000). The probability of quantal secretion within an array of calcium channels of an active zone. Biophys. J. *78*, 2222–2240.

Bollmann, J.H., and Sakmann, B. (2005). Control of synaptic strength and timing by the release-site Ca^{2+} signal. Nat. Neurosci. *8*, 426–434.

Borst, J.G., and Sakmann, B. (1996). Calcium influx and transmitter release in a fast CNS synapse. Nature *383*, 431–434.

Bucurenciu, I., Kulik, A., Schwaller, B., Frotscher, M., and Jonas, P. (2008). Nanodomain coupling between Ca^{2+} channels and Ca^{2+} sensors promotes fast and efficient transmitter release at a cortical GABAergic synapse. Neuron *57*, 536–545.

Bucurenciu, I., Bischofberger, J., and Jonas, P. (2010). A small number of open Ca^{2+} channels trigger transmitter release at a central GABAergic synapse. Nat. Neurosci. *13*, 19–21.

Budisantoso, T., Harada, H., Kamasawa, N., Fukazawa, Y., Shigemoto, R., and Matsui, K. (2013). Evaluation of glutamate concentration transient in the synaptic cleft of the rat calyx of Held. J. Physiol. *591*, 219–239.

Chapman, E.R., and Davis, A.F. (1998). Direct interaction of a Ca^{2+}-binding loop of synaptotagmin with lipid bilayers. J. Biol. Chem. *273*, 13995–14001.

Chen, Z., Cooper, B., Kalla, S., Varoqueaux, F., and Young, S.M., Jr. (2013). The Munc13 proteins differentially regulate readily releasable pool dynamics and calcium-dependent recovery at a central synapse. J. Neurosci. *33*, 8336–8351.

DiGregorio, D.A., Peskoff, A., and Vergara, J.L. (1999). Measurement of action potential-induced presynaptic calcium domains at a cultured neuromuscular junction. J. Neurosci. *19*, 7846–7859.

DiGregorio, D.A., Rothman, J.S., Nielsen, T.A., and Silver, R.A. (2007). Desensitization properties of AMPA receptors at the cerebellar mossy fiber granule cell synapse. J. Neurosci. *27*, 8344–8357.

Eggermann, E., Bucurenciu, I., Goswami, S.P., and Jonas, P. (2012). Nanodomain coupling between Ca^{2+} channels and sensors of exocytosis at fast mammalian synapses. Nat. Rev. Neurosci. *13*, 7–21.

Ermolyuk, Y.S., Alder, F.G., Surges, R., Pavlov, I.Y., Timofeeva, Y., Kullmann, D.M., and Volynski, K.E. (2013). Differential triggering of spontaneous glutamate release by P/Q-, N- and R-type Ca^{2+} channels. Nat. Neurosci. *16*, 1754–1763.

Fedchyshyn, M.J., and Wang, L.Y. (2005). Developmental transformation of the release modality at the calyx of Held synapse. J. Neurosci. *25*, 4131–4140.

Fedchyshyn, M.J., and Wang, L.Y. (2007). Activity-dependent changes in temporal components of neurotransmission at the juvenile mouse calyx of Held synapse. J. Physiol. *581*, 581–602.

Felmy, F., and Schneggenburger, R. (2004). Developmental expression of the Ca^{2+}-binding proteins calretinin and parvalbumin at the calyx of held of rats and mice. Eur. J. Neurosci. *20*, 1473–1482.

Fujimoto, K. (1995). Freeze-fracture replica electron microscopy combined with SDS digestion for cytochemical labeling of integral membrane proteins. Application to the immunogold labeling of intercellular junctional complexes. J. Cell Sci. *108*, 3443–3449.

Hagiwara, A., Fukazawa, Y., Deguchi-Tawarada, M., Ohtsuka, T., and Shigemoto, R. (2005). Differential distribution of release-related proteins in the hippocampal CA3 area as revealed by freeze-fracture replica labeling. J. Comp. Neurol. *489*, 195–216.

Hallermann, S., and Silver, R.A. (2013). Sustaining rapid vesicular release at active zones: potential roles for vesicle tethering. Trends Neurosci. *36*, 185–194.

Han, Y., Kaeser, P.S., Südhof, T.C., and Schneggenburger, R. (2011). RIM determines Ca^2+ channel density and vesicle docking at the presynaptic active zone. Neuron *69*, 304–316.

Helmchen, F., Borst, J.G., and Sakmann, B. (1997). Calcium dynamics associated with a single action potential in a CNS presynaptic terminal. Biophys. J. *72*, 1458–1471.

Holderith, N., Lorincz, A., Katona, G., Rózsa, B., Kulik, A., Watanabe, M., and Nusser, Z. (2012). Release probability of hippocampal glutamatergic terminals scales with the size of the active zone. Nat. Neurosci. *15*, 988–997.

Indriati, D.W., Kamasawa, N., Matsui, K., Meredith, A.L., Watanabe, M., and Shigemoto, R. (2013). Quantitative localization of $Ca_v2.1$ (P/Q-type) voltage-dependent calcium channels in Purkinje cells: somatodendritic gradient and distinct somatic coclustering with calcium-activated potassium channels. J. Neurosci. *33*, 3668–3678.

Ishikawa, T., Nakamura, Y., Saitoh, N., Li, W.B., Iwasaki, S., and Takahashi, T. (2003). Distinct roles of Kv1 and Kv3 potassium channels at the calyx of Held presynaptic terminal. J. Neurosci. *23*, 10445–10453.

Kandler, K., and Friauf, E. (1993). Pre- and postnatal development of efferent connections of the cochlear nucleus in the rat. J. Comp. Neurol. *328*, 161–184.

Kiyonaka, S., Wakamori, M., Miki, T., Uriu, Y., Nonaka, M., Bito, H., Beedle, A.M., Mori, E., Hara, Y., De Waard, M., et al. (2007). RIM1 confers sustained activity and neurotransmitter vesicle anchoring to presynaptic Ca^{2+} channels. Nat. Neurosci. *10*, 691–701.

Kochubey, O., Han, Y., and Schneggenburger, R. (2009). Developmental regulation of the intracellular Ca^{2+} sensitivity of vesicle fusion and Ca^{2+}-secretion coupling at the rat calyx of Held. J. Physiol. *587*, 3009–3023.

Koike-Tani, M., Kanda, T., Saitoh, N., Yamashita, T., and Takahashi, T. (2008). Involvement of AMPA receptor desensitization in short-term synaptic depression at the calyx of Held in developing rats. J. Physiol. *586*, 2263–2275.

Kulik, A., Nakadate, K., Hagiwara, A., Fukazawa, Y., Luján, R., Saito, H., Suzuki, N., Futatsugi, A., Mikoshiba, K., Frotscher, M., and Shigemoto, R. (2004). Immunocytochemical localization of the alpha 1A subunit of the P/Q-type calcium channel in the rat cerebellum. Eur. J. Neurosci. *19*, 2169–2178.

Markram, H., Roth, A., and Helmchen, F. (1998). Competitive calcium binding: implications for dendritic calcium signaling. J. Comput. Neurosci. *5*, 331–348.

Meinrenken, C.J., Borst, J.G., and Sakmann, B. (2002). Calcium secretion coupling at calyx of Held governed by nonuniform channel-vesicle topography. J. Neurosci. *22*, 1648–1667.

Mercer, A.J., Chen, M., and Thoreson, W.B. (2011). Lateral mobility of presynaptic L-type calcium channels at photoreceptor ribbon synapses. J. Neurosci. *31*, 4397–4406.

Mintz, I.M., Sabatini, B.L., and Regehr, W.G. (1995). Calcium control of transmitter release at a cerebellar synapse. Neuron *15*, 675–688.

Miyazaki, T., Yamasaki, M., Hashimoto, K., Yamazaki, M., Abe, M., Usui, H., Kano, M., Sakimura, K., and Watanabe, M. (2012). $Ca_v2.1$ in cerebellar Purkinje cells regulates competitive excitatory synaptic wiring, cell survival, and cerebellar biochemical compartmentalization. J. Neurosci. *32*, 1311–1328.

Nadkarni, S., Bartol, T.M., Sejnowski, T.J., and Levine, H. (2010). Modelling vesicular release at hippocampal synapses. PLoS Comput. Biol. *6*, e1000983.

Nägerl, U.V., Novo, D., Mody, I., and Vergara, J.L. (2000). Binding kinetics of calbindin-D(28k) determined by flash photolysis of caged Ca(2+). Biophys. J. *79*, 3009–3018.

Neher, E., and Augustine, G.J. (1992). Calcium gradients and buffers in bovine chromaffin cells. J. Physiol. *450*, 273–301.

Oertel, D. (1999). The role of timing in the brain stem auditory nuclei of vertebrates. Annu. Rev. Physiol. *61*, 497–519.

Pan, B., and Zucker, R.S. (2009). A general model of synaptic transmission and short-term plasticity. Neuron *62*, 539–554.

Rozov, A., Burnashev, N., Sakmann, B., and Neher, E. (2001). Transmitter release modulation by intracellular Ca^{2+} buffers in facilitating and depressing nerve terminals of pyramidal cells in layer 2/3 of the rat neocortex indicates a target cell-specific difference in presynaptic calcium dynamics. J. Physiol. *531*, 807–826.

Sakaba, T., and Neher, E. (2001). Quantitative relationship between transmitter release and calcium current at the calyx of held synapse. J. Neurosci. *21*, 462–476.

Sätzler, K., Söhl, L.F., Bollmann, J.H., Borst, J.G., Frotscher, M., Sakmann, B., and Lübke, J.H. (2002). Three-dimensional reconstruction of a calyx of Held and its postsynaptic principal neuron in the medial nucleus of the trapezoid body. J. Neurosci. *22*, 10567–10579.

Schmidt, H., Brachtendorf, S., Arendt, O., Hallermann, S., Ishiyama, S., Bornschein, G., Gall, D., Schiffmann, S.N., Heckmann, M., and Eilers, J. (2013). Nanodomain coupling at an excitatory cortical synapse. Curr. Biol. *23*, 244–249.

Schneggenburger, R., and Neher, E. (2005). Presynaptic calcium and control of vesicle fusion. Curr. Opin. Neurobiol. *15*, 266–274.

Scimemi, A., and Diamond, J.S. (2012). The number and organization of Ca^{2+} channels in the active zone shapes neurotransmitter release from Schaffer collateral synapses. J. Neurosci. *32*, 18157–18176.

Sheng, J., He, L., Zheng, H., Xue, L., Luo, F., Shin, W., Sun, T., Kuner, T., Yue, D.T., and Wu, L.G. (2012). Calcium-channel number critically influences synaptic strength and plasticity at the active zone. Nat. Neurosci. *15*, 998–1006.

Stanley, E.F. (1993). Single calcium channels and acetylcholine release at a presynaptic nerve terminal. Neuron *11*, 1007–1011.

Südhof, T.C. (2013). Neurotransmitter release: the last millisecond in the life of a synaptic vesicle. Neuron *80*, 675–690.

Takahashi, T., Hori, T., Nakamura, Y., and Yamashita, T. (2012). Patch-clamp recording method in slices for studying presynaptic mechanisms. In Patch Clamp Techniques: From Beginning to Advanced Protocols, Y. Okada, ed. (Tokyo: Springer Japan), pp. 137–145.

Taschenberger, H., and von Gersdorff, H. (2000). Fine-tuning an auditory synapse for speed and fidelity: developmental changes in presynaptic waveform, EPSC kinetics, and synaptic plasticity. J. Neurosci. *20*, 9162–9173.

Taschenberger, H., Leão, R.M., Rowland, K.C., Spirou, G.A., and von Gersdorff, H. (2002). Optimizing synaptic architecture and efficiency for high-frequency transmission. Neuron *36*, 1127–1143.

Taschenberger, H., Scheuss, V., and Neher, E. (2005). Release kinetics, quantal parameters and their modulation during short-term depression at a developing synapse in the rat CNS. J. Physiol. *568*, 513–537.

Vyleta, N.P., and Jonas, P. (2014). Loose coupling between Ca^{2+} channels and release sensors at a plastic hippocampal synapse. Science *343*, 665–670.

Wang, L.Y., Fedchyshyn, M.J., and Yang, Y.M. (2009). Action potential evoked transmitter release in central synapses: insights from the developing calyx of Held. Mol. Brain *2*, 36.

Watanabe, H., Yamashita, T., Saitoh, N., Kiyonaka, S., Iwamatsu, A., Campbell, K.P., Mori, Y., and Takahashi, T. (2010). Involvement of Ca^{2+} channel synprint site in synaptic vesicle endocytosis. J. Neurosci. *30*, 655–660.

Weber, A.M., Wong, F.K., Tufford, A.R., Schlichter, L.C., Matveev, V., and Stanley, E.F. (2010). N-type Ca^{2+} channels carry the largest current: implications for nanodomains and transmitter release. Nat. Neurosci. *13*, 1348–1350.

Xu, T., Naraghi, M., Kang, H., and Neher, E. (1997). Kinetic studies of Ca^{2+} binding and Ca^{2+} clearance in the cytosol of adrenal chromaffin cells. Biophys. J. *73*, 532–545.

Yang, Y.M., Fedchyshyn, M.J., Grande, G., Aitoubah, J., Tsang, C.W., Xie, H., Ackerley, C.A., Trimble, W.S., and Wang, L.Y. (2010). Septins regulate developmental switching from microdomain to nanodomain coupling of Ca(2+) influx to neurotransmitter release at a central synapse. Neuron *67*, 100–115.

Young, S.M., Jr., and Neher, E. (2009). Synaptotagmin has an essential function in synaptic vesicle positioning for synchronous release in addition to its role as a calcium sensor. Neuron *63*, 482–496.

Neuron, Volume *85*

Supplemental Information

Nanoscale Distribution of Presynaptic Ca^{2+} channels and Its Impact on Vesicular Release during Development

Yukihiro Nakamura, Harumi Harada, Naomi Kamasawa, Ko Matsui, Jason S. Rothman, Ryuichi Shigemoto, R. Angus Silver, David A. DiGregorio, and Tomoyuki Takahashi

Inventory of supplemental information

Supplemental Data

Figure S1 (related to Figures 1 and 2). Comparison of $Ca_v2.1$ gold particle labeling in control and in $Ca_v2.1$ knockout mice, and determination of criterion for cluster analysis

Figure S2 (related to Figures 1 and 2). Application of cluster criterion to SDS-FRL images, and co-staining of $Ca_v2.1$ and RIM to identify active zones

Figure S3 (related to Figure 3). Estimation of confocal spot point spread function

Figure S4 (related to Figure 3). Spatial dependence of measured and simulated AP-evoked Ca^{2+} transients

Figure S5 (related to Figure 3). Simulation of developmental changes in AP-evoked Ca^{2+} transients

Figure S6 (related to Figure 5). Pipette perfusion control experiments

Figure S7 (related to Figure 5). Spatial distribution of presynaptic Ca^{2+} influx during development

Figure S8 (related to Figures 6 and 8). Influence of model parameters on vesicular release simulations

Table S1 (related to Figure 4) Model parameters for simulations of fluorescence Ca^{2+} transients

Table S2 (related to Figures 6, 7 and 8) Model parameters for simulations of Ca^{2+} diffusion and vesicular release

Supplemental Experimental Procedures

- Animals
- Electron microscopy and analysis of SDS-digested freeze-fracture replica labeling
  - SDS-FRL
  - Immunoparticle distribution analysis
  - Estimation of labeling efficiency
- Slice electrophysiology and Ca^{2+} imaging
  - Brainstem slice preparation
  - Electrophysiological recordings and data analysis
  - Measurements and analysis of Ca^{2+} transients
- Numerical simulations of Ca^{2+} reaction-diffusion and vesicular release
  - Simulations of fluorescence Ca^{2+} transients
  - Simulations of nanoscale [Ca^{2+}] and vesicular release

Supplemental References

SUPPLEMENTAL DATA

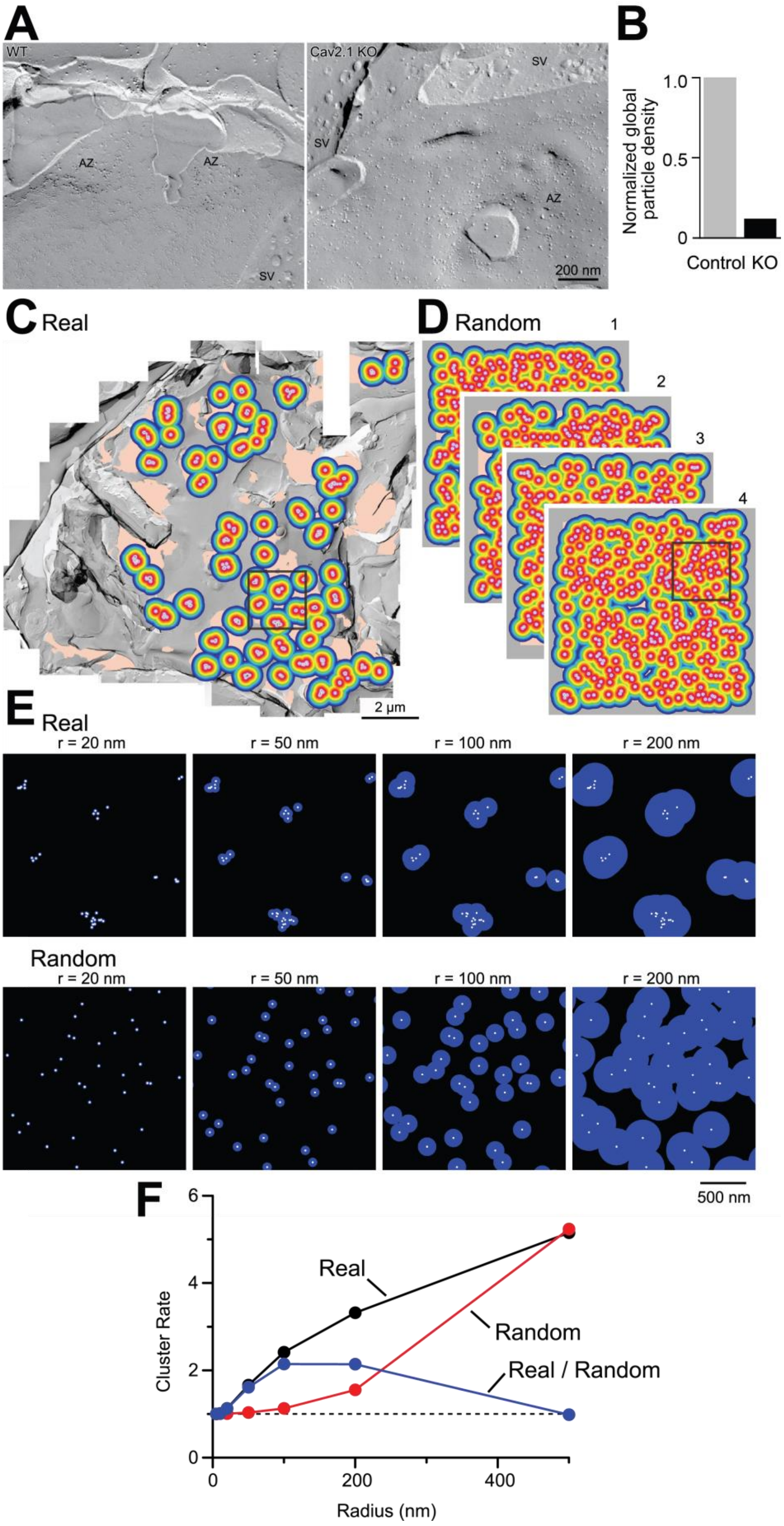

Figure S1. Comparison of $Ca_v2.1$ Gold Particle Labeling in Control and in $Ca_v2.1$ Knockout Mice, and Determination of Criterion for Cluster Analysis

(A) SDS-FRL immunogold labeling of $Ca_v2.1$ at the presynaptic P-face of the calyx of Held of a wild-type mouse (WT, left panel, P13). Presence of synaptic vesicles (SVs) in the cross-fracture through the cytosol confirms that this is the presynaptic P-face. As in rats, immunogold particles were mostly found as clusters. In a $Ca_v2.1$ knockout (KO) mouse (right panel, P13), the clustered pattern of immunogold labeling was not observed in the presynaptic P-face, not even near concaved surface with dimples, which indicates putative AZs.

(B) Global immunogold particle density of wild-type and $Ca_v2.1$ KO mice, both normalized to wild-type levels. The mean density for $Ca_v2.1$ KO mice was no more than 10% of wild-type (not shown), similar to the background level and confirming the specificity of the antibody (n = 2 murine calyces for both).

(C) SDS-FRL immunogold labeling of $Ca_v2.1$ at the presynaptic P-face (pink area) of a calyx of Held from a P21 rat. Distance from each pixel to the nearest neighbor immunogold particle is expressed in a pseudocolor map. The nearest distance from the pixels on the blue line to the immunogold particle is 480 nm.

(D) The same number of immunogold particles was distributed randomly in a square area with the same size as the sum of the presynaptic P-face area exposed in (C). 100 of such random distributions were made and distance maps were created for all.

(E) Comparison of real immunogold particle clusters with randomly distributed particles. Enlarged 2 × 2 µm areas in (C) and (D) are shown. Each particle location is shown with a white dot. Blue circles with the specified radius (upper panel) were drawn around each particle. By systematically varying the radius of these circles, we probed the criteria that defined reasonable clusters. When the circle radius was 50 nm, circles showed some overlap in the real distribution, but almost no overlap in the random distribution. When the circle radius was 200 nm, circles nearly filled the entire field in the random distribution, but a substantial fraction of the field remained unoccupied by the circles in the real distribution.

(F) To search for the optimal circle radius for defining a cluster, we calculated the ‘cluster rate’ between real and random distributions. The area that the circles occupy (S_T) was divided by the area of a circle around a single particle (S_C). The total number of immunoparticles (N_P) was divided by S_T/ S_C and we defined this value (N_P S_C / S_T) as the “cluster rate”. If there is no overlap between circles, the cluster rate is 1. The cluster rate increases, however, when circles begin to overlap. With a large enough radius, the cluster rate approaches the total number of immunoparticles. As we increased the circle radius, the ‘cluster rate’ increased in both real and random distributions. The former slope was steeper than the latter below 100 nm, but above 200 nm ‘cluster rate’ in the random distribution increased more steeply. The maximal separation between random and real distributions was found at 100 nm, where the ‘cluster rate’ for the real distribution (black) divided by that for the random distribution (red) showed the largest value (blue).

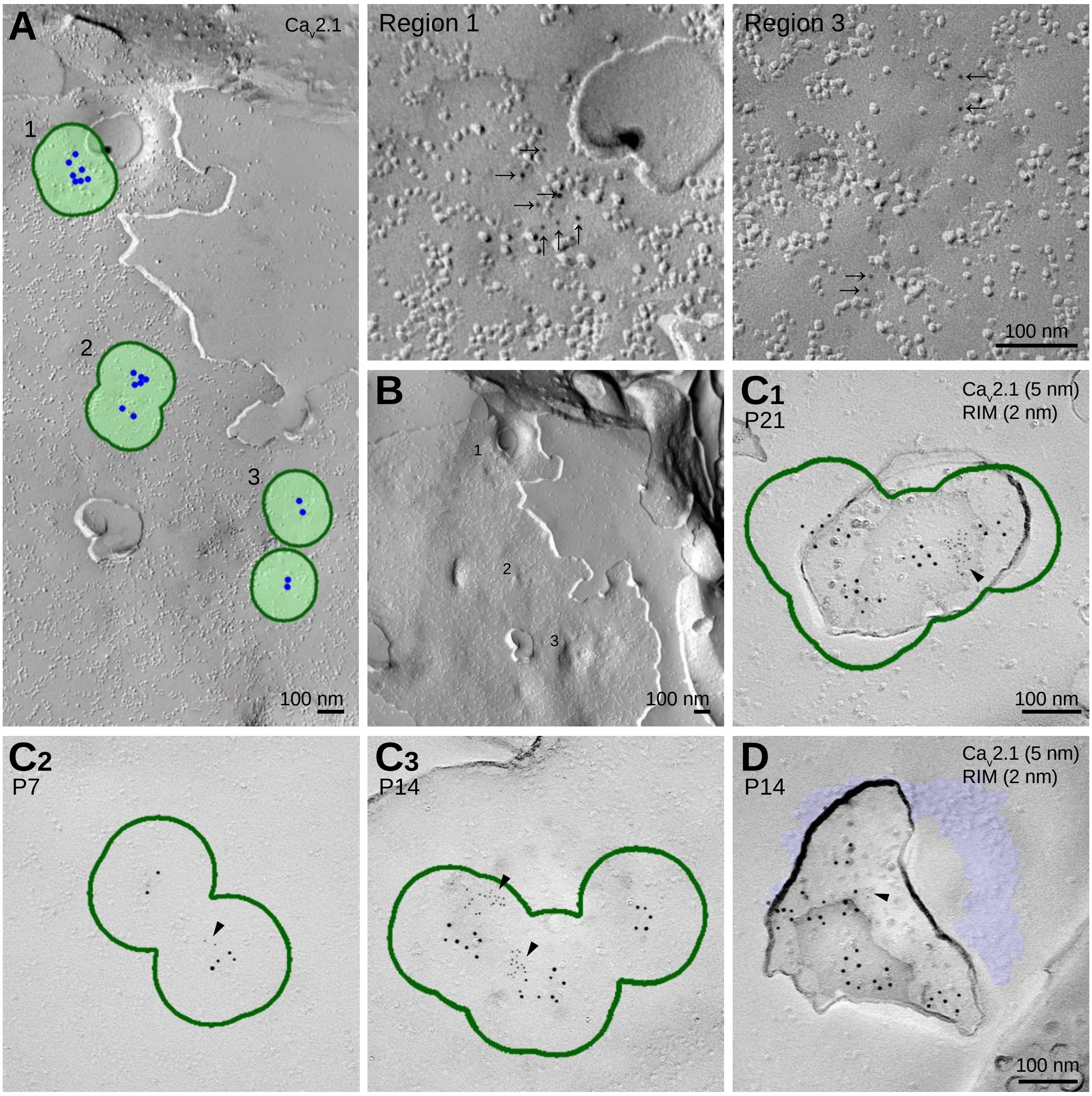

Figure S2. Application of Cluster Criterion to SDS-FRL Images, and Co-staining of Ca_v2.1 and RIM to Identify Active Zones

(A) *Left*: The replica image shows the same region as Figure 1A1. Ca_v2.1 particles are labeled with blue dots and cluster area is labeled with green. *Right*: Regions 1 and 3 are shown at a higher magnification. Region 2 is shown in Figure 1A2 and 1A3. Arrows indicate the location of each immunogold particle. Gold particles in region 3 are separated into two clusters according to our definition of cluster with a 100 nm radius. Note that NND of the two clusters is short (250 nm) in this case. Such a short NND observed in La1 was not seen in La2 with higher labeling efficiency. This is presumably because the lower channel labeling efficiency in La1 resulted in misclassifications of some large clusters as two closely space clusters.

(B) The same region as (A) viewed with a higher tilt (40 degrees) shows clusters 1-3 on concaved surface. Ca_v2.1 particle clusters are often found near such a concaved surface with dimples.

(C) Representative images of double immunogold labeling of Ca_v2.1 and RIM. Immunogold particles for RIM (2 nm, marked with arrowheads) were detected close to Ca_v2.1 particles (5 nm) in P21 (C1), P7

(C2), and P14 (C3, D) replicas, falling within the defined area of a cluster (green line), supporting that $Ca_v2.1$ clusters are located in AZs at all ages examined.

(D) One example of replica image showing a transition of the postsynaptic exoplasmic face to the presynaptic P-face within a synapse. An intra-membrane particle cluster indicating excitatory postsynaptic site on the exoplasmic face is labeled with light purple. Immunogold particles for $Ca_v2.1$ were observed in a small "window" showing the presynaptic P-face continuous to the postsynaptic site. Scale bars are 100 nm.

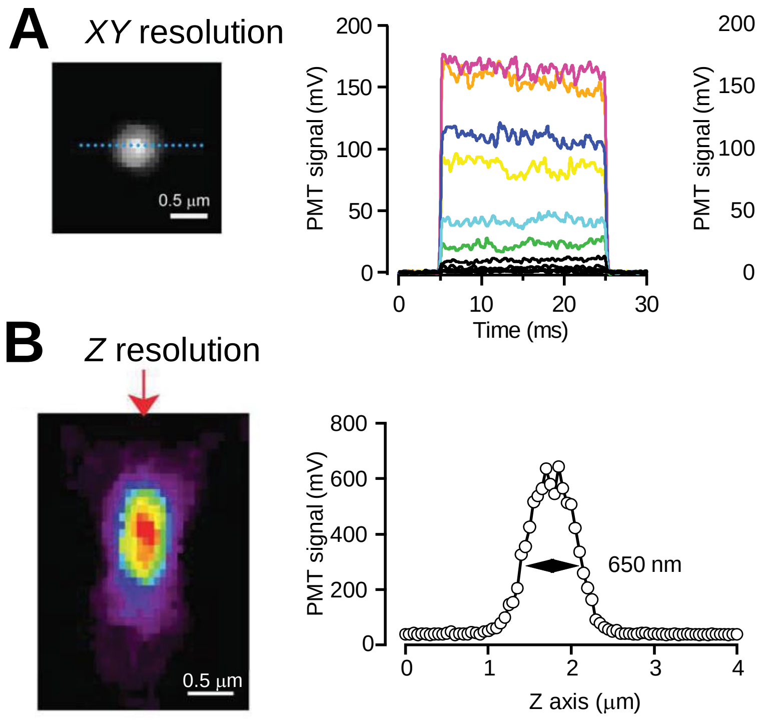

Figure S3. Estimation of Confocal Spot Point Spread Function

(A) *Left*: Confocal laser scanning image of a 100 nm green fluorescence bead with light blue dots indicating spot locations for the point scan. *Center*: Fluorescence traces recorded when the confocal spot is placed at locations along a line crossing the fluorescence bead in 80 nm steps. *Right*: Fluorescence intensity of each trace versus spot location from a representative scan. The fluorescence intensity during the 20 ms laser pulse was averaged. Red trace is a Gaussian fit from which the full width at half maximum was determined. The average full width at half maximum from 5 different beads was 220 ± 3 nm.

(B) *Left*: *x-z* plot of the scanning confocal point spread function created by imaging a 100 nm fluorescent bead. Focal planes were adjusted using a piezoelectric drive in 50 nm steps. Red arrow indicates the location of the intensity line profile along the *z*-axis. *Right*: *z*-axis intensity line profile (circles; average of 9 neighboring pixels) and its Gaussian fit (solid line). The average full width at half maximum of Gaussian fits *x-z* plots from image stacks of 6 different fluorescence beads was 650 ± 12 nm.

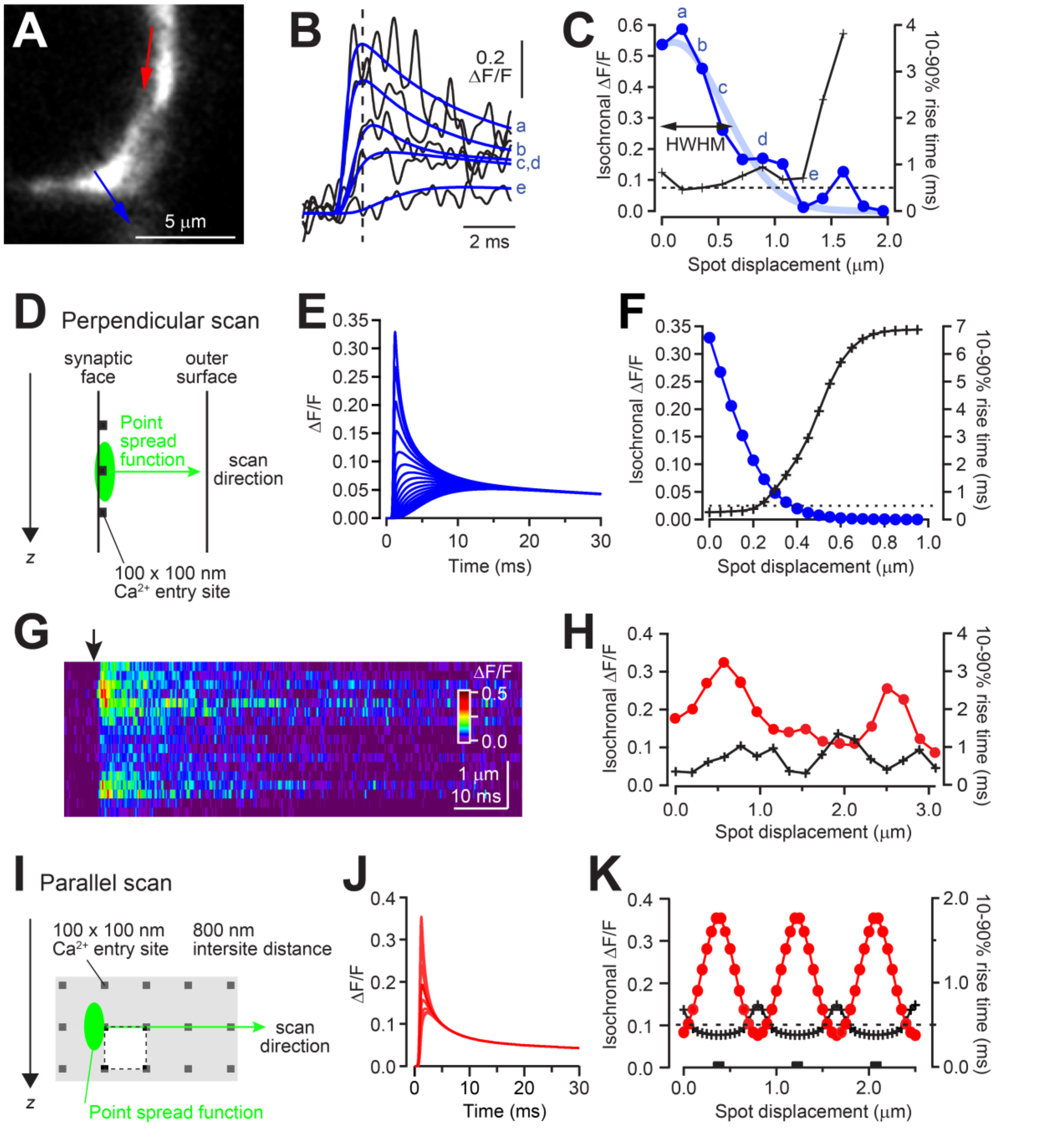

Figure S4. Spatial Dependence of Measured and Simulated AP-evoked Ca^{2+} Transients

(A) Confocal spot recording locations using a systematic spacing perpendicular to (blue arrow for B and C) and along (red arrow for G and H) the synaptic face of a representative P8 calyx.

(B) Representative Ca^{2+} transients (black traces) recorded at locations displaced from synaptic face, and their multi-exponential fits. A dashed line indicates the peak time of the largest transient. The amplitude of each fitted trace at this time point was used for the isochronal $\Delta F/F$ plot.

(C) Isochronal $\Delta F/F$ amplitudes (left axis) and 10-90% rise time (right axis) of Ca^{2+} transients plotted against the distance of spot displacement (178 nm steps) away from the synaptic face. The thick blue line shows a Gaussian fit to data points, from which the 50% decay distance of $\Delta F/F$ half width at half maximal (HWHM) was estimated. Ca^{2+} transients exhibited a prominent amplitude reduction with spot displacement (50% reduction in amplitude at 0.7 ± 0.03 μm, *n* = 4 calyces). These results are consistent with the report that VGCC currents are exclusively localized at the synaptic face (Sheng et

al., 2012).

(D) A schematic figure showing the arrangement of Ca^{2+} entry-sites and confocal detection volume for numerical simulation of Ca^{2+} transient recording locations perpendicular to synaptic surface. The size of simulation field was 0.8 μm (*x*: thickness of the calyx) × 0.8 μm (*y*) × 1 μm (*z*), and divided into equal elementary voxel sizes of 50 nm cube for simulation purposes. Ca^{2+} entry site was simulated as a 100 × 100 nm square (to approximate cluster area in SDS-FRL experiments).

(E) Simulated Ca^{2+} transients from confocal spot locations separated by 50 nm and perpendicular to synaptic face (green arrow in D).

(F) Isochronal $\Delta F/F$ (blue circles) and 10-90% rise time (black crosses) of the simulated Ca^{2+} transients for perpendicular scan. Isochronal amplitudes were calculated over a 0.2 ms window around the peak of the largest transient.

(G) A kymograph showing confocal spot recorded $\Delta F/F$ traces along synaptic face from the P8 calyx. Each trace was recorded at locations separated by 193 μm. An arrow indicates the AP peak time.

(H) Isochronal $\Delta F/F$ amplitudes of Ca^{2+} transients (red circles) and 10-90% rise time (black crosses) of Ca^{2+} transients plotted against spot displacement along synaptic face.

(I) A schematic figure showing the arrangement of Ca^{2+} entry-sites and confocal detection volume for parallel scan in simulation. A dashed square indicates simulation volume.

(J) Simulated Ca^{2+} transients from confocal spot locations separated by 50 nm and parallel to the synaptic surface. Scan direction is shown as a green arrow in (I).

(K) Isochronal $\Delta F/F$ amplitude (red circles) and 10-90% rise time (black crosses) of the simulated Ca^{2+} transients from (J). Black bars show the locations of Ca^{2+} entry site. Dashed line indicates rise time criterion (0.5 ms).

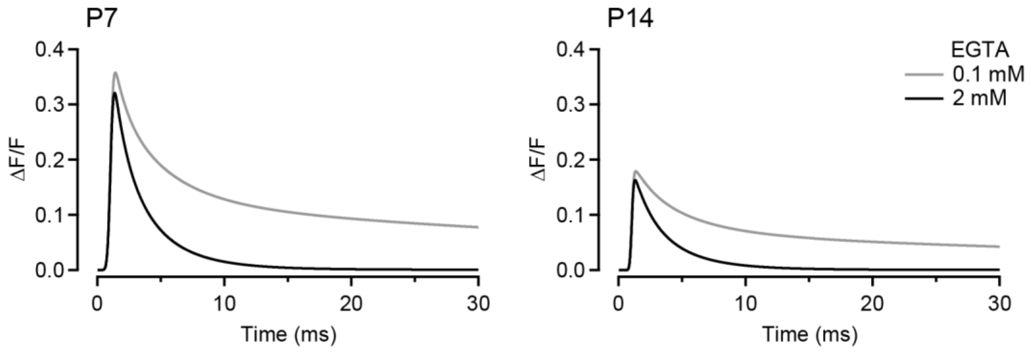

Figure S5. Simulation of Developmental Changes in AP-evoked Ca^{2+} Transients

Simulations of confocal spot detected Ca^{2+} transients at P7 and P14 using low-affinity endogenous fixed buffer (EFB, κ = 40) with 0.1 mM (gray) or 2 mM (black) EGTA in the internal solution. The Ca^{2+} diffusion model reproduced well the time course and amplitude of the experimentally measured Ca^{2+} transients at P7 and P14 (see Figures 4A and 4B). The number of open VGCCs was adjusted to match the amplitude of the peak ΔF/F between simulations and data (13 VGCCs for P7 and 11 VGCCs for P14).

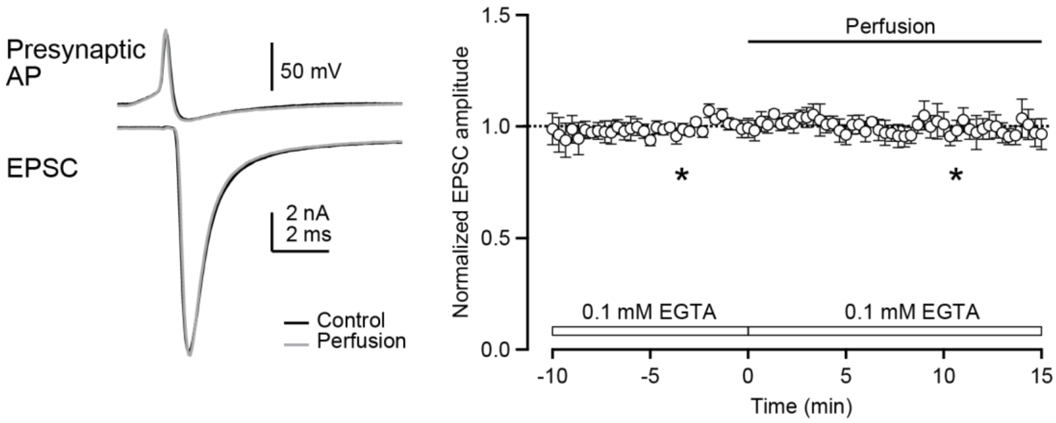

Figure S6. Pipette Perfusion Control Experiments

Left: Presynaptic AP and EPSCs 10 min after patch perfusion (gray trace) were indistinguishable from those recorded 5 min before patch perfusion (black trace, superimposed). *Right*: Time plot of EPSC amplitude when the internal solution of the same composition (0.1 mM EGTA) was perfused into the terminal at time zero. Data points are mean and SEM calculated from 7 experiments. The EPSC amplitudes were normalized to the mean EPSC amplitude calculated from EPSCs recorded during the 5 min before the onset of perfusion. Asterisks indicate the times at which sample records (left traces, superimposed) were collected.

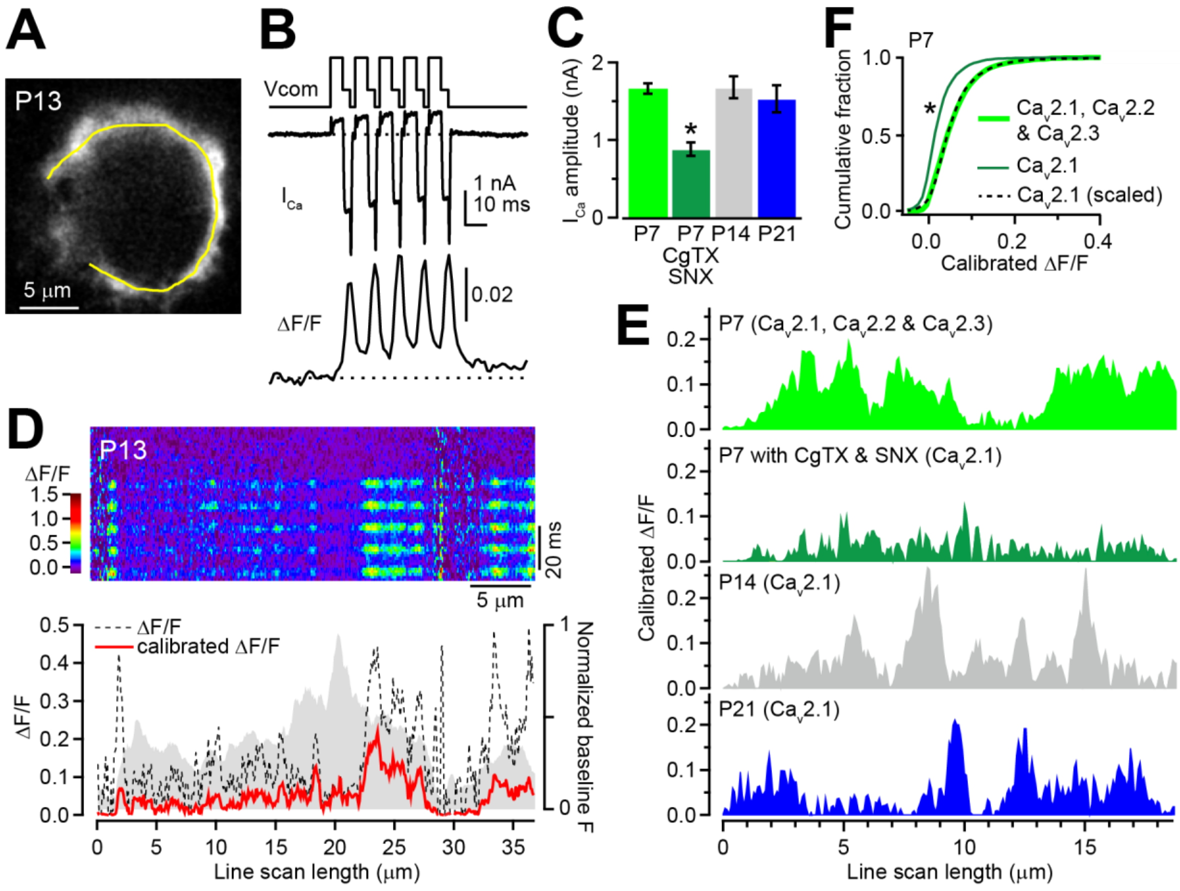

Figure S7. Spatial Distribution of Presynaptic Ca^{2+} Influx during Development

To examine the contribution of VGCC subtypes to the spatial distribution of Ca^{2+} influx, we performed confocal line scan Ca^{2+} imaging along the synaptic face of the calyx in response to voltage steps with and without subtype specific VGCC blockers. We used the low-affinity dye Oregon Green BAPTA-5N as a linear reporter of Ca^{2+} entry, and 10 mM EGTA to constrain the distribution of free Ca^{2+} to near Ca^{2+} entry sites.

(A) Confocal fluorescence image of a P13 calyx of Held loaded with 40 µM Alexa594 and 100 µM Oregon Green BAPTA-5N. Yellow line denotes the line scan region.

(B) Whole-terminal Ca^{2+} current (I_{Ca}, middle trace) evoked by voltage steps from –80 mV to +80 mV for 5 ms then to 0 mV for 3 ms (V_{com}, top trace). Bottom trace is a spatial average of the fluorescence change (*Δ*F/F; average of 3 trials) from the line scan in (D).

(C) The steady-state amplitude of I_{Ca} remained similar throughout development: at P7 (*n* = 23 calyces), P14 (*n* = 10), P21 (*n* = 6) and P7 calyces in the presence of CgTX and SNX (*n* = 11, **P* < 0.05, one way ANOVA followed by Tuckey's post hoc between P7 (toxins) and all other groups).

(D) Linescan images (kymograph) and spatial profiles of *Δ*F/F exhibited hot spots of fluorescence changes along the scan region. *Top*: Average *Δ*F/F line scan (*n* = 3 trials) in response to the voltage step shown in (A). Acquisition rate was 1.2 ms/line. *Bottom*: Spatial distribution of resting fluorescence (gray, normalized to its maximum value), *Δ*F/F (dotted), and calibrated *Δ*F/F (red) along the line scan length. *Δ*F/F values were obtained from temporal averages of 3 ms during a depolarizing step to 0 mV and averaged from 15 such image segments (5 per scan × 3 line scan images). The calibrated the *Δ*F/F profile was used to normalize for terminal dimensions smaller than the microscope point spread

function, and calculated by dividing ΔF/F values at each pixel location by the relative resting fluorescence (calculated from the ratio of resting fluorescence at each spatial increment and the maximal resting fluorescence of the linescan).

(E) Spatial distribution of Ca^{2+} influx, expressed as calibrated ΔF/F, in a P7 calyx in the presence of CgTX and SNX, in a P14 calyx and in a P21 calyx.

(F) The spatial distribution was quantified using cumulative amplitude distributions of calibrated ΔF/F at P7 calyces in the absence (*n* = 39 scans from 23 calyces) and presence of CgTX and SNX (TX; *n* = 29 scans from 13 calyces). Dashed line is the ΔF/F distribution in the presence of toxins after normalizing by the median ΔF/F. There was no significant difference in the spatial distribution of Ca^{2+} entry with and without these toxins (*P* = 0.96, Kolmogorov-Smirnov test), suggesting that $Ca_v2.2$ and $Ca_v2.3$ have a similar distribution to $Ca_v2.1$, given our submicron resolution (see Figure S3).

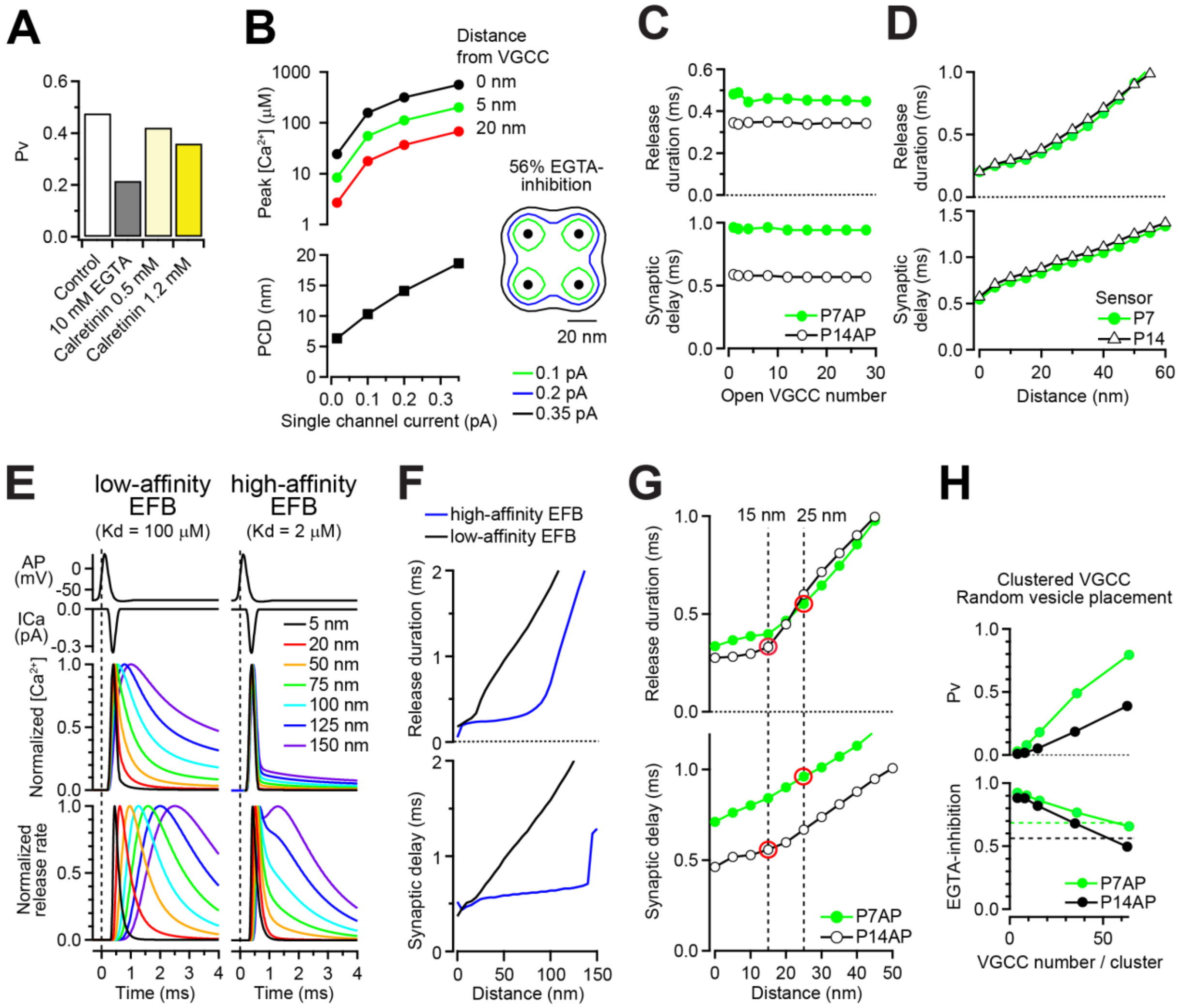

Figure S8. Influence of Model Parameters on Vesicular Release Simulations

(A) The effect of calretinin on P_v. P_v was estimated using four open VGCCs with an NND of 35 nm. For these simulations, we used rate constants for the independent T-site and R-site of calretinin (Table S2). The presence of calretinin at 1.2 mM (yellow bar) only had a modest effect on P_v with respect to control conditions.

(B) The influence of the single VGCC current amplitude on peak Ca^{2+} concentration (*top left*) and *perimeter coupling distance* (PCD; *bottom left*). Right panel indicates the EGTA-inhibition of P_v contour lines (56%) around the channel cluster composed of four open VGCCs with NND of 35 nm, assuming the single VGCC current amplitude as 0.35 pA (black), 0.2 pA (blue) and 0.1 pA (green). These simulations indicate that release is likely to occur from the perimeter of clusters for single channel conductances greater than ~0.2 pA.

(C) Synaptic delay and release duration plotted against the VGCC number within a cluster for perimeter release model simulations (PCD= 26-34 nm for P7 and 18-21 nm for P14). Although the number of open VGCC in response to AP decreased due to acceleration of AP from P7 to P14 (Yang and Wang, 2006), changing the number of open VGCCs had no effect on synaptic delay or release duration.

(D) Effects of developmental changes in Ca^{2+} sensitivity of vesicular sensors on release time course. Changes in Ca^{2+} sensor from P7 to the lower Ca^{2+} affinity sensor for P14 had little effect on release

duration and synaptic delay. Both sensor simulations were performed with an AP_7 waveform.

(E) Ca^{2+} reaction-diffusion and vesicular release simulation using a low-affinity (K_d = 100 μM, *left*) or high affinity EFB (K_d = 2 μM, *right*). For each condition, the time courses of Ca^{2+} transients (middle raw) and release rate (bottom raw) for different membrane voxels (distances from cluster perimeter are color-coded).

(F) Effects of EFB property on release duration and synaptic delay assuming high-affinity (blue trace) or low-affinity (black trace) EFB. The high-affinity buffer produces a release duration that is largely insensitive to PCD values between 10 and 100 nm.

(G) Dependence of release duration (upper panel) and synaptic delay (lower panel) on the PCD for P7 (green) and P14 (black), simulated using the perimeter release model with stochastic channel opening (Figure 7). Red circles indicate values predicted by experimental results.

(H) Initial P_v and EGTA-inhibition of P_v in random vesicle placement model. In this simulation, we assumed that synaptic vesicles primed for release were randomly distributed within AZ containing a single VGCC cluster. In this arrangement, the AZ P_v was calculated by simply averaging P_v from all locations within the AZ, assuming an area of 0.05 μm^2 (Taschenberger et al., 2002). Although the AZ P_v linearly increased with the number of open VGCC within the cluster, the mean number of VGCCs necessary to match experimental EGTA-inhibition values at P7 and P14 (dashed lines), was far greater than the mean number estimated from EM experiments (Figure 2E).

Table S1. Model Parameters for Simulations of Fluorescence Ca^{2+} Transients

Simulation Parameters	value	units	Reference
Simulation voxel size	50	nm	
Time step for simulation	0.38	μs	
Full width at half maximum of 3D Gaussian point spread function	220 (*x-y*)<br>700 (*z*)	nm	Figure S3
Ca^{2+} entry			
Maximal single channel current during AP	0.28 (P7)<br>0.35 (P14)	pA	Calculated from Sheng et al.,2012 and Figure 3F
Time course of Ca^{2+} entry			Taken from Figure 3F
Size of Ca^{2+} entry site	100 × 100	nm	Calculated from Figure 2C
Diffusion coefficient	0.22	$\mu m^2\ ms^{-1}$	Allbritton et al, 1992
Basal Ca^{2+} concentration	10	nM	Measured
Ca^{2+} extrusion	0.4	ms^{-1}	Helmchen et al, 1997
Endogenous fixed buffer properties			
k_{on}	100	$mM^{-1}ms^{-1}$	Xu et al., 1997
k_{off}	10	ms^{-1}	
Total concentration	4.0	mM	Calculated from Helmchen et al, 1997
ATP calcium binding properties			
k_{on}	500	$mM^{-1}ms^{-1}$	Naraghi & Neher, 1997
k_{off}	100	ms^{-1}	
Diffusion coefficient	0.2	$\mu m^2\ ms^{-1}$	
Total concentration available to bind Ca^{2+}	0.65	mM	calculated using Maxchelator
EGTA buffer properties			
k_{on}	10.5	$mM^{-1}ms^{-1}$	Nägerl et al., 2000
k_{off}	0.000735	ms^{-1}	
Diffusion coefficient	0.22	$\mu m^2\ ms^{-1}$	Naraghi & Neher, 1997
Total concentration	0.1, 2.0 or 10	mM	Experimental values
Calcium indicator (Oregon Green BAPTA-5N) properties			
k_{on}	240	$mM^{-1}ms^{-1}$	Faas et al., 2007
k_{off}	8.67	ms^{-1}	
Concentration	0.1	mM	Experimental value
Diffusion coefficient	0.1	$\mu m^2\ ms^{-1}$	Gabso et al., 1997
Ratio of maximum to minimum fluorescence	11.8		Bollmann and Sakmann, 2005

Table S2. Model Parameters for Simulations of Ca^{2+} Diffusion and Vesicular Release

Simulation Parameters	value	units	Reference
Simulation voxel size	5	nm	
Time step for Ca simulation	0.01515	μs	
Time step for release simulation	10	μs	
Ca^{2+} entry			
Maximal single channel current during AP	0.28 (P7)<br>0.35 (P14)	pA	Calculated from Sheng et al.,2012 and Figure 3F
Time course of Ca^{2+} entry			Taken from Figure 3F
Diffusion coefficient	0.22	$\mu m^2\ ms^{-1}$	Allbritton et al, 1992
Basal Ca^{2+} concentration	10	nM	Measured
Endogenous fixed buffer properties			
k_{on}	100	$mM^{-1}ms^{-1}$	Xu et al,. 1997
k_{off}	10	ms^{-1}	
Total concentration	4.0	mM	Calculated from Helmchen et al, 1997
ATP calcium binding properties			
k_{on}	500	$mM^{-1}ms^{-1}$	Naraghi & Neher, 1997
k_{off}	100	ms^{-1}	
Diffusion coefficient	0.2	$\mu m^2\ ms^{-1}$	
Total concentration available to bind Ca^{2+} (with 0.1 mM EGTA)	0.176	mM	calculated using Maxchelator
Total concentration available to bind Ca^{2+} (with 10 mM EGTA)	0.27	mM	
EGTA buffer properties			
k_{on}	10.5	$mM^{-1}ms^{-1}$	Nägerl et al., 2000
k_{off}	0.000735	ms^{-1}	
Diffusion coefficient	0.22	$\mu m^2\ ms^{-1}$	Naraghi & Neher, 1997
Total concentration	0.1 or 10	mM	Experimental values
Calretinin			
T site k_{on}	1.8	$mM^{-1}ms^{-1}$	Faas et al., 2007
T site k_{off}	0.053	ms^{-1}	
R site k_{on}	310	$mM^{-1}ms^{-1}$	
R site k_{off}	0.02	ms^{-1}	
Diffusion coefficient	0.02	$\mu m^2\ ms^{-1}$	Adopted the value for Calbindin D28k, Schmidt et al., (2005)
Total concentration	0.5 or 1.2	mM	Edmonds et al., 2000
Ca^{2+} sensor for release			
k_{on}	115 (P14)<br>121 (P8)	$mM^{-1}ms^{-1}$	Kochubey et al., 2009
k_{off}	7.9 (P14)<br>6.5 (P8)	ms^{-1}	
Cooperativity factor β	0.26		
Vesicular fusion rate γ	0.696	ms^{-1}	

SUPPREMENTAL EXPERIMENTAL PROCEDURES

Animals

Animal experiments were conducted in accordance with the guidelines of Doshisha University (Kyoto Japan), the National Institute for Physiological Sciences (Okazaki, Japan) and Institut Pasteur (Paris, France). Wistar rats and C57BL/6J mice of both sexes were raised on a 12 h light/dark cycle with water and food *ad libitum.*

Electron Microscopy and Analysis of SDS-Digested Freeze-Fracture Replica Labeling

SDS-FRL

Wistar rats at postnatal days (P) 7-8, 14-15, and 21 (*n* = 2, 4, and 5 animals, respectively) and P13 $Ca_v2.1$ knock-out (KO) and P13 wild-type mice were used. SDS-FRL was performed with some modifications (Masugi-Tokita and Shigemoto, 2007; Masugi-Tokita et al., 2007; Budisantoso et al., 2012; Indriati et al., 2013) of the technique developed by Fujimoto (Fujimoto, 1995). The rats were anesthetized with sodium pentobarbital (50 mg/kg, i.p.) and perfused transcardially with 25 mM phosphate buffered saline solution (PBS) for 1 min, followed by perfusion with 2% paraformaldehyde (PFA) and 15% saturated picric acid in 0.1 M phosphate buffer (PB) for 12 min. Coronal slices (130 μm thick) were cut using a vibrating microslicer (Pro7, Dosaka, Kyoto) in 0.1 M PB. A region of the MNTB was trimmed from the slices and immersed in graded glycerol of 10-30% in 0.1 M PB at 4°C 1 overnight and frozen by a high pressure freezing machine (HPM010; BAL-TEC). Frozen samples were fractured into two parts at -140°C and replicated by carbon deposition (5 nm thick), carbon-platinum (uni-direction from 60°, 2 nm) and carbon (20 nm) in a freeze-fracture replica machine (JFD II, JEOL, Tokyo). Tissue debris was dissolved with gentle shaking at 80°C for 18 h or 24 h in a solution containing 15 mM Tris-HCl (pH 8.3), 20% sucrose, and 2.5% SDS. The replicas were washed three times in 50 mM Tris-buffered saline (TBS, pH 7.4) containing 0.05% bovine serum albumin (BSA), 0.1% Tween-20, and 0.05% sodium azide and blocked with 5% BSA in the washing buffer for 1 h at room temperature. The replicas were then incubated with the guinea pig primary antibody against $Ca_v2.1$ subunit of P/Q type Ca^{2+} channel (8.1 μg/ml, Miyazaki et al., 2012) overnight at 15°C followed by incubation with goat anti-guinea pig secondary antibodies conjugated with 5 nm gold particles (British Biocell International, Cardiff) overnight at 15°C. Identification of 5 nm particles on the replicas was based on their size, round shape, and electron density. Weaker density dots just next to intra-membrane particles are mostly shadows made by the platinum coating and they were excluded. The specificity of the $Ca_v2.1$ antibody was confirmed by testing MNTB tissue from $Ca_v2.1$ KO mice. Immunogold particles for $Ca_v2.1$ were mostly abolished in the KO mice tissue (Figures S1A and S1B). For the double immunolabeling, primary antibody against RIM1/2 (Synaptic Systems, Holderith et al., 2012) combined with anti-rabbit secondary antibodies conjugated with 2 nm gold particles (British Biocell International) was used in a sequential manner after completing the labeling for $Ca_v2.1$. To facilitate visualization of these small gold particles, carbon replicas (Loukanov et al., 2010) were used for the double labeling. Background labeling for RIM estimated on the E-face was 0.32 /μm^2. The probability of having a single particle for RIM by chance within the 100 nm radius (green lines in Figure S2) of average $Ca_v2.1$ clusters was calculated to be less than 1%. The ratios of $Ca_v2.1$ clusters double labeled with at least a single RIM

particle were 64, 87, and 74% for P7 (n = 28), P14 (n = 79) and P21 (n = 100) samples, respectively.

Immunoparticle Distribution Analysis

Quantification of the distribution of immunoparticles was carried out as follows. Images of presynaptic P-face were captured at a magnification of 46,000× with a digital camera (Veleta, Olympus-Soft Imaging System; OSIS). Images were analyzed with iTEM (OSIS) and FIJI software (distributed under the General Public License, GPL). Distances were calculated using macros in Excel (Microsoft). The XY coordinates of immunoparticles were recorded and extracted in iTEM and the distances from each particle to every other particles were calculated and the smallest value was assigned as the nearest neighbor distance (NND) for each particle. To find clusters of immunoparticles, binary images with particle locations represented as single pixel dots were created and a 100 nm radius circle was drawn around each particle using the Maximum filter in FIJI. The particles were considered as forming a cluster when these circles overlap. When local maxima were searched in the binary images with overlapping circles, a geometrical centroid, also referred to as the center of gravity in this article, for each overlapping circle was found. The average particle distribution across all clusters was assessed by aligning individual distributions on their center of gravity (cluster center). The NND between clusters was estimated by determining the shortest distance between cluster centers.

Estimation of Labeling Efficiency

Labeling efficiency of $Ca_v2.1$ was estimated by comparing the overall density of the $Ca_v2.1$ immunogold particles with the amplitude of whole-terminal I_{Ca}. In response to a voltage step to 0 mV, the amplitude of steady-state whole-terminal I_{Ca} was 1691 pA at P14 (Figure S7C). When this amplitude was divided by the single channel current (0.15 pA) and the channel open probability for $Ca_v2.1$ channel at 0 mV (0.50, Sheng et al, 2012), this gave an estimate of 22546 channels per terminal. This number is similar to the estimate using variance mean analysis (Lin et al, 2011). We next estimated the number of gold particles corresponding to the entire calyx for both labeling samples. To do this, we measured surface area of postsynaptic MNTB neurons and their coverage by calyces. The surface area of MNTB neuron was 1168 ± 68 μm^2 (n = 11), similar to the value previously reported for the calyx of Held at P14 (Taschenberger et al., 2002). As calyx covers 55.8% of the surface (Taschenberger et al., 2002) the synaptic surface area was estimated as 647 μm^2.

In La1 (P14) samples the gold particle density was 6.7 /μm^2, giving a total number of $Ca_v2.1$ gold particles for the entire calyx of 4338. The labeling efficiency was therefore estimated to be 19% (4338/22546). In La2 (P14) the particle density was 21.7 /μm^2 giving a total gold particle number of 13984. The labeling efficiency for La2 was then estimated to be 62% (13984/22546). This labeling efficiency of La2 was similar to previously reported using the same batch of antibody (Indriati et al, 2013). As the NND within cluster was not different between P7, P14 and P21 in both La1 (Figure 2F) and La2 (data not shown), we assumed the same labeling efficiency across ages.

Slice Electrophysiology and Ca^{2+} Imaging

Brainstem Slice Preparation

Brainstem slices were prepared from P7-21 Wistar rats. Briefly, rats were decapitated under halothane anesthesia and their brains were quickly removed. Transverse slices (150-250 μm thick) containing the

MNTB were cut using a tissue slicer (VT1200S; Leica Microsystems). The dissections and slicing were performed in ice-cold Ca^{2+}-free artificial cerebrospinal fluid (ACSF) containing (in mM) 125 NaCl, 2.5 KCl, 26 $NaHCO_3$, 1.25 NaH_2PO_4, 6 $MgCl_2$, 10 glucose, 3 myo-inositol, 2 sodium pyruvate and 0.5 ascorbic acid (pH 7.4 when bubbled with 95% O_2 and 5% CO_2). Slices were maintained in the standard ACSF at 37°C for 40-60 min and subsequently at room temperature. The composition of standard ACSF was the same as the Ca^{2+}-free ACSF except that $MgCl_2$ and $CaCl_2$ concentrations were 1 mM and 2 mM, respectively.

Electrophysiological Recordings and Data Analysis

Whole-cell patch-clamp recordings from calyces were made using Multiclamp 700A or Multiclamp 700B amplifier (Molecular Devices). All experiments were carried out at room temperature (22-24 ^{o}C). Patch pipettes were made from glass capillaries (GC150F-10, Harvard Apparatus, Kent) using a puller (P-1000, Sutter). For electrophysiological recordings combined with confocal presynaptic Ca^{2+} imaging, the pipette solution contained (in mM): 95 K-methanesulfonate, 30 KCl, 40 HEPES, 0.1 or 2 EGTA, 6 NaOH, 4 $MgCl_2$, 4 ATP-Na, 0.5 GTP-Na (pH adjusted to 7.3 with KOH, 295-305 mOsm), to which we added Oregon Green BAPTA 5N (100 μM, Invitrogen) for Ca^{2+} imaging and Alexa 594 (40 μM, Invitrogen) for visualizing the shape of calyces. In presynaptic voltage-clamp experiments, K-methanesulfonate and KCl were replaced with (in mM) 125 CsCl and 10 TEA-Cl. Sodium and potassium currents were blocked by adding tetrodotoxin (1 μM), TEA-Cl (10 mM) and 4-aminopirdine (100 μM) to perfusate. In line scan experiments (Figure S7), [EGTA] was raised to 10 mM to minimize diffusional blurring of $[Ca^{2+}]$ and Ca^{2+}-dependent VGCC inactivation at P7 (Nakamura et al., 2008). For simultaneous pre- and postsynaptic patch-clamp recordings, the presynaptic pipette solution contained (in mM): 90 K-methanesulfonate, 30 KCl, 3 K-glutamate, 40 HEPES, 12 Na_2 creatinephosphate, 0.1 EGTA, 1 $MgCl_2$, 2 ATP-Mg, 0.5 GTP-Na (pH adjusted to 7.3 with KOH, 315 mOsm). Free Ca^{2+} concentration in all presynaptic solutions was less than 10 nM (calculated using Maxchelator #46v8 (http://maxchelator.stanford.edu/), which was confirmed by fluorescent calibration using Oregon Green BAPTA1 (Invitrogen). The presynaptic pipette and series resistance were 4.5–7.0 MΩ and 6–22 MΩ, respectively, and routinely compensated by 80%. In voltage clamp experiments, linear leak and capacitive currents were subtracted using the scaled pulse (P/8) protocol. The liquid junction potential was not corrected. Presynaptic APs were evoked by brief current injection via a presynaptic pipette or by presynaptic fiber stimulation using an extracellular bipolar tungsten electrode.

The presynaptic pipette perfusion was performed as described previously (Takahashi et al, 2012). Briefly, under stereoscopic microscope observation, thin glass capillary (PT-030, Takao Manufacturing, Kyoto) containing the presynaptic patch solution with 10 mM EGTA was inserted into the presynaptic patch pipette. We set the distance between the tip of thin capillary and that of presynaptic patch pipette to be less than 200 μm. The tip of the inner glass capillary was connected to a picospritzer (Parker, Cleaveland) via tubing, from which we controlled the pressure (typically 8-10 psi) and timing of intra-terminal dialysis.

Electrodes for postsynaptic MNTB neurons had a resistance of 2.5-4.5 MΩ with the pipette solution containing (in mM): 110 Cs-methanesulfonate, 30 CsCl, 10 HEPES, 5 EGTA, 5 QX314 and 1 $MgCl_2$ (310 mOsm/l, pH 7.3 adjusted with CsOH). The access resistance was 6–20 MΩ, which was compensated by 80%. EPSCs were recorded from MNTB principal neurons at a holding potential of -70

mV. 100 μM Picrotoxin (100 μM) and strychnine (0.5 μM) were used to block spontaneous inhibitory postsynaptic currents. ω-Conotoxin-GIVA and SNX-482 (Peptide Institute, Osaka) were bath-applied with cytochrome-C (0.1 mg/ml).

Unless otherwise noted all chemicals and salts were from Sigma (St. Louis, MO) or Nacalai (Kyoto, Japan). QX314 was purchased from Almone Labs (Jerusalem). Tetrodotoxin was from Abcam (Cambridge, UK). Electrophysiological recordings were low-pass filtered at 10 kHz and acquired at 50-100 kHz (6052E, National Instruments or Digidata 1320A, Molecular Devices). Data analysis was performed with IgorPro 6.3 (WaveMetrics) using NeuroMatic software (http://www.neuromatic.thinkrandom.com/), or with Excel. Residual series resistance errors were compensated off-line for all evoked EPSCs. The synaptic delay was estimated from the 50% rise of the presynaptic AP to the 20% rise of the EPSC. All values in the text and figures are given as means ± SEM unless otherwise indicated. Statistical comparisons were made using the Student's unpaired t-test unless otherwise noted. $P < 0.05$ was considered as significant.

Time domain deconvolution of AP-evoked EPSCs was performed as describe previously (Neher and Sakaba., 2001; Taschenberger et al., 2005). We avoided use of low-affinity AMPAR antagonists because they inhibit mEPSC, precluding their use as a quantal response template for release rate estimates by deconvolution (see below). Cyclothiazide was also omitted due to its effects on presynaptic Ca^{2+} and K^+ currents (Ishikawa and Takahashi, 2001). For the EPSC deconvolution, matched quantal responses were used at each synapse. Quantal templates were extracted using triple exponential fits to average mEPSC waveform.

Measurements and Analysis of Ca^{2+} Transients

Ca^{2+}-dependent fluorescence transients were recorded using confocal laser scanning and spot detection using an Ultima scanning head (Prairie Technologies) mounted on an Olympus BX61W1 microscope. An acousto-optical tunable filter (AOTF) was used to select the excitation wavelength, amplitude and duration of illumination from 488 (Omicron Laserage) and 594 nm (Cobalt Lasers) diode lasers, for excitation of Oregon Green BAPTA-5N and Alexa 594, respectively. The laser beam was collimated and adjusted to overfill a 1.1 NA 60× objective (LUMFLN60XW, Olympus), thereby forming a. a diffraction-limited illumination spot in the specimen plane. Emitted fluorescence was descaned, and aligned through a 60 µm pinhole, placed on a conjugate image plane (corresponding to ~0.5 Airy units). Fluorescence emission from Oregon Green BAPTA-5N was filtered using a 535/70 nm band pass filter and detected with a gallium arsenide phosphide-based photocathode photomultiplier tube (H7422P, Hamamatsu Photonics). Alexa 594 fluorescence was filtered with a 605LP filter (all filters were from Chroma) and detected with a side-on multi-alkali PMT (3896, Hamamatsu Photonics). Zoom factor was set such that pixel sizes were 80 nm.

Confocal spot-detected fluorescence signals were filtered at 10 kHz using an 8-pole Bessel filter (Frequency Devices), digitized at 100 kHz, then filtered offline at 2 kHz. The jitter in the AP onset was corrected by time-aligning at the AP peak. The magnitude of Ca^{2+} transients was expressed as $\Delta F/F$ of Oregon Green BAPTA-5N fluorescence signal, which was calculated according to the equation:

$$\Delta F/F\ (t) = (F(t) - F_{rest}) / F_{rest} \quad \text{(equation 1)}$$

where F_{rest} is the time averaged (10 ms window) fluorescence before stimulation and $F(t)$ is the time-dependent fluorescence transient. $F(t)$ and F_{rest} values were calculated from the raw PMT signal

by subtracting the signal due to background fluorescence (i.e. due to PMT signal in the dark and that due to auto-fluorescence of tissue).The presence of Ca^{2+} transients was confirmed if the peak amplitude of ΔF/F trace was 3× larger than baseline root mean square, and such traces were further analyzed as shown below. To estimate the amplitude and rise time of Ca^{2+} transients, we fit single or averaged $\Delta F/F$ traces with the following equation (Nielsen et al., 2004), a least-square algorithm implemented in IgorPro:

$$\Delta F/F(t) = A_1\left(1-\exp\left(\frac{t-t_0}{\tau_{rise}}\right)\right)^n\left(A_2\exp\left(\frac{t-t_0}{\tau_{decay1}}\right)+A_3\exp\left(\frac{t-t_0}{\tau_{decay2}}\right)\right) \quad \text{(equation 2)}$$

For kinetic comparison, we selected transients for further analysis according to a rise time criterion of less than 0.5 ms. For spot locations separated by <0.2 um, only the largest trace were chosen within an 0.5 µm window (local maximum). The decay phase of $\Delta F/F$ traces were fitted by a double exponential decay function according to:

$$\Delta F/F(t) = A_1\exp\left(\frac{t-t_0}{\tau_{decay1}}\right)+A_2\exp\left(\frac{t-t_0}{\tau_{decay2}}\right) \qquad \text{(equation 3)}$$

where A_1 and A_2, and τ_{decay1} and τ_{decay2} are the relative amplitudes and time constants, respectively, of the fits. The weighted decay was calculated from the weighted average of the two time constants of a double exponential fit of the Ca^{2+} transient decay $(\tau_{decay1} A_1 + \tau_{decay2} A_2) / (A_1 + A_2)$.

For linescan-based fluorescence detection the acquisition rate for each line was typically 0.9-1.5 ms. To estimate the spatial distribution of Ca^{2+} entry, we calculated $\Delta F/F$ from a 3 ms window during the depolarizing step to 0 mV. This spatial profile of $\Delta F/F$ was calibrated by dividing by the ratio of F_{rest} per pixel and the maximum F_{rest} of the scan. The maximum F_{rest} was assumed to occur when point spread function was fully included within the nerve terminal. This procedure minimized errors caused by the calyx volume smaller than the detection volume and provided a more accurate estimate of the location and size of Ca^{2+} entry sites.

Numerical Simulations of Ca^{2+} Reaction-Diffusion and Vesicular Release

Simulations of Fluorescence Ca^{2+} Transients

We used D3D, a Java-based 3D reaction-diffusion simulator that was developed from earlier simulator implementations (Nielsen et al., 2004; DiGregorio et al., 2007) running on a Windows 7 operating system to calculate the spatiotemporal distribution of $[Ca^{2+}]$ in the vicinity of VGCC clusters at a calyx of Held terminal. Ca^{2+} diffusion and binding with Oregon Green BAPTA-5N and buffers were simulated by numerically integrating differential equations using an explicit finite-difference (Euler) method with a fixed time step and an elementary integration volume (i.e. voxels) (DiGregorio et al., 1999). In order to speed the calculation, we used simulation voxels that were 50 x 50 x 50 nm. The predicted Ca^{2+} transient decay time courses did not differ if the voxel size was reduced to 5 x 5 x 5 nm. The total simulation volume was 0.8 (x) × 0.8 (y) × 1.0 (z) µm, with the Ca^{2+} entry site centered within a surface orthogonal to the z-dimension. The height (z) matches the thickness of the terminal (Sätzler et al., 2002), while the x and y dimensions correspond to the average NND between clusters (Figure 1E), which with reflective boundaries on the four surfaces orthogonal to the x-y dimensions, is mathematically equivalent to periodic symmetrically arranged sources. In some simulations we took

advantage of quarter symmetry in the *x*-*y* plane to reduce the simulation volume by one quarter. The time step was calculated according to a stability criterion determined by the following equation:

$$h = {(3 * D * dt)}/{\partial x^2},$$

where *D* is the diffusion coefficient of the fastest diffusant, *dx* the voxel width and *h* the stability factor (0.1-0.4). Ca^{2+} extrusion via active transport was included on both *z* surfaces at a rate of 400 Ca^{2+}/s (Helmchen et al, 1997).

To model Ca^{2+} entry, the simulation volume contained a single 100 × 100 nm Ca^{2+} entry site. The total I_{Ca}, equivalent to the summed current from open VGCCs within a cluster, was homogenously distributed over the Ca^{2+} entry site, a size mimicking the average VGCC cluster area (Figure 2C). The total Ca^{2+} influx was varied by adjusting the equivalent number of open VGCCs to reproduce the amplitude of measured Ca^{2+} transient (Figures 4A and 4B). The time course of whole-terminal I_{Ca} in response to a voltage command waveform of a presynaptic AP (Figure 3F) was used as the waveform of Ca^{2+} entry. Because developmental shortening in the presynaptic AP results in a different Ca^{2+} driving force during the AP, we estimated the driving force for P7 and P14 conditions as the difference between the Ca^{2+} reversal potential (+45 mV, Sheng et al, 2012) and the potential of the AP waveform at the time of maximal I_{Ca} (Figure 3F). The computed Ca^{2+} driving force along with the single VGCC conductance (Sheng et al., 2012) resulted in a single VGCC current equaling 0.28 pA for P7 and 0.35 pA for P14 conditions. The resulting I_{Ca} had a half-duration of 0.28 ms for P7 and 0.19 ms for P14 conditions.

The concentration of Ca^{2+}-bound Oregon Green BAPTA-5N was converted to a fluorescence change ΔF/F as described by equation 6 in DiGregorio et al. (1999). To compare to experimental data, ΔF/F values were weighted according to a 3D Gaussian function matching the lateral and axial full width at half maximal of the measured confocal point spread function (Figure S3). The Gaussian volume was positioned in different locations within the simulation volumes by adding an *x*, *y*, or *z* offset to the 3D function (Figures S4D and S4I). Point spread function locations that produced transients with fast rise times (<0.5 ms) were then averaged together to produce a final simulated ΔF/F transient (0.1 mM EGTA) that was compared directly to data. The same spot locations were used to produce the average simulated ΔF/F transient in 2 mM EGTA. For confocal spot locations corresponding to the synaptic surface, the point spread function was centered 100 nm from the *z* surface containing the Ca^{2+} entry site. Spatial dependence along the synaptic face (parallel scan) and perpendicular to the face was achieved by displacing the center of the point spread function in the *x* or *z* dimension, respectively (Figure S4). Simulation parameters are summarized in Table S1.

Simulations of Nanoscale [Ca^{2+}] and Vesicular Release

To simulate Ca^{2+} reaction-diffusion near the vicinity of VGCC clusters on the nanoscale, we used the D3D simulation environment with a smaller voxel size (5 nm cubes). To reduce the total simulation time, we used a smaller simulation volume of 0.5 (*x*) × 0.5 (*y*) × 1.0 (*z*) μm. We confirmed that this smaller volume generated a similar result to that of the larger volume and therefore had no effect on our PCD estimate (data not shown). We also omitted the Ca^{2+} extrusion from these simulations, because the Ca^{2+} extrusion rate is so slow that it does not make significant contribution during the 10 ms simulation duration. For the P7 simulations, we assumed a VGCC open probability = 0.25 during a single AP

(Sheng et al, 2012) estimated using single channel recordings. For the P14 simulations, open probability was scaled down by 30% to 0.175 to account for the shorter AP duration at P14 (Yang and Wang, 2006).

For simulations of fixed VGCC open patterns (Figures 6 and 8), we modeled only the open VGCCs during an AP. The open channel number and density is assumed to equal that of gold particles per cluster of La1 samples, because the VGCC open probability is similar to the labeling efficiency of La1 (19%). We used a grid distribution of the VGCCs with a NND of 35 nm, which is similar to the NND of the gold particles measured in the La1 samples (37 nm). In some cases we directly used the immunogold particle locations of La1 clusters to define the open VGCC positions. The transmitter release rate was calculated using a 5-state Ca^{2+}-dependent release model for developing rats (Kochubey et al, 2009), together with the simulated $[Ca^{2+}]$ time course ($[Ca^{2+}](t)$) computed at each 5 nm voxel in Igor using the Euler method. The vesicular release probability (P_v) was calculated from the integral of the release rate. EGTA-inhibition of P_v was estimated by examining the simulated P_v at a particular voxel when simulated $[Ca^{2+}](t)$ waveforms in 0.1 or 10 mM EGTA. For each cluster we estimated the PCD, defined as the average distance between the voxels exhibiting experimental EGTA-inhibition values (Figure 5F) and the nearest VGCC. Contour lines were drawn using the implemented function of Igor. P_v for each VGCC cluster was the mean P_v over voxels at the PCD in the presence of 0.1 mM EGTA. For the analysis of release kinetics, we used results from the fixed pattern of VGCC opening simulations. Similar results were obtained using other age-dependent models in mice (Wang et al., 2008).

For the simulations with stochastic patterns of VGCC opening (Figure 7), we used a grid distribution of VGCC with a NND of 25 nm, similar to the gold particle NND of La2. Different patterns of open VGCCs were generated on each trial by assuming that the each VGCC was independent and had an equal open probability during AP (specific for that each age). We computed $[Ca^{2+}](t)$ waveforms at each voxel for each trial, which were then used to estimate the fraction of sensors in the release state at each voxel per trial. P_v was then calculated from the average fraction of sensors in the release state across 50 trials. EGTA-inhibition of P_v was then calculated from the fractional reduction in P_v in the 0.1 mM and 10 mM EGTA simulations.

Simulation parameters are summarized in Table S2. The k_{on} value reported for EGTA by Naraghi (1997; 2.7×10^6 $M^{-1}s^{-1}$ at pH 7.2) is one order of magnitude slower than that by Nägerl et al., (2000; 1.0×10^7 $M^{-1}s^{-1}$ at pH 7.3, also see Neher, 1986). We adopted the latter value because the magnitude of EPSC-inhibition by 10 mM EGTA was similar to that by 0.5-1 mM BAPTA (k_{on}, 4.5×10^8 $M^{-1}s^{-1}$) at P14 calyces (data not shown), suggesting 10-20 times, not 170 times (Naraghi, 1997), difference in k_{on} between EGTA and BAPTA.

SUPPREMENTAL REFERENCES

Allbritton, N. L., Meyer, T., and Stryer, L. (1992). Range of messenger action of calcium ion and inositol 1,4,5- trisphosphate. Science *258*, 1812-1815.

Bollmann, J.H., and Sakmann, B. (2005). Control of synaptic strength and timing by the release-site Ca^{2+} signal. Nat Neurosci *8*, 426-434.

Budisantoso, T., Harada, H., Kamasawa, N., Fukazawa, Y., Shigemoto, R., and Matsui, K. (2013). Evaluation of glutamate concentration transient in the synaptic cleft of the rat calyx of Held. J Physiol *591*, 219-239.

DiGregorio, D.A., Peskoff, A., and Vergara, J.L. (1999). Measurement of action potential-induced presynaptic calcium domains at a cultured neuromuscular junction. J Neurosci *19*, 7846-7859.

DiGregorio, D.A., Rothman, J.S., Nielsen, T.A., and Silver, R.A. (2007). Desensitization properties of AMPA receptors at the cerebellar mossy fiber granule cell synapse. J Neurosci *27*, 8344-8357.

Edmonds, B., Reyes, R., Schwaller, B., and Roberts, W.M. (2000). Calretinin modifies presynaptic calcium signaling in frog saccular hair cells. Nat Neurosci *3*, 786-790.

Faas, G.C., Schwaller, B., Vergara, J. L., and Mody, I. (2007). Resolving the fast kinetics of cooperative binding: Ca^{2+} buffering by Calretinin. Plos Biolgy *5*, e311.

Fujimoto, K. (1995). Freeze-fracture replica electron microscopy combined with SDS digestion for cytochemical labeling of integral membrane proteins. Application to the immunogold labeling of intercellular junctional complexes. J Cell Sci *108*, 3443-3449.

Gabso, M., Neher, E., and Spira, M. E. (1997). Low mobility of the Ca^{2+} buffers in axons of cultured Aplysia neurons. Neuron *18*, 473-481.

Helmchen, F., Borst, J.G., and Sakmann, B. (1997). Calcium dynamics associated with a single action potential in a CNS presynaptic terminal. Biophys J *72*, 1458-1471.

Holderith, N., Lorincz, A., Katona, G., Rozsa, B., Kulik, A., Watanabe, M., and Nusser, Z. (2012). Release probability of hippocampal glutamatergic terminals scales with the size of the active zone. Nat Neurosci *15*, 988-997.

Indriati, D.W., Kamasawa, N., Matsui, K., Meredith, A.L., Watanabe, M., and Shigemoto, R. (2013). Quantitative localization of $Ca_v2.1$ (P/Q-type) voltage-dependent calcium channels in Purkinje cells: somatodendritic gradient and distinct somatic coclustering with calcium-activated potassium channels. J Neurosci *33*, 3668-3678.

Ishikawa, T., and Takahashi, T. (2001). Mechanisms underlying presynaptic facilitatory effect of cyclothiazide at the calyx of Held of juvenile rats. J Physiol *533*, 423-431.

Kochubey, O., Han, Y., and Schneggenburger, R. (2009). Developmental regulation of the intracellular Ca^{2+} sensitivity of vesicle fusion and Ca^{2+}-secretion coupling at the rat calyx of Held. J Physiol *587*, 3009-3023.

Lin, K.H., Oleskevich, S., and Taschenberger, H. (2011). Presynaptic Ca^{2+} influx and vesicle exocytosis at the mouse endbulb of Held: a comparison of two auditory nerve terminals. J Physiol *589*, 4301-4320.

Loukanov A., Kamasawa N., Danev R., Shigemoto R., and Nagayama K. (2010). Immunolocalization of multiple membrane proteins on a carbon replica with STEM and EDX. Ultramicroscopy *110*, 366-374,

Masugi-Tokita, M., and Shigemoto, R. (2007). High-resolution quantitative visualization of glutamate and GABA receptors at central synapses. Curr Opin Neurobiol *17*, 387-393.

Masugi-Tokita, M., Tarusawa, E., Watanabe, M., Molnár, E., Fujimoto, K., and Shigemoto, R. (2007). Number and density of AMPA receptors in individual synapses in the rat cerebellum as revealed by SDS-digested freeze-fracture replica labeling. J Neurosci *27*, 2135-2144.

Miyazaki, T., Yamasaki, M., Hashimoto, K., Yamazaki, M., Abe, M., Usui, H., Kano, M., Sakimura, K., and Watanabe, M. (2012). $Ca_v2.1$ in cerebellar Purkinje cells regulates competitive excitatory

synaptic wiring, cell survival, and cerebellar biochemical compartmentalization. J Neurosci *32*, 1311-1328.

Nägerl, U. V., Novo, D., Mody, I., and Vergara, J. L. (2000). Binding kinetics of calbindin-D(28k) determined by flash photolysis of caged Ca^{2+}. Biophys J *79*, 3009-3018.

Naraghi, M. (1997) T-jump study of calcium binding kinetics of calcium chelators. Cell Calcium *22*, 255-268

Naraghi, M., and Neher, E. (1997). Linearized buffered Ca^{2+} diffusion in microdomains and its implications for calculation of [Ca^{2+}] at the mouth of a calcium channel. J Neurosci *17*, 6961-6973.

Nakamura, T., Yamashita, T., Saitoh, N., and Takahashi, T. (2008). Developmental changes in calcium/calmodulin-dependent inactivation of calcium currents at the rat calyx of Held. J Physiol *586*, 2253-2261.

Neher, E. (1986) Concentration profiles of intracellular calcium in the presence of a diffusible chelator. Exp Brain Res *14*, 80-96.

Nielsen, T.A., DiGregorio, D.A., and Silver, R.A. (2004). Modulation of glutamate mobility reveals the mechanism underlying slow-rising AMPAR EPSCs and the diffusion coefficient in the synaptic cleft. Neuron *42*, 757-771.

Sakaba, T., and Neher, E. (2001). Quantitative relationship between transmitter release and calcium current at the calyx of Held synapse. J Neurosci *21*, 462-476.

Sätzler, K., Söhl, L.F., Bollmann, J.H., Borst, J.G.G., Frotscher, M., Sakmann, B., and Lübke, J.H. (2002). Three-dimensional reconstruction of a calyx of Held and its postsynaptic principal neuron in the medial nucleus of the trapezoid body. J Neurosci *22*, 10567-10579.

Schmidt, H., Schwaller, B. and Eilers, J. (2005) Calbindin D28k targets myo-inositol monophosphatase in spines and dendrites of cerebellar Purkinje neurons. Proc Natl Acad Sci U S A *102*, 5850–5855

Sheng, J., He, L., Zheng, H., Xue, L., Luo, F., Shin, W., Sun, T., Kuner, T., Yue, D.T., and Wu, L.G. (2012). Calcium-channel number critically influences synaptic strength and plasticity at the active zone. Nat Neurosci *15*, 998-1006.

Takahashi,T., Hori, T., Nakamura, Y., and Yamashita,T. (2012). Patch-clamp recording method in slices for studying presynaptic mechanisms. In Patch Clamp Techniques, Y Okada, ed. (Springer) pp. 137-145.

Taschenberger, H., Leão, R.M., Rowland, K.C., Spirou, G.A., and von Gersdorff, H. (2002). Optimizing synaptic architecture and efficiency for high-frequency transmission. Neuron *36*, 1127-1143.

Taschenberger, H., Scheuss, V., and Neher, E. (2005). Release kinetics, quantal parameters and their modulation during short-term depression at a developing synapse in the rat CNS. J Physiol *568*, 513-537.

Wang, L.Y., Neher, E., and Taschenberger, H. (2008). Synaptic vesicles in mature calyx of Held synapses sense higher nanodomain calcium concentrations during action potential-evoked glutamate release. J Neurosci *28*, 14450-14458.

Xu, T., Naraghi, M., Kang, H., and Neher, E. (1997). Kinetic studies of Ca^{2+} binding and Ca^{2+} clearance in the cytosol of adrenal chromaffin cells. Biophys J *73*, 532-545.

Yang, Y.M., and Wang, L.Y. (2006). Amplitude and kinetics of action potential-evoked Ca^{2+} current and its efficacy in triggering transmitter release at the developing calyx of Held synapse. J Neurosci *26*, 5698-5708.
